# Regiochemistry of cyclocondensation reactions in the synthesis of polyazaheterocycles

**DOI:** 10.3762/bjoc.13.29

**Published:** 2017-02-10

**Authors:** Patrick T Campos, Leticia V Rodrigues, Andrei L Belladona, Caroline R Bender, Juliana S Bitencurt, Fernanda A Rosa, Davi F Back, Helio G Bonacorso, Nilo Zanatta, Clarissa P Frizzo, Marcos A P Martins

**Affiliations:** 1 IFSul Campus de Pelotas, Instituto Federal de Educação, Ciência e Tecnologia Sul-Rio-Grandense, 96.015-360, Pelotas, RS, Brazil; 2 Núcleo de Química de Heterociclos (NUQUIMHE), Departamento de Química, Centro de Ciências Naturais e Exatas, Universidade Federal de Santa Maria, 97.105-900, Santa Maria, RS, Brazil; 3 Departamento de Química, Centro de Ciências Exatas, Universidade Estadual de Maringá, 87020-900,Maringa, PR, Brazil; 4 Departamento de Química, Universidade Federal de Santa Maria, 97105-900, Santa Maria, RS, Brazil

**Keywords:** DFT-B3LYP, polyazaheterocycles, pyrazinone, pyrido[1,2-*a*]pyrimidinone, pyrimido[1,2-*a*]benzimidazole, quinoxalinone, thiazolo[3,2-*a*]pyrimidinone

## Abstract

The syntheses of several polyazaheterocycles are demonstrated. The cyclocondensation reactions between β-enaminodiketones [CCl_3_C(O)C(=CNMe_2_)C(O)-CO_2_Et] and aromatic amidines resulted in glyoxalate-substituted pyrido[1,2-*a*]pyrimidinone, thiazolo[3,2-*a*]pyrimidinone and pyrimido[1,2-*a*]benzimidazole. Pyrazinones and quinoxalinones were obtained through the reaction of these glyoxalates with ethylenediamine and 1,2-phenylenediamine derivatives. On the other hand, the reaction of glyoxalates with amidines did not lead to the formation of imidazolones, but rather *N*-acylated products were obtained. All the products were isolated in good yields. DFT-B3LYP calculations provided HOMO/LUMO coefficients, charge densities, and the stability energies of the intermediates, and from these data it was possible to explain the regiochemistry of the products obtained. Additionally, the data were a useful tool for elucidating the reaction mechanisms.

## Introduction

Various syntheses of polyazaheterocycles are described in the literature because they are important components for the preparation of bioactive molecules [1–3]. One of the most important synthetic methods towards compounds containing nitrogen atoms in the ring junction represents the cyclocondensation reaction [4]. Pyrido[1,2-*a*]pyrimidinones [5], thiazolo[3,2-*a*]pyrimidinones [6], and pyrimido[1,2-*a*]benzimidazole [7] are examples of polyazaheterocycles obtained through the reaction of 1,3-dielectrophiles and 1,3-dinucleophiles. Appropriately functionalized heterocycles can also lead to the formation of polyazaheterocycles. Our research group has reported the synthesis of ethyl 5-carbonylpyrimidine-4-carboxylates from unsymmetrical enaminodiketones and amidines and their application in the preparation of pyrimido[4,5-*d*]pyridazin-8(7*H*)-ones [8]. One of the most common approaches used for the synthesis of pyrazinones [9–10] and quinoxalinone [11–12] cores is the cyclocondensation reaction between 1,2-dicarbonyl compounds and 1,2-diamines. In this manner, Zamcova et al. [13] reported the synthesis of imidazo[1,2]heteroarylglyoxylates, which involved the cyclocondensation of 1,2-dicarbonyl compounds with ethylenediamine and 1,2-phenylenediamine and they obtained polyazaheterocycles with pyrazinone and quinoxalinone cores.

Although there is a wide range of papers reporting on cyclocondensation reactions, only few authors have discussed the regiochemistry of the products [11]. Saiz et al. [14] synthesized 2-hydrazolyl-4-thiazolidinones with the aid of semi-empirical calculations (PM3 method). Through HOMO/LUMO energies, orbital coefficients, and charge distribution, a mechanism was proposed explaining the products observed. The authors claimed that the HOMO/LUMO energy gap is small and that the reaction between thiosemicarbazone and benzyl is kinetically favored, probably controlled by the frontier orbital component. Furthermore, calculations of HOMO and LUMO frontier orbital coefficients were used to prove the regiochemistry in cycloaddition reactions [15–16].

The main objective of this work has been the synthesis of a series of polyazaheterocycles through cyclocondensation reactions between a β-enaminodiketone and several 1,3-dinucleophiles. Due to the versatility of the precursors, a wide range of compounds was expected. Hence, DFT-B3LYP quantum-chemical calculations were used to understand the regiochemistry of the obtained products.

## Results and Discussion

β-Enaminodiketone **1**, which is a key precursor for the synthesis of polyazaheterocyclic compounds, was synthesized by methods previously described by our research group [17–20]. This compound is a highly versatile precursor, because it comprises four distinct electrophilic centers which can be attacked by nucleophiles (positions 2, 4, 5, and 6, see Table 1).

**Table 1:** Reaction of β-enaminodiketone **1** with aromatic amidines **2**–**4**.

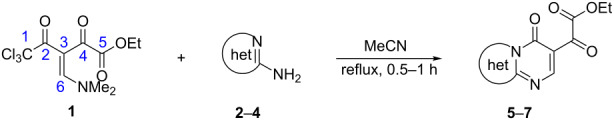			

Entry	Amidine	Product	Yield (%)^a^

1^b^	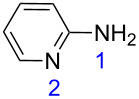 **2**	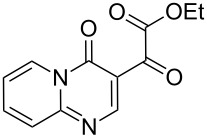 **5**	80
2^b^	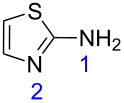 **3**	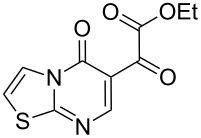 **6**	86
3^c^	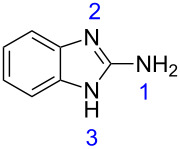 **4**	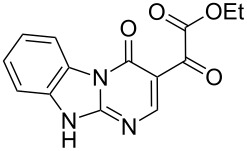 **7**	56

^a^Isolated yield. ^b^Ideal condition = 0.5 h. ^c^Ideal condition = 1 h.

On the other hand, the non-symmetrical dinucleophiles **2–4** (Table 1) employed in this work can lead to different reaction pathways, thus affording different products. Initially, the reaction between β-enaminodiketone **1** and 2-aminopyridine (**2**) (Table 1) was tested in acetonitrile at 25 °C and the progress of the reaction was monitored by thin-layer chromatography (TLC). Full conversion was achieved after 3 h and product **5** was isolated in a yield of 74%. When the reaction was repeated under reflux conditions, the time to reach conversion was reduced to 30 min and product **5** was isolated in 80% yield (Table 1). Other solvents such as ethanol, tetrahydrofuran, acetone, or ethyl acetate were also tested under reflux conditions but were not as efficient as acetonitrile. Generally refluxing **1** and **2** in either solvent needed longer reaction times in most cases (0.5, 3, 1, and 1 h, respectively for ethanol, tetrahydrofuran, acetone, and ethyl acetate) and throughout lower yields of **5** were obtained (54, 74, 77, and 75%, respectively).

With the best conditions (refluxing acetonitrile, 30 min) at hand, compound **1** was next reacted with amidines **3** and **4**. While compound **6** was isolated in a good yield of 86% under these conditions, product **7** was isolated in only 56% yield after prolonged (1 h) reaction time (Table 1). The formation of product **7** was observed by precipitation. After completion of the reaction, the solvent was evaporated and the residue purified by column chromatography. All products **5**–**7** were obtained in a highly regioselective manner and in good yields. Analogues of **5**–**7** with a carbonyl substituent have been previously reported from the cyclocondensation reaction between their respective dinucleophiles and diethyl ethoxymethylenemalonate [21–25].

Theoretical DFT-B3LYP calculations were performed to elucidate the reaction mechanism. From the energy minimization calculations the HOMO/LUMO coefficient and charge density data were determined and the results showed that the C(6) (β-carbon) of **1** had the largest LUMO coefficient (Table 2), thus indicating its soft electrophilic character. The carbonyl group C(2) had a lower value than C(6) and a significantly higher value than the ester carbonyl C(5). This is probably due to the inductive effect (−I) by the chlorine atoms near to C(2). Lastly, C(5) had the lowest LUMO coefficient value of all electrophilic centers present in the enaminodiketone. One reason for this is the mesomeric effect (+M), which is caused by the delocalization of electrons from the oxygen atom present in the ethoxy part. These data indicate that the first nucleophilic attack takes place on C(6) (β-carbon), while the second attack occurs on C(2).

**Table 2:** LUMO coefficients and charge densities for the selected atoms in **1** obtained by DFT-B3LYP calculations.

LUMO (a.u.)	−0.076			
Atoms	C2	C4	C5	C6
LUMO coeff.	0.171	0.024	0.001	0.225
Charge density	0.249	0.199	0.309	−0.040

The corresponding calculations for nucleophiles **2–4** showed that the sp^3^ hybridized nitrogen atoms had the largest HOMO coefficients, followed by the nitrogen atoms (sp^2^) of the ring (Table 3). These HOMO coefficients designate the first and second nucleophilic attack, respectively.

**Table 3:** HOMO coefficients and charge densities for the selected atoms in **2–4** obtained by DFT-B3LYP calculations.

Compounds	**2**		**3**		**4**		

HOMO (a.u.)	−0.221		−0.219		−0.210		
Atoms	N1	N2	N1	N2	N1	N2	N3
HOMO coeff.	0.238	0.108	0.206	0.127	0.156	0.033	0.142
Charge density	−0.261	−0.187	−0.246	−0.201	−0.259	−0.148	−0.261

The order of charge density values for the selected carbon atoms in **1** was as follows: C(5) > C(2) > C(4) > C(6). On the other hand, the charge density values obtained for the investigated atoms in compounds **2–4** were higher for the sp^2^ hybridized nitrogen of the ring. Considering the products formed in the synthesis, as well as the calculation values, it was possible to conclude that the reaction was thermodynamically favored. Small differences were observed between the HOMO/LUMO energies of electrophiles and nucleophiles (Table 2 and Table 3), and the reactions were controlled by frontier molecular orbitals rather than by charge density. Analogous calculations using the PM3 method for cyclocondensation reactions were performed by Saiz et al. [14] for the formation of thiazolidinones and by Komarov et al. [26] for triazoles.

**Scheme 1:**
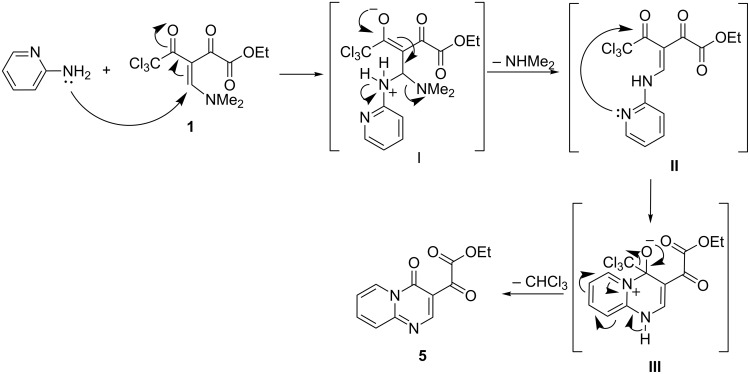
Mechanism proposed for the formation of compound **5**.

Based on the DFT-B3LYP calculations, the formation of products **3–7** can be explained through the following steps (Scheme 1): (i) attack by the NH_2_ nucleophile of **2** on the β-carbon of **1** resulting in adduct **I**; (ii) elimination of the NMe_2_ group from intermediate **I** under formation of **II**; (iii) the second nucleophilic attack, which is promoted by the nitrogen atom of the pyridine ring, on the carbonyl carbon atom adjacent to the CCl_3_ group leads to intermediate **III**; (iv) the π-bond formation causes the elimination of the CCl_3_ group, followed by aromatization finally generates product **5** (Scheme 1). The trichloromethyl (CCl_3_) substituent as a leaving group in β-alkoxyvinyl trichloromethyl ketones has been previously used by us for the synthesis of similar heterocycles [27–28].

Products **5–7** are considered to be very attractive building block in the synthesis of heterocycles, because they have three different electrophilic carbonyl groups. This is expected to lead to different reaction pathways and thus, diverse products. However, the amide carbonyl of these compounds is considered to be less reactive, as its modification results in the loss of heterocyclic aromaticity.

However, aiming at the synthesis of new heterocycles, we next investigated the reaction of compounds **5–7** with different 1,2-diamines. Initially, the reaction between compound **5** and ethylenediamine (**8a**) was tested. Due to precipitation of the product during the course of the reaction, the conversion was analyzed by ^1^H NMR.

Under initial conditions (acetonitrile, 25 °C, 1 h) 67% of the reactants were converted to product **9a** which could be increased to 71% by a longer reaction time of 2 h. When the reaction was performed at reflux temperature of acetonitrile for 1 h, a conversion of 83% could be achieved. Finally, a complete consumption of the starting material was achieved in refluxing acetonitrile for 2 h. Other solvents (ethanol, THF, acetone, ethyl acetate) were also tested at reflux temperature for 2 h and conversion rates of 95, 44, 41, and 41%, respectively, were obtained.

The best reaction conditions were used for the subsequent cyclocondensation reactions between compounds **5–7** and various diamines **8a–f**. The reaction time ranged from 2 to 24 h and the pyrazinone and quinoxalinone derivatives **9a–f**, **10c–f**, **11a–e** were obtained in medium to high yield (see Table 4 for substituents originating from the diamino components **8a**–**f** and Table 5 for the reaction products).

**Table 4:** Substituents R in products **9a–f**, **9b'**, **9e'**, **9f'**, **10c–f**, **10e'**, **10f'**, **11a–e** and **11b'** originating from the diamine.

R	**a**	**b**	**c**	**d**	**e**	**f**

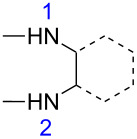	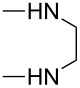	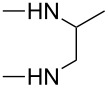	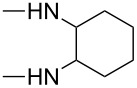	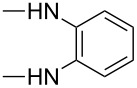	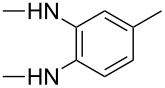	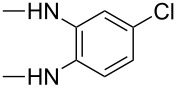

**Table 5:** Synthesis of compounds **9a–f**, **9b'**, **9e'**, **9f'**, **10c–f**, **10e'**, **10f'**, **11a–e** and **11b'**.

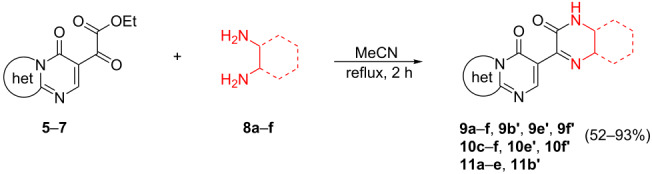					

Entry	α-Ketoester	1,2-Diamine	Products	Yield (%)^c^	Isomers (%)

1	**5**	**8a**	**9a**	83	–
2	**5**	**8b**	**9b**:**9b’**	68	27:73
3	**5**	**8c**	**9c**	72	–
4	**5**	**8d**	**9d**	87	–
5^a^	**5**	**8e**	**9e**:**9e’**	65	44:56
6^a^	**5**	**8f**	**9f**:**9f’**	66	55:45
7	**6**	**8c**	**10c**	77	–
8	**6**	**8d**	**10d**	93	–
9	**6**	**8e**	**10e**:**10e’**	70	42:58
10	**6**	**8f**	**10f**:**10f’**	66	48:52
11	**7**	**8a**	**11a**	78	–
12	**7**	**8b**	**11b**:**11b’**	89	50:50
13	**7**	**8c**	**11c**	91	–
14^b^	**7**	**8d**	**11d**	52	–
15^b^	**7**	**8e**	**11e**:**11e’**	56	48:52

^a^Reaction time of 16 h; ^b^Reaction time of 24 h. ^c^Isolated product.

All products precipitated from the reaction mixtures and could be collected by simple filtration. The products **9a–f**, **10c–f** were purified by simple washing with ethyl acetate (3 × 1 mL) and compounds **11a–e** were washed with acetonitrile (1 × 1 mL). The reactions done with non-symmetric dinucleophiles **8b**, **8e**, and **8f** furnished isomeric product mixtures.

Data obtained from DFT-B3LYP theoretical calculations showed that in the three α-ketoesters **5–7**, the carbonyl of the ketone has a larger LUMO coefficient and a higher charge density than the ester carbonyl (Table 6). These values indicate that the first and second nucleophilic attack on the 1,2-dicarbonyl should take place at the C_ketone_ and the ester carbonyl, respectively.

**Table 6:** . LUMO coefficients and charge densities for the selected atoms in compounds **5–7** obtained by DFT-B3LYP calculations.

Compounds	**5**		**6**		**7**	

LUMO (a.u.)	−0.101		−0.095		−0.081	
Atoms	C_ketone_	C_ester_	C_ketone_	C_ester_	C_ketone_	C_ester_
LUMO coeff.	0.099	0.009	0.111	0.007	0.120	0.007
Charge density	0.233	0.293	0.231	0.293	0.230	0.297

For product **8b**, the HOMO coefficient was larger for N2 (Table 7). This indicates that this N2 is involved in the first nucleophilic attack on the ketone carbon atom of substrates **5–7** (most electrophilic center). The subsequent cyclization then occurs through the attack of N1 from **8b** on the ester carbon justifying the formation of product **9b’** in larger quantities than the isomeric product **9b** (see structures in the Supporting Information File 1). Additionally, it was observed that the nitrogen atom farthest from the substituent is the most nucleophilic. The better conjugation of methyl (hyperconjugation effect) and chlorine (+M effect) substituents in the *para*-position could be a reason for the decrease in the N1 nucleophilicity.

**Table 7:** HOMO coefficients and charge densities for the selected atoms in the precursors **8a–f** obtained by DFT-B3LYP calculations.

Compound	**8a**		**8b**		**8d**	

HOMO (a.u.)	−0.232		−0.229		−0.196	
Atoms	N1	N2	N1	N2	N1	N2
HOMO coeff.	0.175	0.006	0.307	0.021	0.162	0.162
Charge density	−0.327	−0.320	−0.334	−0.331	−0.314	−0.314

Nevertheless, the HOMO coefficient values found for the substituted 1,2-phenylenediamine were lower than those found for 1,2-phenylenediamine (**8d**) without a substituent. Similarly, for **8b** smaller HOMO coefficients are obtained than for the nitrogen atoms of ethylenediamine (**8a**) (see Table 7). For the nucleophiles **8e** and **8f**, the calculations showed similar HOMO coefficients (Supporting Information File 1, Table S1) for both nitrogen atoms, thus indicating no preference in the nucleophilic attack. This explains why the proportions of products containing these fragments are close to 1:1 (e.g., **9e** and **9e’**, **9f** and **9f’**, **10e** and **10e’**, **10f** and **10f’**, and **11e** and **11e’**). As can be seen in the literature, the use of **8e** [29] and **8f** [30] in the synthesis of quinoxalinones leads to the formation of isomeric mixtures.

To determine the stability of the isomers, a calculation was done for all products that were obtained as a mixture of isomers. The energy values of the individual products are shown in Table 8. The values indicate a small energy difference between the isomers, ranging from 0.53 to 0.76 kcal mol^−1^ for the phenylenediamine derivatives (**e**,**e**’;**f**,**f**’), and from 1.44 to 1.47 kcal mol^−1^ for compounds containing 1,2-diaminopropane (**b**,**b**’) in the structure. These data suggest that there is no formation of a preferential isomer, thus corroborating the experimental results.

**Table 8:** Energies for isomeric products obtained from DFT-B3LYP calculations.

Most stable isomer	Least stable isomer	Δ*E* for isomers (kcal mol^−1^)

**9b**	**9b’**	1.47
**9e**	**9e’**	0.74
**9f**	**9f’**	0.56
**10e**	**10e’**	0.76
**10f**	**10f’**	0.58
**11b**	**11b’**	1.44
**11e**	**11e’**	0.53

In order to synthesize imidazolones, reactions of the previously obtained α-ketoesters **5** and **6** were done with amidines **8g**,**h**. The reaction between **5** and **8g** was tested to achieve the optimal conditions. The use of acetonitrile or ethanol as the solvent (at 25 °C or reflux) in the presence of bases such as potassium carbonate (K_2_CO_3_) or sodium ethoxide (CH_3_CH_2_ONa), and with reaction times ranging from 0.5 to 20 h, was not effective in forming the imidazolone of interest. The only product observed was the acylated amidine generated through a nucleophilic addition of the amidine to the ester carbonyl. For the latter compound the best result was obtained for the reaction in ethanol with CH_3_CH_2_ONa as the base at 25 °C for 0.5 h.

The results from the condensation reactions of **5** and **6** with acetamidine (**8g**) and benzamidine (**8h**) are collected in Table 9. As before, the reaction products **12g**,**h** and **13g**,**h** precipitated from the reaction mixture and were purified by simple washing with ethyl acetate (3 × 1 mL) and distilled water (2 × 1 mL). Thus the products were obtained by the transformation of an ester into an amide and occurred similarly to those synthesized by Andreichikov et al. [31] without subsequent cyclization.

**Table 9:** Synthesis of compounds **12g**,**h** and **13g**,**h**.

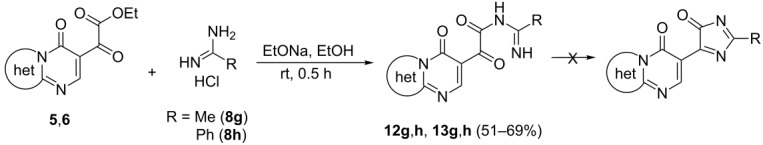				

Entry	α-Ketoester	Amidine	Products	Yield (%)^a^

1	**5**	**8g**	**12g**	67
2	**5**	**8h**	**12h**	66
3	**6**	**8g**	**13g**	69
4	**6**	**8h**	**13h**	51

^a^Isolated product.

The experimental results suggest that the first step in the reaction mechanism involves a nucleophilic attack on the ester carbonyl, which leads to the formation of an amide intermediate. This means that the amidine does not attack the (more reactive) ketone carbonyl as expected, which would lead to an intermediate imine. Since the more reactive center of the dinucleophile does not react with the more reactive center of the dielectrophile it can be concluded that the reaction is thermodynamically controlled rather than kinetically controlled. Thus, the stability of the intermediates formed in the reaction should be considered to be more important than the reactivity of the site.

Knowing that the formation of imine and amide intermediates in this reaction proceeds through the elimination of one water and one ethanol molecule as cleavage products, direct energetic comparisons were performed between each intermediate/cleavage product pair. The DFT-B3LYP calculation method showed that the amide/ethanol pair are by −8.33 kcal·mol^−1^ and −6.79 kcal·mol^−1^ more stable than the imine/water pair for the ethylenediamine and acetamidine derivatives, respectively (Table 10). These results, which are in agreement with the experimental results obtained for the reaction between α-ketoester and acetamidine (Table 9), indicate that the first stage of pyridazinone and quinoxalinone formation also proceeds through an amide intermediate.

**Table 10:** . Energies calculated by DFT-B3LYP for intermediates and cleavage products.^a^

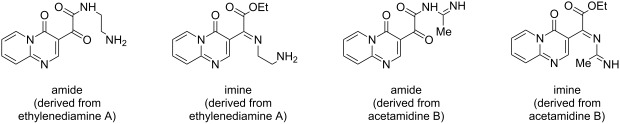			

Intermediate/cleavage product (A)	∆*E* (kcal·mol^−1^)	Intermediate/cleavage product (B)	∆*E* (kcal·mol^−1^)

amide/ethanol	−8.33	amide/ethanol	−6.79
imine/water	0.00	imine/water	0.00

^a^Δ*E* = (*E*_(amide+ethanol_ − *E*_(imine+water)_).

**Scheme 2:**
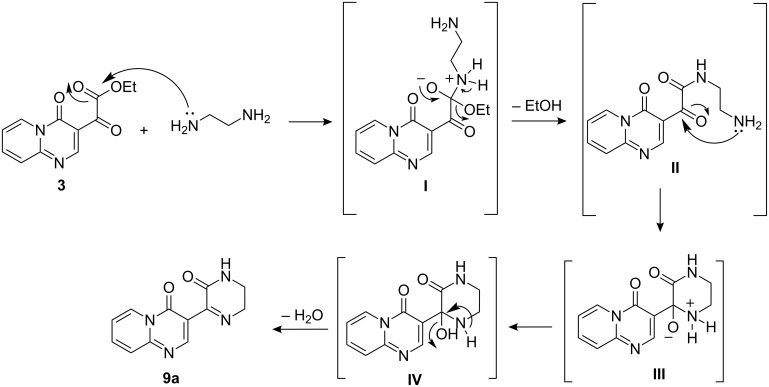
Mechanism proposed for the formation of compound **9a**.

The formation of the products **9a–f**, **10c–f** and **11a–e** can be explained via the mechanism presented in Scheme 2, which details compound **9a**. Initially, (i) the nucleophilic attack of the ethylenediamine nitrogen on the ester carbonyl carbon atom leads to intermediate **I**; (ii) the elimination of one molecule of ethanol leads to the intermediate amide **II**; (iii) the attack of the second nitrogen atom in the ethylenediamine part from **8a** leads to intermediate **III**; (iv) a prototropism occurs which leads to the formation of **IV**; finally, (v) the elimination of a water molecule results in the product **9a**. The reaction mechanism for the formation of the acylated amidines **12g**,**h** and **13g**,**h** is known and comprises the first two steps shown in Scheme 2.

In a multicomponent reaction between an α-ketoacid, a diamine such as **8d**, an aldehyde, and an isonitrile Nixey et al. [32] observed an amide intermediate prior to the formation of the heterocyclic quinoxalinone. An intermediate amide was also observed by Sherman et al. [33] in the reaction between an α-ketoacid, thiophene-2-glyoxylic acid, and *N*-(2-amino-4-nitrophenyl)acetamide, in accordance with the mechanism proposed in this work.

**Figure 1:**
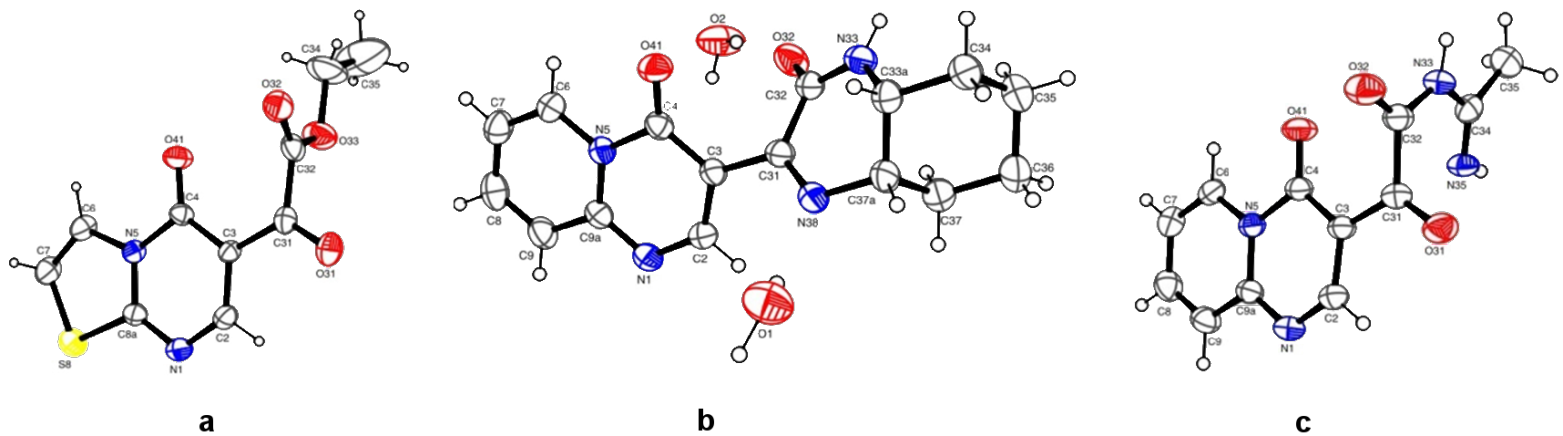
ORTEP plot of **6**, **9c**, and **12g** with the thermal ellipsoids drawn at the following probability levels: (a) 20%, (b) 50%, and (c) 40%.

To rationalize why the cyclization reaction between the amidines **8g**,**h** and the α-ketoester did not occur, the HOMO coefficient and charge densities for the nitrogens of the amidine were determined using computational calculations. The values found for the HOMO coefficient and charge density were 0.009 and −0.259, respectively, which are small enough to promote the nucleophilic attack that leads to a heterocycle. The structures of all the compounds were confirmed by ^1^H and ^13^C NMR, LC–MS, and elemental analysis. The structures of compounds **6**, **9c**, and **12g** were additionally confirmed via crystallographic data (Figure 1).

## Conclusion

The synthesis of pyrido[1,2-*a*]pyrimidinone, thiazolo[3,2-*a*]pyrimidinone, and pyrimidobenzimidazole polyazaheterocycles through the cyclocondensation reactions between β-enaminodiketone and aromatic amidines was highly regioselective. Results obtained from DFT-B3LYP theoretical calculations were in agreement with the experimental data. Using data from HOMO and LUMO coefficients, it was possible to conclude that the reaction is controlled by frontier molecular orbital component, which influenced the choice of reaction mechanism that was proposed.

The synthesis of pyrazinone and quinoxalinone heterocycles through cyclocondensation reactions between α-ketoester polyazaheterocycles and ethylenediamine and phenylenediamine derivatives was also successful. Isomeric product mixtures were formed when non-symmetric diamines were used as dinucleophiles. Theoretical calculations provided data regarding the stability of these isomeric mixtures.

However, the synthesis of imidazolone heterocycles was not achieved. Nevertheless, the N-acylated product was formed from the condensation reaction between the α-ketoester fragment in polyazaheterocycles and amidines. The experimental results and theoretical calculations of HOMO/LUMO coefficients, together with charge density and energetic stability of the intermediates, indicate that reactions between α-ketoesters and dinucleophiles are thermodynamically controlled. The proposed reaction mechanism, which is based on DFT-B3LYP data, demonstrates that an intermediate amide is expected, rather than an imine amide.

## Supporting Information

File 1 Additional information, characterization methods, experimental, analytical data, and copies of NMR spectra.
