# Supplementary material for: Regiochemistry of cyclocondensation reactions in the synthesis of polyazaheterocycles
Source: Beilstein J Org Chem. 2017 Feb 10;13:257–66. doi: 10.3762/bjoc.13.29 (PMC5331339; doi:10.3762/bjoc.13.29)
Supplement: File 1 — Additional information, characterization methods, experimental, analytical data, and copies of NMR spectra. [file Beilstein_J_Org_Chem-13-257-s001.pdf]

Supporting Information

for

Regiochemistry of cyclocondensation reactions in the synthesis of polyazaheterocycles

Patrick T. Campos[1], Leticia V. Rodrigues[2], Andrei L. Belladona[2], Caroline R. Bender[2], Juliana, S. Bitencurt[2], Fernanda A. Rosa[3], Davi F. Back[4], Helio G. Bonacorso[2], Nilo Zanatta[2], Clarissa P. Frizzo[2], and Marcos A. P. Martins[2§]*

Address: [1]IFSul Campus de Pelotas, Instituto Federal de Educação, Ciência e Tecnologia Sul-Rio-Grandense, 96.015-360, Pelotas, RS, Brazil, [2]Núcleo de Química de Heterociclos (NUQUIMHE), Departamento de Química, Centro de Ciências Naturais e Exatas, Universidade Federal de Santa Maria, 97.105-900, Santa Maria, RS, Brazil, [3]Departamento de Química, Centro de Ciências Exatas, Universidade Estadual de Maringá, 87020-900,Maringa, PR, Brazil and [4]Departamento de Química, Universidade Federal de Santa Maria, 97105-900, Santa Maria, RS, Brazil

[§]Tel.: +55 55 3220-8756; Fax: +55 55 3220-8031;
Email: Marcos A. P. Martins - mmartins@base.ufsm.br
*Corresponding author

Additional information, characterization methods, experimental, analytical data, and copies of NMR spectra

1. Additional Information

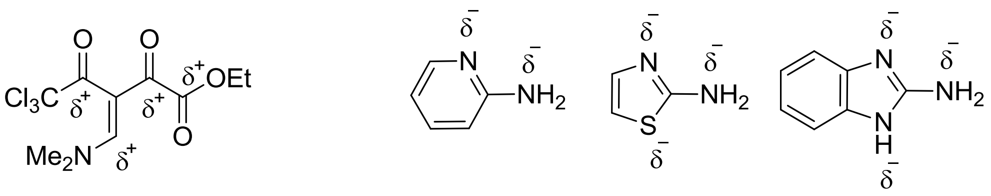

1 2 3 4

Figure S1: (A) Representation of β-enaminodiketone **1** electrophilic centers and (B) nucleophilic centers in structures for **2–4**.

Table S1: HOMO coefficients and charges for selected atoms in compounds **8a–f** obtained by DFT-B3LYP calculations.

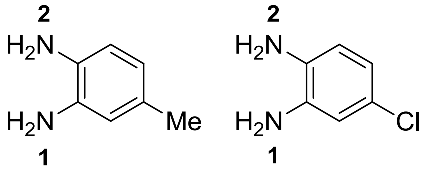

Compound	**8e**		**8f**	
HOMO (a.u.)	-0.194		-0.204	
Átoms	*N1*	*N2*	*N1*	*N2*
$Coef._{HOMO}$	0.147	0.157	0.146	0.164
Charge density	-0.313	-0.317	-0.307	-0.314

2. Characterization methods

The ^{1}H and ^{13}C spectra were recorded on a Bruker DPX 400 spectrometer (^{1}H NMR at 400 MHz and ^{13}C NMR at 100.6 MHz), digital resolution of ± 0.01 ppm, with 0.5 M solutions in $CDCl_3$ or DMSO as solvent. The ESI mass spectra was performed on an Agilent 6460 Triple Quadrupole, connected to a 1200

series LC. The mass spectrometer was operated in the positive jet stream electrospray ionization (ESI) mode. Elemental analyses were performed on a Perkin Elmer CHN elemental analyzer. The diffraction measurements were carried out by graphite monochromatized MoKα radiation with λ = 0.71073 Å on a Bruker SMART CCD diffractometer [1]. The structures were solved with direct methods using the SHELXS-97 program and refined on F2 by full-matrix least-squares by SHELXL-97 [2]. The absorption correction was performed by Gaussian methods [3]. The anisotropic displacement parameters for non-hydrogen atoms were applied. The hydrogen atoms were placed at calculated positions with 0.96 Å (methyl CH_3), 0.97 Å (methylene CH_2), 0.98 Å (methine CH), 0.93 Å (aromatic CH) and 0.86 Å (NH) using a riding model. The hydrogen isotropic thermal parameters were kept equal to $U_{iso}(H) = xU_{eq}$ (carrier C atom), with x = 1.5 for methyl groups and x = 1.2 otherwise. The valence angles C–C–H and H–C–H of methyl groups was set to 109.5° and H atoms were allowed to rotate around the C–C bond. Molecular graphics were prepared using ORTEP3 for Windows [4,5].

The assignment of signals in the ^{1}H NMR spectra for the isomers of compounds **10** was difficult due to the similarity of the compounds but could be accomplished with the aid of two-dimensional NMR techniques as COSY and HETCOR. Firstly, for **10e** the singlet at 7.6 ppm is attributed to H37 and the same proton in compound **10e’** resonates as a multiplet in the range of 7.7–7.6 ppm. Protons H34 and H35 could be assigned through their coupling by COSY correlations. In case of **10e**' the protons H34 and the H35 appear as a multiplet in the range of 7.2–7.1 ppm and couple with H36 and H37. The carbon atoms

were attributed using the HETCOR experiment. The same sequence of steps was used for the assignment of the signals of the others regioisomers.

2.1. Theoretical calculations

All theoretical calculations were performed with the Gaussian09 program package [6]. With the aim at obtaining the most stable regioisomer of the compounds, a 360° scan of the dihedral angle O=C–C=O change was performed in steps of 10° at the HF/6-31G level. A 360° scan of the dihedral angle N–C–C–N was also performed for the precursors 1,2-diaminepropane in steps of 10° at the HF/6-31G level. The more stable regioisomers were reoptimized at the B3LYP/cc-pVTZ level. All other geometries of the compounds were fully optimized at the B3LYP/cc-pVTZ level of theory and verified as minima in the potential energy by calculating the Hessian matrices by harmonic frequency calculations. HOMO and LUMO energy values and coefficients were obtained from geometries optimized at the B3LYP/cc-pVTZ level of theory.

2.2. X-ray crystallography.

Atomic coordinates, bond lengths, bond angles, and thermal parameters for **6**, **9c** and **12g** have been deposited at the Cambridge Crystallographic Data Centre (CCDC). The data can be obtained free of charge via www.ccdc.cam.uk/conts/retrieving.html (or from the CCDC, 12 Union Road, Cambridge CB21EZ; fax: +44 1223 335 033; or deposit@ccdc.cam.ac.uk). Any request to the CCDC for data should quote the full literature citation and CCDC reference numbers.

Crystal data for 6. Obtained from MeCN: $C_{10}H_8N_2O_4S$, *M* = 252.24, space group P_{212121} (orthorhombic), *a* = 8.2539(2), *b* = 9.9582(2), *c* = 13.8149(4) Å, *V* = 1135.50(5) $Å^3$, Z = 4, D_c = 1.476 g·cm^3, μ(MoKα) = 0.289 mm^{-1}, 293(2) K, 10870 reflections collected, 2513 unique (R_{int} = 0.0434), 2513 reflections with I > 2σ(I), 155 parameters, R_1 = 0,1077, wR_2 (all data) = 0.1731.

CCDC reference number 846497.

Crystal Data for 9c. Obtained from MeCN/H_2O 9:1: $C_{16}H_{20}N_4O_4$, *M* = 332.36, space group P_{-1} (Triclinic), *a* = 8.6972(9), *b* = 9.4632(9), *c* = 10.6765(10) Å, α = 68.046(6)°, β = 77.045(7)°, γ = 82.175(7)°, *V* = 792.91(13) $Å^3$, Z = 2, D_c = 1.392 g·cm^3, μ(MoKα) = 0.102 mm^{-1}, 293(2) K, 12857 reflections collected, 3019 unique (R_{int} = 0.0567), 3019reflections with I > 2σ(I), 234 parameters, R_1 = 0.1107, wR_2 (all data) = 0.1753.

CCDC reference number 846499.

Crystal Data for 12g. Obtained from MeCN: $C_{12}H_{10}N_4O_3$, *M* = 258.24, space group P_{-1} (Triclinic), *a* = 7.3807(4), *b* = 8.7049(6), *c* = 10.2452(6) Å, α = 99.258(4)°, β = 110.800(4)°, γ = 105.762(4)°, *V* = 567.45(6) $Å^3$, Z = 2, D_c = 1.511 g·cm^3, μ(MoKα) = 0.113 mm^{-1}, 293(2) K, 9627 reflections collected, 2524 unique (R_{int} = 0.0279), 2524 reflections with I > 2σ(I), 172 parameters, R_1 = 0.0907, wR_2 (all data) = 0.1548.

CCDC reference number 846501.

Table S2: Data collection and structure refinement for structure of compound **6**, **9c** and **12g**.

Compound	**6**	**9c**	**12g**
CCDC Number	846497	846499	846498
Empirical formula	$C_{10}H_8N_2O_4S$	$C_{16}H_{20}N_4O_4$	$C_{12}H_{10}N_2O_4$
Formula weight	252.24	332.36	246.22
Temperature (K)	293(2)	293(2)	293(2)
Wavelenght (Å)	0.71073	0.71073	0.71073
Crystal system	Ortorrhombic	Triclinic	Triclinic
Space group	P_{212121}	P_{-1}	P_{-1}
a (Å)	8.2539(2)	8.6972(9)	6.6427(8)
b (Å)	9.9582(2)	9.4632(9)	6.8427(8)
c (Å)	13.8149(4)	10.6765(10)	13.4771(14)
α (deg)	90	68.046(6)	98.711(7)
β (deg)	90	77.045(7)	102.987(7)
γ (deg)	90	82.175(7)	97.767(8)
Volume ($Å^3$)	1135.50(5)	792.91(13)	580.86(11)
Z/density (calcd)(Mg/m^3)	4	2	2
Absorption coefficient (mm^{-1})	0.289	1.392	0.108
F(000) (e)	520	352	256
Crystal size (mm^3)	0.336 x 0.277 x 0.154	0.257 x 0.149 x 0.126	0.564 x 0.107 x 0.08
θ range fo data collection (deg)	2.87 to 27.11	2.32 to 25.70	3.06 to 26.46
Reflections collected/unique	10870 /2513 [R(int) = 0.0434]	12857 / 3019 [R(int) = 0.0567]	9408 / 2382 [R(int) = 0.0338]
Completeness to θ (%)	(27.11) 99.9 %	(27.16) 98.9%	(27.29) 98.9%
Absorption correction	Gaussian	Gaussian	Gaussian
Max, min transmission	0.9898, 0.9102	0.99991, 0.90390	0.9855, 0.9303
Refinement method	Full-matrix least-squares on F^2	Full-matrix least-squares on F^2	Full-matrix least-squares on F^2
Data/restraints/param.	2513 / 0 / 155	3019 / 0 / 234	2382 / 0 / 164
Goodness-of-fit on F^2	0.996	1.033	1.022
Final R indices [$I \geq 2\sigma (I)$][a]	R_1 = 0.0583. wR_2 = 0.1380	R_1 = 0.0567. wR_2 = 0.1436	R_1 = 0.0489. wR_2 = 0.1253
R_1 (all data)[a]	R_1 = 0.1077. wR_2 = 0.1731	R_1 = 0.1107. wR_2 = 0.1753	R_1 = 0.0993. wR_2 = 0.1555

3. Experimental section

3.1. General procedure for the preparation of compounds 5–7

A mixture of the amidine **2–4** (5 mmol) and β-enaminodiketone **1** (5 mmol) in acetonitrile (15 mL) was kept under stirring and reflux for 0.5 h. Subsequently, the acetonitrile was evaporated under reduced pressure, and the resulting solid was purified by column chromatography, using silica gel as stationary phase and a mixture of ethyl acetate and hexane (3:1) as eluent for compounds **5** and **6**. For compound **7**, acetonitrile was used as the eluent.

Ethyl 2-oxo-2-(4-oxo-4*H*-pyrido[1,2-*a*]pyrimidin-3-yl) acetate (5)

Yield 80%, m.p. 159.31 °C.^{1}H NMR (400 MHz, $CDCl_3$): δ 1.42 (t, 3H, H35), 4.46 (q, 2H, H34), 7.47 (td, 1H, $^{1}J_{HH}$ = 7.1; $^{2}J_{HH}$ = 1.5; H8), 7.89 (ddd, 1H, $^{1}J_{HH}$ = 8.8; $^{2}J_{HH}$ = 1.5; $^{3}J_{HH}$ = 0.7; H6), 8.11 (ddd, 1H, $^{1}J_{HH}$ = 8.8; $^{2}J_{HH}$ = 6.8; $^{3}J_{HH}$ = 1.7; H7), 9.01 (s, 1H, H2), 9.26 (ddd, 1H, $^{1}J_{HH}$ = 7.1; $^{2}J_{HH}$ = 1.5; $^{3}J_{HH}$ = 0.7; H9). ^{13}C NMR (100 MHz, $CDCl_3$): δ 14,0 (C35), 62.2 (C34), 108.5 (C3), 118.2 (C8), 127.3 (C6), 128.7 (C9), 141,0 (C7), 153.9 (C4), 156.2 (C9a), 158.5 (C2), 165.0 (C32), 184.6 (C31). MS (ESI) *m/z*: [$(M+H)^+$, 247], [$(M+Na)^+$, 269]. Anal. Calcd for $C_{12}H_{10}N_2O_4$: C, 58.54; H, 4.09; N, 11.38. Found: C, 58.31; H, 4.23; N, 11.65.

Ethyl 2-oxo-2-(5-oxo-5*H*-thiazolo[3,2-*a*]pyrimidin-6-yl) acetate (6)

Yield 86%, m.p. 130.42 °C. ^{1}H NMR (400 MHz, $CDCl_3$): δ 1.41 (t, 3H, CH_3), 4.44 (q, 2H, CH_2), 7.31 (d, 1H, $^{1}J_{HH}$ = 4.6, H7), 8.18 (d, 1H, $^{1}J_{HH}$ = 4.9, H6), 8.80 (s, 1H, H2). ^{13}C NMR (100 MHz, $CDCl_3$): δ 13.9 (C35), 62.3 (C34), 110.9 (C3), 114.7 (C7), 123.3 (C6), 156.4 (C4), 157.9 (C2), 164.8 (C32), 168.4 (C8a), 184.5

(C31). MS (ESI) *m/z*: [(M+Na)$^+$, 275]. Anal. Calcd for $C_{10}H_8N_2O_4S$: C, 47.61; H, 3.20; N, 11.11. Found: C, 47.34; H, 3.17; N, 11.05.

Ethyl 2-oxo-2-(2-oxo-2*H*,6*H*-pyrimido[1,2-*a*]benzoimidazole-3-yl) acetate (7)
Yield 56%, m.p. 262.96 °C. ^{1}H NMR (400 MHz, DMSO-d_6): δ 1.34 (t, 3H, H35), 4.35 (q, 2H, H34), 7.43 (t, 1H, $^1J_{HH}$ = 7.8; H7), 7.54 (t, 1H, $^1J_{HH}$ = 7.8; H8), 7.63 (d, 1H, $^1J_{HH}$ = 8.1; H9), 8.43 (d, 1H, $^1J_{HH}$ = 8.1; H6), 8.70 (s, 1H, H2). ^{13}C NMR (100 MHz, DMSO-d_6): δ 13.6 (C35), 61.0 (C34), 106.9 (C3), 113.2 (C9), 115.7 (C6), 123.2 (C7), 126.4 (C8), 126.5 (C5a), 132.3 (C9a), 151.6 (C4), 158.2 (C10a), 158.8 (C2), 165.3 (C32), 183.6 (C31). MS (ESI) *m/z*: [(M+H)$^+$, 286], [(M+Na)$^+$, 308]. Anal. Calcd for $C_{14}H_{11}N_3O_4$: C, 58.95; H, 3.89; N, 14.73. Found: C, 58.75; H, 3.87; N, 14.49.

2. General procedure for the preparation of compounds 9a–f, 9b', 9e', 9f', 10c–f, 10e', 10f', 11a–e and 11b'

A mixture of diamine **8a–f** (0.5 mmol) and α-ketoester **5–7** (0.5 mmol) in acetonitrile (2 mL) was kept under agitation and reflux for 2–24 h. The acetonitrile was then evaporated under reduced pressure, and the resulting solid was purified by washing with ethyl acetate (3 × 1 mL) for compounds **9a–f, 9b', 9e', 9f', 10c–f, 10e', 10f', 11a–e, 11b'** and **11a**, and by simple filtration for compounds **11b–e** and **11b'**.

3-(3-Oxo-3,4,5,6-tetrahydropyrazin-2-yl)-4*H*-pyrido[1,2-*a*]pyrimidin-4-one (9a)

Yield 83%, - Decomposition Temperature. 251.07°. ^{1}H NMR (400 MHz, DMSO-*d*$_6$): δ 3,39-3,35 (m, 2H, H34), 3.78 (t, 2H, H35), 7.44 (td, 1H, $^{1}J_{HH}$ = 7.1; $^{2}J_{HH}$ = 1.5; H8), 7.75 (ddd, 1H, $^{1}J_{HH}$ = 8.8; $^{2}J_{HH}$ = 1.5; $^{3}J_{HH}$ = 0.7; H6), 8.03 (ddd, 1H, $^{1}J_{HH}$ = 8.8; $^{2}J_{HH}$ = 6.6; $^{3}J_{HH}$ = 1.5; H7), 8.26 (bs, 1H, NH), 8.39 (s, 1H, H2), 9.00 (ddd, 1H, $^{1}J_{HH}$ = 7.1; $^{2}J_{HH}$ = 1.5; $^{3}J_{HH}$ = 0.7; H9). ^{13}C NMR (100 MHz, DMSO-*d*$_6$): δ 38.1 (C34), 48.3 (C35), 113.6 (C3), 116.9 (C8), 125.8 (C6), 127.0 (C9), 137.8 (C7), 151.4 (C4), 153.2 (C2), 155.2 (C9a), 155.4 (C32), 160.9 (C31). MS (ESI) *m/z*: [(M+H)$^+$, 243], [(M+Na)$^+$, 265]. Anal. Calcd for $C_{12}H_{10}N_4O_2$: C, 59.50; H, 4.16; N, 23.13. Found: C, 59.25; H, 4.11; N, 23.09.

3-(6-Methyl-3-oxo-3,4,5,6-tetrahydropyrazin-2-yl)-4*H*-pyrido[1,2-*a*]pyrimidin-4-one (9b)

Yield 68% with 27% of isomer **9b**, m.p. 134.16 or 154.17°C. ^{1}H NMR (400 MHz, DMSO-*d*$_6$): δ 3.12 (ddd, 1H, $^{1}J_{HH}$ = 13; $^{2}J_{HH}$ = 10.3; $^{3}J_{HH}$ = 2; H34’), 3.44-3.37 (m, H34’’), 3.86 -3.81 (m, 1H, H35), 7.46 (td, 1H, $^{1}J_{HH}$ = 7.1; $^{2}J_{HH}$ = 1.5; H8), 7.77 (d, 1H, $^{1}J_{HH}$ = 8.8; H6), 8.05 (ddd, 1H, $^{1}J_{HH}$ = 8.8; $^{2}J_{HH}$ = 6.8; $^{3}J_{HH}$ = 1.5; H7), 8.40 (s, 1H, H2), 8.46 (bs, 1H, NH33), 9.0 (d, 1H, $^{1}J_{HH}$ = 7.3; H9). ^{13}C NMR (100 MHz, DMSO-*d*$_6$): δ 18.5 (C351), 43.0 (C34), 54.9 (C35), 113.7 (C3), 117.1 (C8), 126.0 (C6), 127.1 (C9), 138.1 (C7), 151.5 (C4), 153.3 (C2), 155.4 (C9), 155.8 (C32), 160.8 (C31). MS (ESI) *m/z*: [(M+H)$^+$, 257], [(M+Na)$^+$, 279]. Anal. Calcd for $C_{13}H_{12}N_4O_2$: C, 60.93; H, 4.72; N, 21.86. Found: C, 59.11; H, 4.67; N, 21.57.

3-(5-Methyl-3-oxo-3,4,5,6-tetrahydropyrazin-2-yl)-4*H*-pyrido[1,2-*a*]pyrimidin-4-one (9b')

Yield 68% with 73% of isomer **9b'**, m.p. 134.16 or 154.17°C. ^{1}H NMR (400 MHz, DMSO-d_6): δ 3.44-3.37 (m, H35''), 3.71 (m, 1H, H34), 3.95 (dd, 1H, $^{1}J_{HH}$ = 16.1; $^{2}J_{HH}$ = 4.1; H35'), 7.46 (td, 1H, $^{1}J_{HH}$ = 7.1; $^{2}J_{HH}$ = 1.5; H8), 7.77 (d, 1H, $^{1}J_{HH}$ = 8.8; H6), 8.05 (ddd, 1H, $^{1}J_{HH}$ = 8.8; $^{2}J_{HH}$ = 6.8; $^{3}J_{HH}$ = 1.5; H7), 8.39 (s, 1H, H2), 8.46 (bs, 1H, NH33), 9.0 (d, 1H, $^{1}J_{HH}$ = 7.3; H9). ^{13}C NMR (100 MHz, DMSO-d_6): δ 17.9 (C341), 44.0 (C34), 53.2 (C35), 113.7 (C3), 117.1 (C8), 126.0 (C6), 127.1 (C9), 138.1 (C7), 151.5 (C4), 153.3 (C2), 155.4 (C9), 155.8 (C32), 160.8 (C31). MS (ESI) *m/z*: [(M+H)$^+$, 257], [(M+Na)$^+$, 279]. Anal. Calcd for $C_{13}H_{12}N_4O_2$: C, 60.93; H, 4.72; N, 21.86. Found: C, 59.11; H, 4.67; N, 21.57.

3-(3-Oxo-3,4,4a,5,6,7,8,8a-octahydroquinoxalin-2-yl)-4*H*-pyrido[1,2-*a*]pyrimidin-4-one (9c)

Yield 72%, m.p. 214.04°C. ^{1}H NMR (400 MHz, DMSO-d_6): δ 1.42-1.30 (m, 4H, H34''+H35''+H36''+H37''), 1.71 (d, 1H, $^{1}J_{HH}$ = 9.8; H35'), 1.78 (d, 1H, $^{1}J_{HH}$ = 9.3; H36'), 1.97 (d, 1H, $^{1}J_{HH}$ = 10.3; H34'), 2.25 (d, 1H, $^{1}J_{HH}$ = 9.8; H37'), 3.16-1.14 (m, 2H, H33a + H37a), 7.44 (td, 1H, $^{1}J_{HH}$ = 7.1; $^{2}J_{HH}$ = 1.2; H8), 7.75 (d, 1H, $^{1}J_{HH}$ = 8.8; H6), 8.03 (ddd, 1H, $^{1}J_{HH}$ = 8.8; $^{2}J_{HH}$ = 7.1; $^{3}J_{HH}$ = 1.5; H7), 8.33 (bs, 1H, NH), 8.39 (s, 1H, H2), 9.0 (d, 1H, $^{1}J_{HH}$ = 7.1; H9). ^{13}C NMR (100 MHz, DMSO-d_6): δ 23.1 (C35), 24.6 (C36), 30.1 (C34), 31.3 (C37), 53.8 (C33a), 62.9 (C37a), 113.4 (C3), 117.0 (C8), 125.9 (C6), 127.0 (C9), 138.0 (C7), 151.5 (C4), 153.3 (C2), 155.3 (C9a), 156.3 (C32), 160.3 (C31). MS (ESI) *m/z*: [(M+H)$^+$, 297], [(M+Na)$^+$, 319]. Anal. Calcd for $C_{16}H_{16}N_4O_2$: C, 64.85; H, 5.44; N, 18.91. Found: C, 64,61; H, 5,43; N, 18.62.

3-(3-Oxo-3,4-dihydroquinoxalin-2-yl)-4*H*-pyrido[1,2-*a*]pyrimidin-4-one (9d)

Yield 87%, m.p. 304.93°C. ^{1}H NMR (400 MHz, DMSO-d_6): δ 7.32 (ddd, 1H, $^1J_{HH}$ = 8.1; $^2J_{HH}$ = 7.3; $^3J_{HH}$ = 1.5; H36), 7.37 (dd, 1H, $^1J_{HH}$ = 8.3; $^2J_{HH}$ = 1.5; H34), 7.48 (td, 1H, $^1J_{HH}$ = 7.1; $^2J_{HH}$ = 1.5; H8), 7.55 (ddd, 1H, $^1J_{HH}$ = 8.1; $^2J_{HH}$ = 7.3; $^3J_{HH}$ = 1.5; H35), 7.82-7.79 (m, 2H, H6 e H37), 8.07 (ddd, 1H, $^1J_{HH}$ = 8.8; $^2J_{HH}$ = 6.8; $^3J_{HH}$ = 1.5; H7), 12.39 (bs, 1H, NH), 8.64 (s, 1H, H2), 9.1 (ddd, 1H, $^1J_{HH}$ = 7.1; $^2J_{HH}$ = 1.5; $^3J_{HH}$ = 0.7; H9). ^{13}C NMR (100 MHz, DMSO-d_6): δ 112.4 (C3), 115.1 (C34), 116.9 (C8), 123.0 (C36), 125.9 (C6), 127.3 (C9), 128.4 (C37), 130.1 (C35), 131.8 (C33a), 132.1 (C37a), 138.0 (C7), 151.4 (C4), 153.7, (C32) 154.2 (C9a), 154.4 (C2), 154.8 (C31). MS (ESI) *m/z*: [(M+H)$^+$, 291], [(M+Na)$^+$, 313]. Anal. Calcd for $C_{16}H_{10}N_4O_2$: C, 66.20; H, 3.47; N, 19.30. Found: C, 65.92; H, 3.33; N, 19.31.

3-(7-Methyl-3-oxo-3,4-dihydroquinoxalin-2-yl)-4*H*-pyrido[1,2-*a*]pyrimidin-4-one (9e)

Yield 65% with 44% of isomer **9e**, m.p. 211.80 or 216.55°C. ^{1}H NMR (400 MHz, DMSO-d_6): δ 2.46 (s, 3H, H361), 7.26 (d, 1H, $^1J_{HH}$ = 8.1; H35), 7.38 (d, 1H, $^1J_{HH}$ = 8.1; H34), 7.47 (t, 1H, $^1J_{HH}$ = 7.1; H8), 7.01 (s, 1H, H37), 7.79 (d, 1H, $^1J_{HH}$ = 8.8; H6), 8.06 (t, 1H, $^1J_{HH}$ = 6.8; H7), 8.61 (s, 1H, H2), 9.09 (d, 1H, $^1J_{HH}$ = 7.1; H9), 12.31 (bs, 1H, H33). ^{13}C NMR (100 MHz, DMSO-d_6): δ 21.1 (C361), 114.8 (C35), 116.9 (C8), 125.9 (C6), 127.3 (C9), 128.2 (C37), 130.2 (C33a), 131.3 (C34), 132.0 (C37a), 137.9 (C7), 140.5 (C36), 151.8 (C4), 152.9 (C32), 154.0 (C9a), 154.3 (C2), 154.8 (C31). MS (ESI) *m/z*: [(M+H)$^+$, 305], [(M+Na)$^+$, 327]. Anal. Calcd for $C_{17}H_{12}N_4O_2$: C, 67.10; H, 3.97; N, 18.41. Found: C, 67.02; H, 3.88; N, 18.18.

3-(6-Methyl-3-oxo-3,4-dihydroquinoxalin-2-yl)-4*H*-pyrido[1,2-*a*]pyrimidin-4-one (9e')

Yield 65% with 56% of isomer **9e'**, m.p. 211.80 or 216.55°C. ^{1}H NMR (400 MHz, DMSO-d_6): δ 2.40 (s, 3H, H351), 7.15-7.14 (m, 2H, H34+H36), 7.47 (t, 1H, $^{1}J_{HH}$ = 7.1; H8), 7.68 (d, 1H, $^{1}J_{HH}$ = 8.6), 7.79 (d, 1H, $^{1}J_{HH}$ = 8.8; H6), 8.06 (t, 1H, $^{1}J_{HH}$ = 6.8; H7), 8.61 (s, 1H, H2), 9.09 (d, 1H, $^{1}J_{HH}$ = 7.1; H9), 12.31 (bs, 1H, H33). ^{13}C NMR (100 MHz, DMSO-d_6): δ 20.1 (C351), 114.7 (C36), 116.9 (C8), 124.4 (C34), 125.9 (C6), 127.3 (C9), 128.0 (C37), 129.8 (C33a), 131.8 (C37a), 132.4 (C35), 137.9 (C7), 151.3 (C4), 153.6 (C32), 153.9 (C9a), 154.3 (C2), 154.8 (C31). MS (ESI) *m/z*: [(M+H)$^+$, 305], [(M+Na)$^+$, 327]. Anal. Calcd for $C_{17}H_{12}N_4O_2$: C, 67.10; H, 3.97; N, 18.41. Found: C, 67.02; H, 3.88; N, 18.18.

3-(7-Chloro-3-oxo-3,4-dihydroquinoxalin-2-yl)-4*H*-pyrido[1,2-*a*]pyrimidin-4-one (9f)

Yield 66% with 55% of isomer **9f**, m.p. 259.47 or 281.99°C. ^{1}H NMR (400 MHz, DMSO-d_6): δ 7.38 (d, 1H, $^{1}J_{HH}$ = 8.8; H35), 7.48 (td, 1H, $^{1}J_{HH}$ = 7.1; $^{2}J_{HH}$ = 1.2; H8), 7.57 (dd, 1H, $^{1}J_{HH}$ = 8.8; $^{2}J_{HH}$ = 2.4; H34), 7.8 (d, 1H, $^{1}J_{HH}$ = 8.6; H6), 7.83 (d, 1H, $^{1}J_{HH}$ = 2.4; H37), 8.08 (ddd, 1H, $^{1}J_{HH}$ = 8.8; $^{2}J_{HH}$ = 6.6; $^{3}J_{HH}$ = 1.5; H7), 8.65 (s, 1H, H2), 9.09 (d, 1H, $^{1}J_{HH}$ = 7.1; H9), 12.47 (bs, 1H, NH33). ^{13}C NMR (100 MHz, DMSO-d_6): δ 111.9 (C3), 116.7 (C35), 117.0 (C8), 125.9 (C6), 127.3 (C9), 127.3 (C37), 130.0 (C34), 131.0 (C33a), 133.1 (C37a), 134.2 (C36), 138.2 (C7), 151.5 (C4), 153.4 (C32), 154.5 (C9a), 154.6 (C2), 155.5 (C31). MS (ESI) *m/z*: [(M+H)$^+$, 325], [(M+Na)$^+$, 347]. Anal. Calcd for $C_{16}H_9ClN_4O_2$: C, 59.18; H, 2.79; N, 17.25. Found: C, 58.98; H, 2.85; N, 16.97.

3-(6-Chloro-3-oxo-3,4-dihydroquinoxalin-2-yl)-4*H*-pyrido[1,2-*a*]pyrimidin-4-one (9f')

Yield 66% with 45% of isomer **9f'**, m.p. 259.47 or 281.99°C. ^{1}H NMR (400 MHz, DMSO-d_6): δ 7.32 (dd, 1H, $^{1}J_{HH}$ = 8.6; $^{2}J_{HH}$ = 2.2; H36), 7.38 (d, 1H, $^{1}J_{HH}$ = 2.2; H34), 7.48 (td, 1H, $^{1}J_{HH}$ = 7.1; $^{2}J_{HH}$ = 1.2; H8), 7.8 (d, 2H, $^{1}J_{HH}$ = 8.6; H6+H37), 8.07 (ddd, 1H, $^{1}J_{HH}$ = 8.6; $^{2}J_{HH}$ = 6.8; $^{3}J_{HH}$ = 1.7; H7), 8.64 (s, 1H, H2), 9.09 (d, 1H, $^{1}J_{HH}$ = 7.1; H9), 12.47 (bs, 1H, NH33). ^{13}C NMR (100 MHz, DMSO-d_6): δ 111.9 (C3), 114.4 (C34), 117.0 (C8), 123.1 (C36), 125.9 (C6), 126.7 (C35), 127.3 (C9), 129.9 (C37), 130.6 (C33a), 132.4 (C37a), 138.2 (C7), 151.4 (C4), 154.4 (C32), 154.5 (C9a), 154.6 (C2), 155.5 (C31). MS (ESI) *m/z*: [(M+H)$^+$, 325], [(M+Na)$^+$, 347]. Anal. Calcd for $C_{16}H_9ClN_4O_2$: C, 59.18; H, 2.79; N, 17.25. Found: C, 58.98; H, 2.85; N, 16.97.

6-(3-Oxo-3,4,4a,5,6,7,8,8a-octahydroquinoxalin-2-yl)-5*H*-thiazolo[3,2-*a*]pyrimidin-5-one (10c)

Yield 77%. ^{1}H NMR (400 MHz, DMSO-d_6): δ 1.40-1.29 (m, 4H, H34'+H35'+H36'+H37'), 1.70 (d, 1H, $^{1}J_{HH}$ = 10; H35'), 1.78 (d, 1H, $^{1}J_{HH}$ = 10; H36'), 1.95 (d, 1H, $^{1}J_{HH}$ = 8.8; H34'), 2.24 (d, 1H, $^{1}J_{HH}$ = 9.0; H37'), 3.14-3.12 (m, 2H, H33a+H37a), 7.63 (d, 1H, $^{1}J_{HH}$ = 4.9), 8.08 (d, 1H, $^{1}J_{HH}$ = 4.6; H6), 8.12 (s, 1H, H2), 8.34 (bs, 1H, NH). ^{13}C NMR (100 MHz, DMSO-d_6): δ 23.1 (C35), 24.6 (C36), 30.0 (C34), 31.2 (C37), 53.7 (C33a), 62.8 (C37a), 114.4 (C7), 115.3 (C3), 121.8 (C6), 151.8 (C2), 155.9 (C4), 156.1 (C32), 159.9 (C8a), 163.9 (C31). MS (ESI) *m/z*: [(M+H)$^+$, 303], [(M+Na)$^+$, 325]**.** Anal. Calcd for $C_{14}H_{14}N_4O_2S$: C, 55.61; H, 4.67; N, 18.53.

6-(3-Oxo-3,4-dihydroquinoxalin-2-yl)-5*H*-thiazolo[3,2-*a*]pyrimidin-5-one (10d)

Yield 93%, m.p. 348.02°C. ^{1}H NMR (400 MHz, DMSO-d_6): δ 7.32 (t, 1H, $^{1}J_{HH}$ = 7.8, H36), 7.36 (d, 1H, $^{1}J_{HH}$ = 8.1, H34), 7.55 (t, 1H, $^{1}J_{HH}$ = 7.8, H35), 7.66 (d, 1H, $^{1}J_{HH}$ = 4.6, H7), 7.79 (d, 1H, $^{1}J_{HH}$ = 7.8, H37), 8.15 (d, 1H, $^{1}J_{HH}$ = 4.9, H6), 8.36 (s, 1H, H2). ^{13}C NMR (100 MHz, DMSO-d_6): δ 114.4 (C3), 114.5 (C7), 115.3 (C34), 122.2 (C6), 123.3 (C36), 128.6 (C37), 130.5 (C34), 131.9 (C33a), 132.2 (C37a), 153.0 (C2), 153.7 (C4), 153.8 (C32), 155.7 (C8a), 164.1 (C31). MS (ESI) *m/z*: [(M+H)$^+$, 297], [(M+Na)$^+$, 319]. Anal. Calcd for $C_{14}H_8N_4O_2S$: C, 56.75; H, 2.72; N, 18.91. Found: C, 56.48; H, 2.52; N, 18.80.

6-(7-Methyl-3-oxo-3,4-dihydroquinoxalin-2-yl)-5*H*-thiazolo[3,2-*a*]pyrimidin-5-one (10e)

Yield 70% with 42% of isomer **10e**, m.p. 302.90 or 315.75°C. ^{1}H NMR (400 MHz, DMSO-d_6): δ 2.43 (s, 3H, H361), 7.25 (d, 1H, $^{1}J_{HH}$ = 8.3; H35), 7.38 (d, 1H, $^{1}J_{HH}$ = 8.8; H34), 7.59 (s, 1H, H37), 7.68-7.65 (m, 1H, H7), 8.15 (d, 1H, $^{1}J_{HH}$ = 4.6; H6), 8.35 (s, 1H, H2), 12.3 (bs, 1H, H33). ^{13}C NMR (100 MHz, DMSO-d_6): δ 21.1 (C361), 114.2 (C7), 114.4 (C4), 114.7 (C35), 122.1 (C6), 128.2 (C37), 129.8 (C33a), 131.4 (C34), 132.0 (C37a), 140.6 (C36), 152.8 (C2), 153.6 (C4), 153.6 (C32), 155.5 (C8a), 163.8 (C31). MS (ESI) *m/z*: [(M+H)$^+$, 311], [(M+Na)$^+$, 333], [(M+K)$^+$, 349]. Anal. Calcd for $C_{15}H_{10}N_4O_2S$: C, 58.05; H, 3.25; N, 18.05. Found: C, 58.19; H, 3.21; N, 18.04.

6-(6-Methyl-3-oxo-3,4-dihydroquinoxalin-2-yl)-5*H*-thiazolo[3,2-*a*]pyrimidin-5-one (10e')

Yield 70% with 58% of isomer **17**, m.p. 302.90 or 315.75°C. ^{1}H NMR (400 MHz, DMSO-d_6): δ 2.39 (s, 3H, H351), 7.15-7.13 (m, 2H, H34+H36), 7.68-7.65 (m, 1H, H7+H37), 8.15 (d, 1H, $^1J_{HH}$ = 4.6; H6), 8.35 (s, 1H, H2), 12.3 (bs, 1H, H33). ^{13}C NMR (100 MHz, DMSO-d_6): δ 20.1 (C351), 114.2 (C7), 114.4 (C4), 114.8 (C36), 122.1 (C6), 124.4 (C34), 128.1 (C37), 130.1 (C37), 131.7 (C37a), 132.4 (C35), 152.4 (C4), 152.8 (C2), 153.8 (C32), 155.5 (C8a), 163.7 (C31).

MS (ESI) *m/z*: [(M+H)$^+$, 311], [(M+Na)$^+$, 333], [(M+K)$^+$, 349]. Anal. Calcd for $C_{15}H_{10}N_4O_2S$: C, 58.05; H, 3.25; N, 18.05. Found: C, 58.19; H, 3.21; N, 18.04.

6-(7-Chloro-3-oxo-3,4-dihydroquinoxalin-2-yl)-5*H*-thiazolo[3,2-*a*]pyrimidin-5-one (10f)

Yield 66% with 48% of isomer **10f**, m.p. 150.61 or 171.00°C. ^{1}H NMR (400 MHz, DMSO-d_6): δ 7.38-7.35 (m, 1H, H35), 7.58 (dd, 1H, $^1J_{HH}$ = 8.6; $^2J_{HH}$ = 2.2; H34), 7.68 (d, 1H, $^1J_{HH}$ = 4.6; H7), 7.82 (d, 1H, $^1J_{HH}$ = 2.2; H37), 8.16 (d, 1H, $^1J_{HH}$ = 4.9; H6), 8.39 (s, 1H, H2), 12.51 (bs, 1H, NH33). ^{13}C NMR (100 MHz, DMSO-d_6): δ 113.91 (C3), 114.53 (C7), 116.8 (C35), 122.2 (C6), 127.5 (C37), 130.2 (C34), 131.1 (C33a), 133.2 (C37a), 134.4 (C36), 153.2 (C2), 153.5 (C4), 154.1 (C32), 155.4 (C8a), 164.2 (C31). MS (ESI) *m/z*: [(M+H)$^+$, 331], [(M+Na)$^+$, 353]. Anal. Calcd for $C_{14}H_7ClN_4O_2S$: C, 50.84; H, 2.13; N, 16.94. Found: C, 50.61; H, 2.33; N, 17.03.

6-(6-Chloro-3-oxo-3,4-dihydroquinoxalin-2-yl)-5*H*-thiazolo[3,2-*a*]pyrimidin-5-one (10f')

Yield 66% with 52% of isomer **10f'**, m.p. 150.61 or 171.00°C. ^{1}H NMR (400 MHz, DMSO-d_6): δ 7.32 (dd, 1H, $^1J_{HH}$ = 8.6; $^2J_{HH}$ = 2.2; H36), 7.38-7.35 (m, 1H, H34), 7.68 (d, 1H, $^1J_{HH}$ = 4.6; H7), 7.79 (d, 1H, $^1J_{HH}$ = 8.6; H37), 8.15 (d, 1H, $^1J_{HH}$ = 4.6; H6), 8.38 (s, 1H, H2), 12.51 (bs, 1H, NH33). ^{13}C NMR (100 MHz, DMSO-d_6): δ 113.9 (C3), 114.5 (C34), 114.5 (C7), 122.2 (C6), 123.3 (C36), 126.8 (C35), 130.2 (C37), 130.6 (C33a), 132.3 (C37a), 153.2 (C2), 153.5 (C4), 155.2 (C32), 155.4 (C8a), 164.1 (C31). MS (ESI) *m/z*: [(M+H)$^+$, 331], [(M+Na)$^+$, 353]. Anal. Calcd for $C_{14}H_7ClN_4O_2S$: C, 50.84; H, 2.13; N, 16.94. Found: C, 50.61; H, 2.33; N, 17.03.

3-(3-Oxo-3,4,5,6-tetrahydropyrazin-2-yl)-2-oxo-2*H*,6*H*-pyrimido[1,2-*a*]benzoimidazole (11a)

Yield 78%, m.p. 251.22°C. ^{1}H NMR (400 MHz, DMSO-d_6): δ 3.46 (t, 2H, H35), 3.80 (t, 2H, H35), 7.31 (ddd, 1H, $^1J_{HH}$ = 8.1; $^2J_{HH}$ = 7.3; $^3J_{HH}$ = 1.2; H7), 7.42 (ddd, 1H, $^1J_{HH}$ = 8.1; $^2J_{HH}$ = 7.3; $^3J_{HH}$ = 1.2; H8), 7.64 (d, 1H, $^1J_{HH}$ = 8.1; H9), 8.36 (ddd, 1H, $^1J_{HH}$ = 8.1; $^2J_{HH}$ = 1.2; $^3J_{HH}$ = 0.7; H6), 9.06 (s, 1H, H2). ^{13}C NMR (100 MHz, DMSO-d_6): δ 37.6 (C34), 43.2 (C35), 114.9 (C9), 116.9 (C6), 121.7 (C7), 124.8 (C8), 128.0 (C5a), 140.7 (C9a), 152.4 (C4), 156.6 (C2), 157.2 (C32), 159.0 (C10a), 161.0 (C31). MS (ESI) *m/z*: [(M+H)$^+$, 282], [(M+Na)$^+$, 304]. Anal. Calcd for $C_{14}H_{11}N_5O_2$: C, 59.78; H, 3.94; N, 24.90. Found: C, 59.41; H, 3.89; N, 24.63.

3-(6-Methyl-3-oxo-3,4,5,6-tetrahydropyrazin-2-yl)-2-oxo-2*H*,6*H*-pyrimido[1,2-*a*]benzoimidazole (11b)

Yield 89% with 50% of isomer **11b**, m.p. 160.78 or 197.26°C. ^{1}H NMR (400 MHz, DMSO-d_6): δ 1.36 (d, 3H, $^{1}J_{HH}$ = 6.6; H351), 3.25 (dd, 1H, $^{1}J_{HH}$ = 13; $^{2}J_{HH}$ = 8.1; H34'), 3.55-3.44 (m, 1H, H34''), 4.08-4.00 (m, 1H, H35), 7.31 (t, 1H, $^{1}J_{HH}$ = 7.8; H7), 7.43 (t, 1H, $^{1}J_{HH}$ = 7.8; H8), 7.65 (dd, 1H, $^{1}J_{HH}$ = 7.8; $^{2}J_{HH}$ = 3.9, H9), 8.36 (d, 1H, $^{1}J_{HH}$ = 7.8, H6), 9.07 (s, 1H, H2). ^{13}C NMR (100 MHz, DMSO-d_6): δ 17.5 (C351), 43.4 (C34), 49.5 (C35), 101.7 (C3), 114.9 (C9), 116.8 (C6), 121.4 (C7), 124.7 (C8), 128.0 (C5a), 140.8 (C9a), 152.6 (C4), 156.4 (C2), 156.7 (C32), 158.7 (C10a), 161.0 (C31). MS (ESI) *m/z*: [(M+H)$^{+}$, 296]. Anal. Calcd for $C_{15}H_{13}N_5O_2$: C, 61.01; H, 4.44; N, 23.72. Found: C, 60.81; H, 4.56; N, 23.81.

3-(5-Methyl-3-oxo-3,4,5,6-tetrahydropyrazin-2-yl)-2-oxo-2*H*,6*H*-pyrimido[1,2-*a*]benzoimidazole (11b')

Yield 89% with 50% of isomer **11b'**, m.p. 160.78 or 197.26°C. ^{1}H NMR (400 MHz, DMSO-d_6): δ 1.22 (d, 3H, $^{1}J_{HH}$ = 6.6; H341), 3.55-3.44 (m, 1H, H35''), 3.84-3.78 (m, 1H, H34), 3.9 (dd, 1H, $^{1}J_{HH}$ = 14.7; $^{2}J_{HH}$ = 4.2; H35'), 7.31 (t, 1H, $^{1}J_{HH}$ = 7.8; H7), 7.43 (t, 1H, $^{1}J_{HH}$ = 7.8; H8), 7.65 (dd, 1H, $^{1}J_{HH}$ = 7.8; $^{2}J_{HH}$ = 3.9, H9), 8.36 (d, 1H, $^{1}J_{HH}$ = 7.8, H6), 8.96 (s, 1H, H2). ^{13}C NMR (100 MHz, DMSO-d_6): δ 17.3 (C341), 44.6 (C34), 49.3 (C35), 100.9 (C3), 114.8 (C9), 116.5 (C6), 121.3 (C7), 124.8 (C8), 127.9 (C5a), 140.5 (C9a), 152.5 (C4), 156.4 (C2), 157.0 (C32), 158.4 (C10a), 160.8 (C31). MS (ESI) *m/z*: [(M+H)$^{+}$, 296]. Anal. Calcd for $C_{15}H_{13}N_5O_2$: C, 61.01; H, 4.44; N, 23.72. Found: C, 60.81; H, 4.56; N, 23.81.

3-(3-Oxo-3,4,4a,5,6,7,8,8a-octahydroquinoxalin-2-yl)-2-oxo-2*H*,6*H*-pyrimido[1,2-*a*]benzoimidazole (11c)

Yield 91%, m.p. 231.06°C. ^{1}H NMR (400 MHz, DMSO-d_6): δ 1,50-1,28 (m, 4H, H34''+H35''+H36''+H37''); 1,81-1,65 (m, 2H, H35'+H36'); 1,98 (d, 1H, $^{1}J_{HH}$ = 11,2; H34'); 2,25 (d, 1H, $^{1}J_{HH}$ = 9,0; H37'); 3,39-3,27 (m, 1H, 2H); 7,34-7,30 (m, 1H, H7); 7,44 (ddd, $^{1}J_{HH}$ = 7,8; $^{2}J_{HH}$ = 7,6; $^{3}J_{HH}$ = 1,2; H8); 7,66-7,62 (m, 1H, H9); 8,37 (ddd, 1H, $^{1}J_{HH}$ = 8,1; $^{2}J_{HH}$ = 1,2; $^{3}J_{HH}$ = 0,7; H6); 8,85 (bs, 1H, H2); tautomer 1,50-1,28 (m, 4H, H34''+H35''+H36''+H37''); 1,81-1,65 (m, 2H, H35'+H36'); 1,98 (d, 1H, $^{1}J_{HH}$ = 11,2; H34'); 2,25 (d, 1H, $^{1}J_{HH}$ = 9,0; H37'); 3,39-3,27 (m, 1H, 2H); 7,34-7,30 (m, 1H, H7); 7,43 (ddd, $^{1}J_{HH}$ = 8,1; $^{2}J_{HH}$ = 7,3; $^{3}J_{HH}$ = 1,2; H8); 7,66-7,62 (m, 1H, H9); 8,36 (ddd, 1H, $^{1}J_{HH}$ = 7,8; $^{2}J_{HH}$ = 1,2; $^{3}J_{HH}$ = 0,7; H6); 8,85 (bs, 1H, H2). MS (ESI) *m/z*: [(M+H)$^{+}$, 336]. Anal. Calcd for $C_{18}H_{17}N_5O_2$: C, 64.47; H, 5.11; N, 20.88. Found: C, 64.22; H, 5.01; N, 20.56.

3-(3-Oxo-3,4-dihydroquinoxalin-2-yl)-2-oxo-2*H*,6*H*-pyrimido[1,2-*a*]benzoimidazole (11d)

Yield 52%, m.p. 377,22°C. ^{1}H NMR (400 MHz, DMSO-d_6): δ 7.31 (ddd, $^{1}J_{HH}$ = 8.1; $^{2}J_{HH}$ = 7.6; $^{3}J_{HH}$ = 1.2; H36), 7.40-7.35 (m, 2H, H7+H34), 7.51 (td, 1H, $^{1}J_{HH}$ = 8.1; $^{2}J_{HH}$ = 1.2; H35), 7.53 (ddd, 1H, $^{1}J_{HH}$ = 8.1; $^{2}J_{HH}$ = 7.3; $^{3}J_{HH}$ = 1.5; H8), 7.58 (d, 1H, $^{1}J_{HH}$ = 8.1; H9), 7.79 (dd, 1H, $^{1}J_{HH}$ = 8.1; $^{2}J_{HH}$ = 1.2; H37), 8.36 (s, 1H, H2), 8.49 (d, 1H, $^{1}J_{HH}$ = 7.8; H6), 12.29 (bs, 1H, NH33). ^{13}C NMR (100 MHz, DMSO-d_6): δ 110.4 (C3), 112.4 (C34), 115.0 (C9), 115.5 (C6), 121.9 (C7), 122.9 (C8), 126.0 (C36), 126.4 (C5a), 128.3 (C37), 129.8 (C35), 131.86 (C33a), 131.93 (C37a), 132.6 (C9a), 149.0 (C4), 152.0 (C32), 154.0 (C2), 154.1 (C10a),

157.0 (C31). MS (ESI) *m/z*: [(M+H)$^+$, 330]. Anal. Calcd for $C_{18}H_{11}N_5O_2$: C, 65.65; H, 3.37; N, 21.27. Found: C, 65.42; H, 3.50; N, 21.03.

3-(7-Methyl-3-oxo-3,4-dihydroquinoxalin-2-yl)-2-oxo-2*H*,6*H*-pyrimido[1,2-*a*]benzoimidazole (11e)

Yield 56% with 48% of isomer **11e**, Decomposition Temperature.. 300.00°C ^{1}H NMR (400 MHz, DMSO-*d*$_6$): δ 2.43 (s, 3H, H361), 7.25 (d, 1H, H35), 7.39-7.34 (m, 2H, H7+H34), 7.51 (t, 1H, $^1J_{HH}$ = 8.1; H8), 7.39 (s, 1H, H37), 7.59-7.58 (m, 1H, H9), 8.50 (s, 1H, H2), 12.22 (bs, 1H, NH33). ^{13}C NMR (100 MHz, DMSO-*d*$_6$): δ 21.0 (C361), 110.5 (C3), 112.4 (C9), 114.8 (C35), 115.5 (C6), 121.9 (C7), 126.0 (C8), 126.4 (C34), 128.1 (C37), 129.7 (C33a), 131.8 (C5a), 131.9 (C37a), 132.6 (C9a), 140.1 (C36), 149.0 (C4), 151.9 (C32), 153.9 (C2), 154.2 (C10a), 157.0 (C31). MS (ESI) *m/z*: [(M+H)$^+$, 344], [(M+Na)$^+$, 366]. Anal. Calcd for $C_{19}H_{13}N_5O_2$: C, 66.47; H, 3.82; N, 20.40. Found: C, 66.91; H, 4.11; N, 20.19.

3-(6-Methyl-3-oxo-3,4-dihydroquinoxalin-2-yl)-2-oxo-2*H*,6*H*-pyrimido[1,2-*a*]benzoimidazole (11e')

Yield 56% with 48% of isomer **11e'**,Decomposition Temperature . 300.00°C ^{1}H NMR (400 MHz, DMSO-*d*$_6$): δ 2.40 (s, 3H, H351), 7.14-7.12 (m, 2H, H34+H36), 7.39-7.34 (m, 2H, H7), 7.51 (t, 1H, $^1J_{HH}$ = 8.1; H8), 7.59-7.58 (m, 1H, H9), 7.66 (d, 1H, $^1J_{HH}$ = 8.3; H37), 8.48 (s, 1H, H2), 12.22 (bs, 1H, NH33).

^{13}C NMR (100 MHz, DMSO-*d*$_6$): δ 20.2 (C351), 110.5 (C3), 112.4 (C9), 114.6 (C36), 115.5 (C6), 121.9 (C7), 124.4 (C34), 126.0 (C8), 127.9 (C37), 130.2 (C33a), 131.0 (C37a), 131.8 (C5a), 132.2 (C35), 132.6 (C9a), 149.0 (C4), 152.8 (C32), 153.9 (C2), 154.2 (C10a), 157.0 (C31). MS (ESI) *m/z*: [(M+H)$^+$, 344],

[(M+Na)$^+$, 366] Anal. Calcd for $C_{19}H_{13}N_5O_2$: C, 66.47; H, 3.82; N, 20.40. Found: C, 66.91; H, 4.11; N, 20.19.

3.3. General Procedure for Preparation of 12g,h and 13g,h

A mixture of sodium metal (0.5 mmol) and dry ethanol (3 mL) was stirred for 5 min in order to form sodium ethoxide. Acetamidine hydrochloride (**8g**) or benzamidine hydrochloride (**8h**) (0.5 mmol) was then added. The mixture was kept under agitation for 5 min, then the α-ketoester **5** or **6** (0.5 mmol) was added, and the reaction mixture was stirred at rt for 0.5 h. The ethanol was evaporated under reduced pressure and the resulting solid was washed with ethyl acetate (3 × 1 mL) followed by water (2 × 1 mL). The washing with water is required due to the formation of sodium chloride through the neutralization of the hydrogen chloride present in the starting amidine with the base sodium ethoxide.

_N_-(1-Iminoethyl)-2-oxo-2-(4-oxo-4_H_-pyrido[1,2-a]pyrimidin-3-yl)acetamide (12g)

Yield 67%, 137.06°C. ^{1}H NMR (400 MHz, DMSO-d_6): δ 2.09 (s, 3H, H35), 7.37 (td, 1H, $^1J_{HH}$ = 7.1; $^2J_{HH}$ = 0.7; H8), 7.71 (ddd, 1H, $^1J_{HH}$ = 8.9; $^2J_{HH}$ = 1.3; $^3J_{HH}$ = 0.7; H6), 7.96 (ddd, 1H, $^1J_{HH}$ = 8.9; $^2J_{HH}$ = 6.6 Hz; $^3J_{HH}$ = 1.6; H7), 8.64 (s, 1H, H2), 8.89 (ddd, 1H, $^1J_{HH}$ = 7.1; $^2J_{HH}$ = 1.5; $^3J_{HH}$ = 0.7; H9). ^{13}C NMR (100 MHz, DMSO-d_6): δ 16,51 (C35), 114,7 (C3), 116,7 (C8), 125,9 (C6), 126,6 (C9), 137,2 (C7), 148,3 (C4), 150,5 (C9a), 151,9 (C2), 154,7 (C32), 169,1 (C34), 184,8 (C31). MS (ESI) *m/z*: [(M+H)$^+$, 259], [(M+Na)$^+$, 281]. Anal. Calcd for $C_{12}H_{10}N_4O_3$: C, 55.81; H, 3.90; N, 21.70. Found: C, 55.56; H, 3.94; N, 21.47.

_N_-(Imino(phenyl)methyl)-2-oxo-2-(4-oxo-4_H_-pyrido[1,2-a]pyrimidin-3-yl)acetamide (12h)

Yield 66%, m.p. 151.55°C. ^{1}H NMR (400 MHz, DMSO-d_6): δ 7.37 (td, 1H, $^{1}J_{HH}$ = 7.1; $^{2}J_{HH}$ = 1.5; H8), 7.54 (t, 2H, Ph), 7.62 (t, 1H, Ph), 7.74 (d, 1H, $^{1}J_{HH}$ = 9.0; H6), 7.96 (ddd, 1H, $^{1}J_{HH}$ = 9.0; $^{2}J_{HH}$ = 6.6; $^{3}J_{HH}$ = 1.5; H7), 8.08 (d, 2H, Ph), 8.75 (s, 1H, H2), 8.86 (ddd, 1H, $^{1}J_{HH}$ = 7.1; $^{2}J_{HH}$ = 1.5; $^{3}J_{HH}$ = 0.7; H9). ^{13}C NMR (100 MHz, DMSO-d_6): δ 114,6 (C3), 116,9 (C8), 126,0 (C6), 126,7 (C9), 127,7 + 128,7 + 128,8 + 132,6 (Ph), 137,5 (C7), 150,7 (C4), 152,1 (C9a), 154,8 (C2), 161,9 (C32+C34), 183,5 (C31). MS (ESI) *m/z*: [(M+H)$^+$, 321], [(M+Na)$^+$, 343]. Anal. Calcd for $C_{17}H_{12}N_4O_3$: C, 63.75; H, 3.78; N, 17.49. Found: C, 63.66; H, 4.03; N, 17.21.

_N_-(1-Iminoethyl)-2-oxo-2-(5-oxo-5_H_-thiazolo[3,2-_a_]pyrimidin-6-yl)acetamide (13g)

Yield 69%,Decomposition Temperature . 162,43°C ^{1}H NMR (400 MHz, DMSO-d_6): δ 2.07 (s, 3H, H35), 7.60 (d, 1H, $^{1}J_{HH}$ = 4.9; H7), 7.97 (d, 1H, $^{1}J_{HH}$ = 4.9; H6), 8,34 (s, 1H, H2). ^{13}C NMR (100 MHz, DMSO-d_6): δ 16.17 (C35), 116.0 (C7), 119.2 (C3), 121.3 (C6), 150.4 (C2), 155.3 (C4 + C32), 162.7 (C8a + C34), 183.7 (C31). MS (ESI) *m/z*: [(M+H)$^+$, 265], [(M+Na)$^+$, 287]. Anal. Calcd for $C_{10}H_8N_4O_3S$: C, 45.45; H, 3.05; N, 21.20. Found: C, 45.15; H, 2.88; N, 21.31.

_N_-(Imino(phenyl)methyl)-2-oxo-2-(5-oxo-5_H_-thiazolo[3,2-_a_]pyrimidin-6-yl)acetamide (13h)

Yield 51%,Decomposition Temperature . 208,11°C ^{1}H NMR (400 MHz, DMSO-d_6): δ 7.55 (t, 2H, Ph), 7.66-7.62 (m, 2H, H7 + Ph), 7.96 (d, 1H, $^{1}J_{HH}$ = 4.4; H6),

8.06 (d, 2H, Ph), 8.46 (s, 1H, H2). ^{13}C NMR (100 MHz, DMSO-d_6): δ 114.3 (C7), 116.1 (C3), 121.4 (C6), 127.4 + 128.5 + 132.2 (Ph), 150.5 (C2), 155.4 (C4 + C32), 162.8 (C8a + C34), 182.4 (C31). MS (ESI) *m/z*: [(M+H)$^+$, 326], [(M+Na)$^+$, 349]. Anal. Calcd for $C_{15}H_{10}N_4O_3S$: C, 55.21; H, 3.09; N, 17.17. Found: C, 55.59; H, 2.92; N, 16.95.

4. ^{1}H and ^{13}C NMR spectra

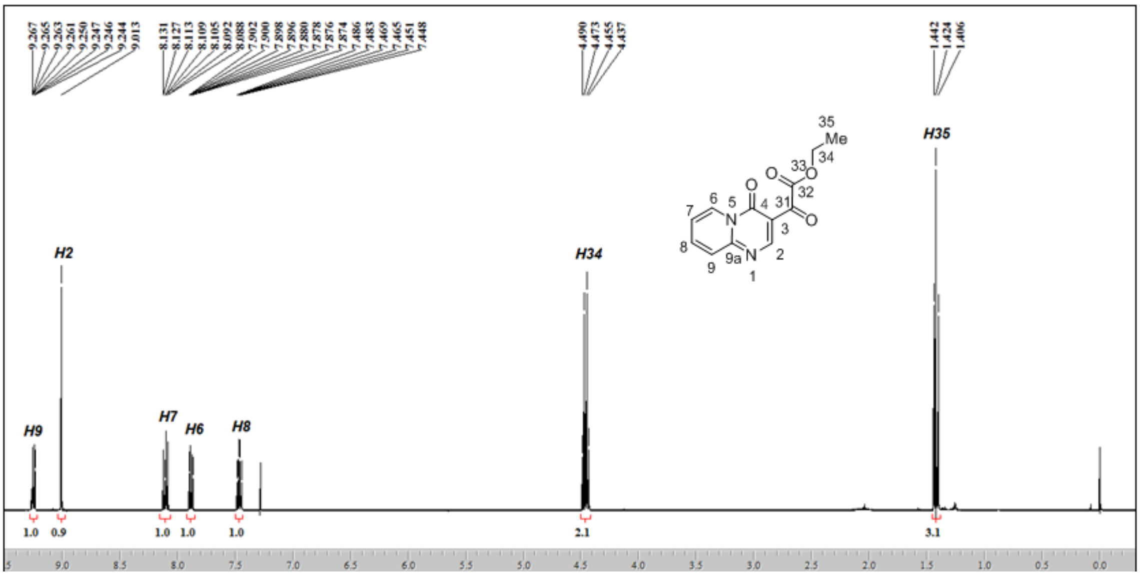

^{1}H NMR spectrum of compound **5** in $CDCl_3$ at 25 °C.

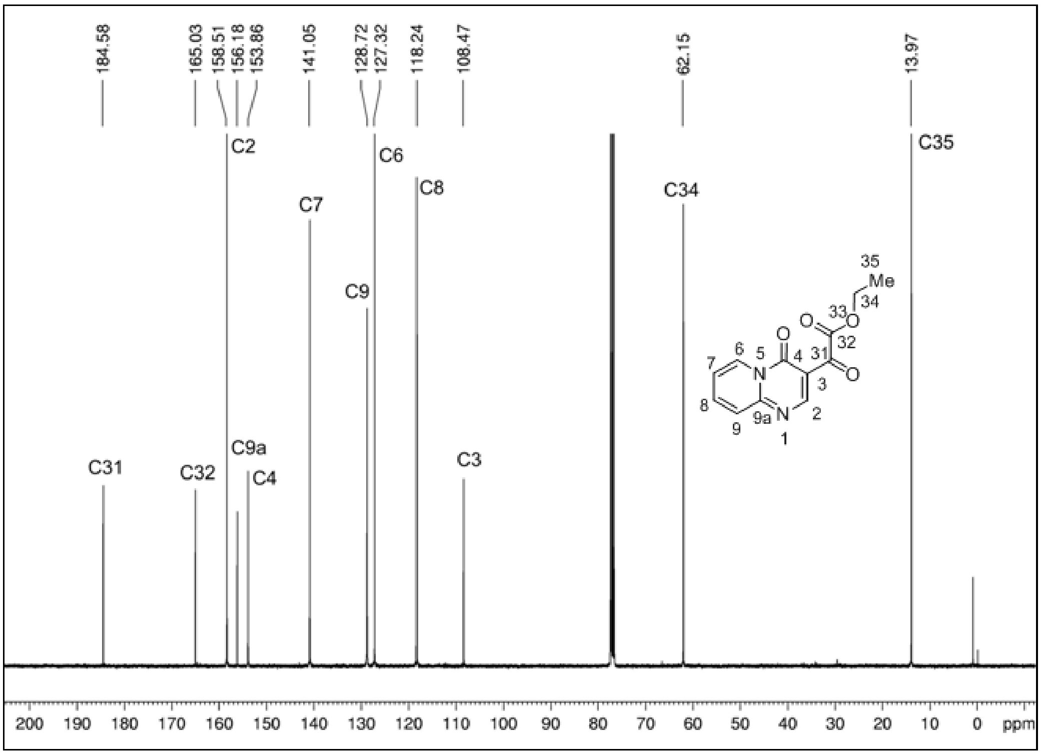

^{13}C NMR spectrum of compound **5** in $CDCl_3$ at 25 °C.

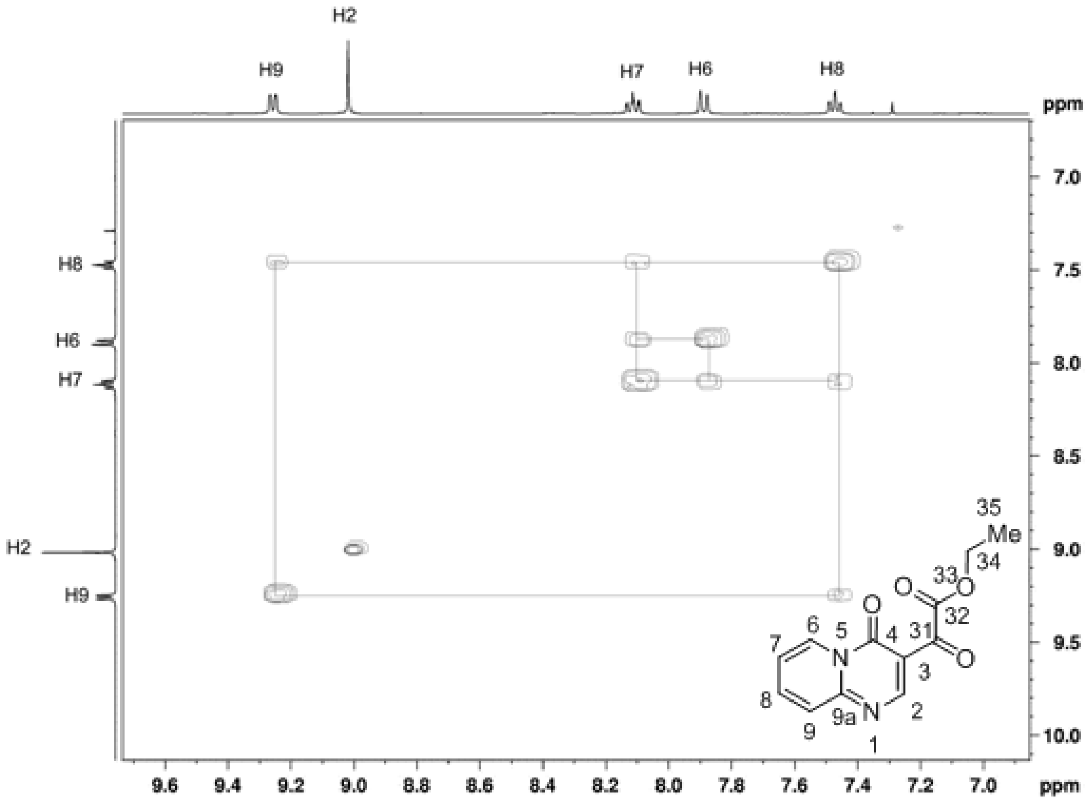

COSY spectrum of compound **5** in $CDCl_3$ at 25 °C.

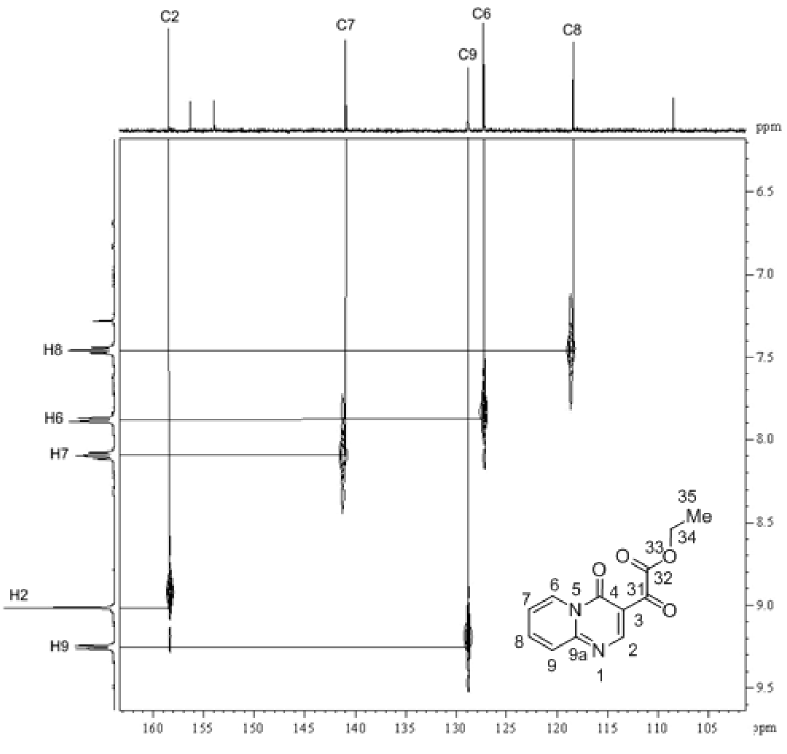

HETCOR spectrum of compound **5** in $CDCl_3$ at 25 °C.

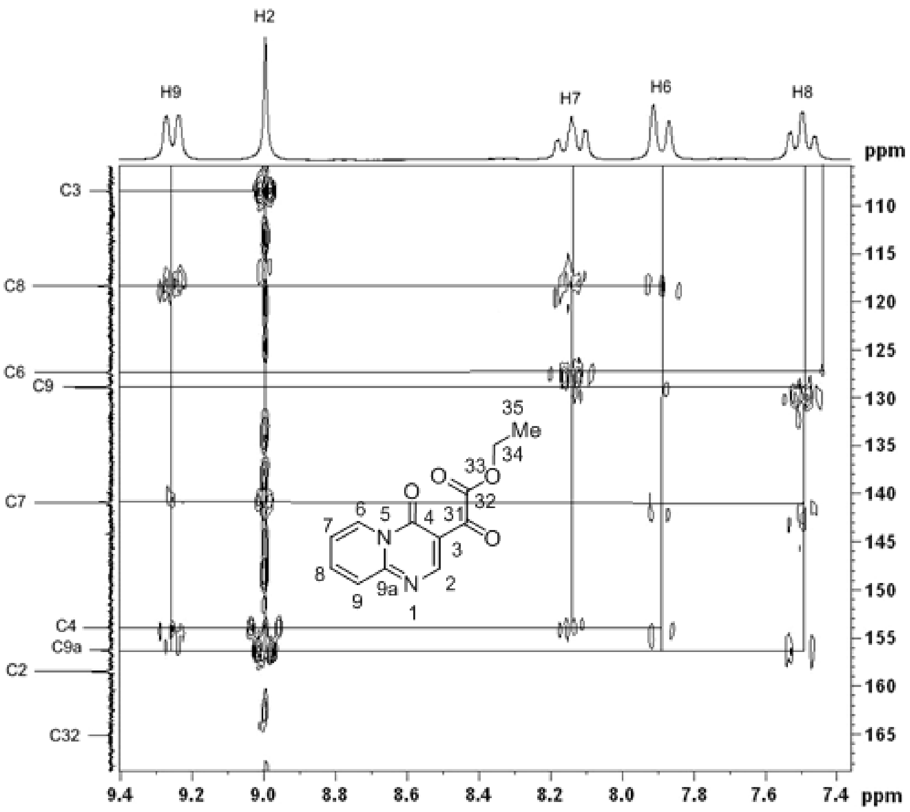

HMBC spectrum of compound **5** in $CDCl_3$ at 25 °C.

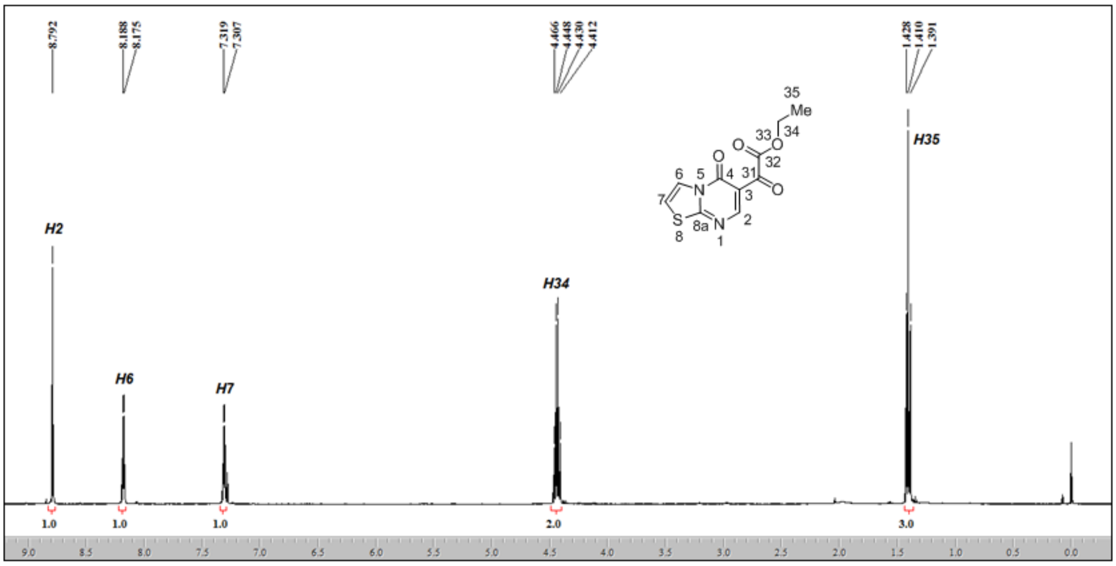

^{1}H NMR spectrum of compound **6** in $CDCl_3$ at 25 °C.

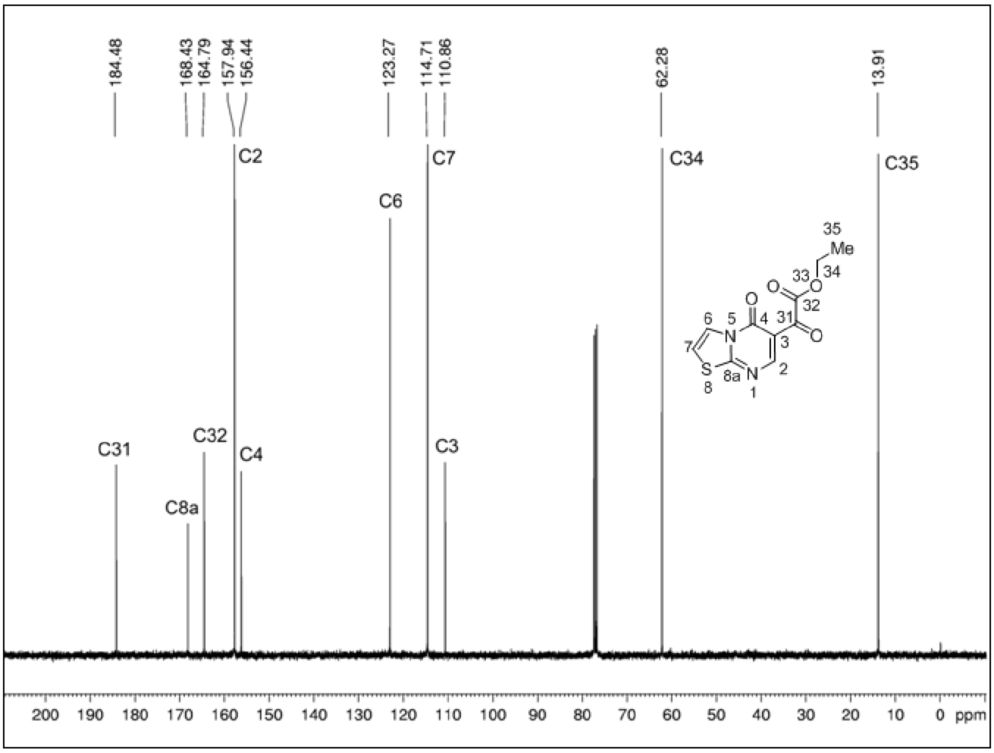

^{13}C NMR spectrum of compound **6** in $CDCl_3$ at 25 °C.

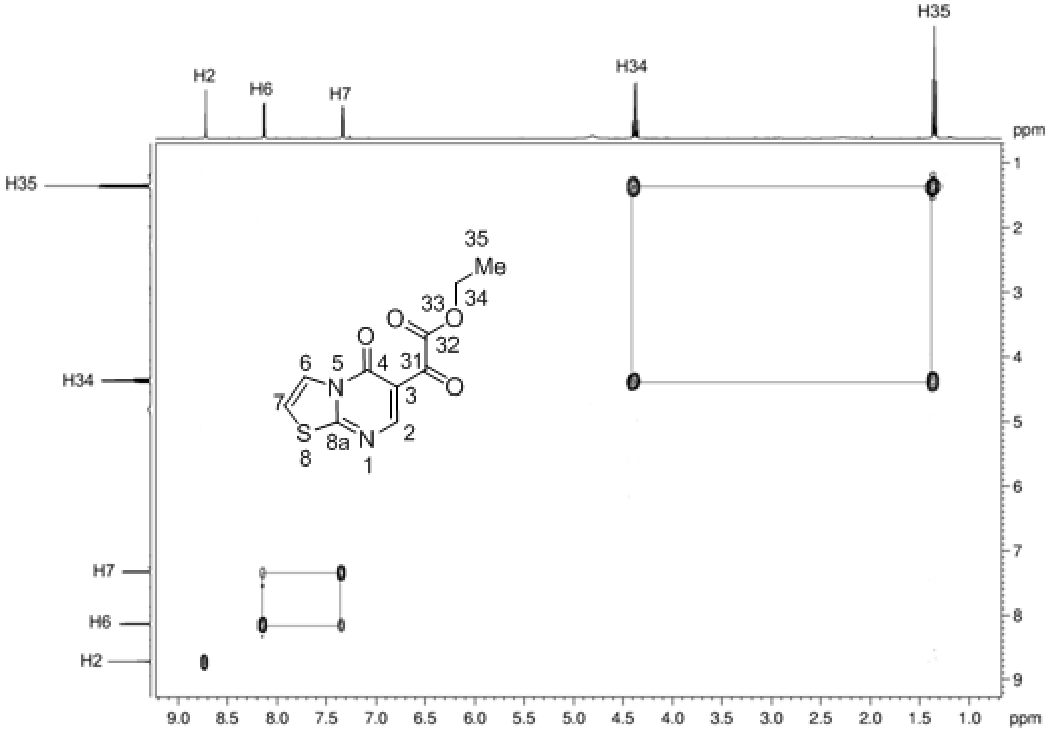

COSY spectrum of compound **6** in $CDCl_3$ at 25 °C.

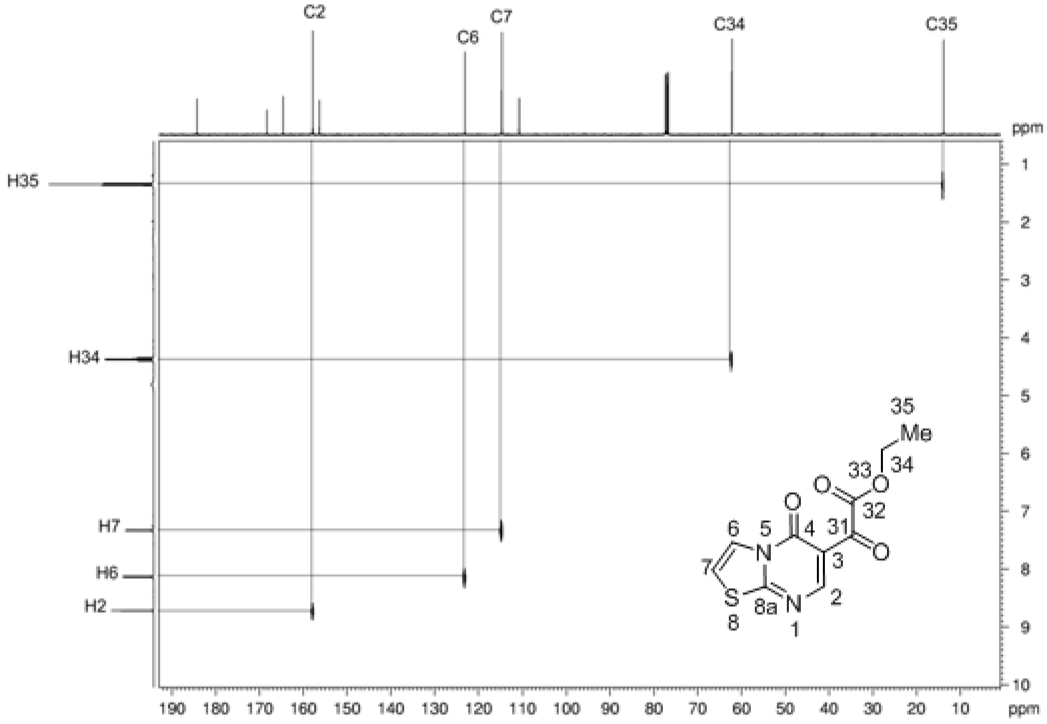

HETCOR spectrum of compound **6** in $CDCl_3$ at 25 °C.

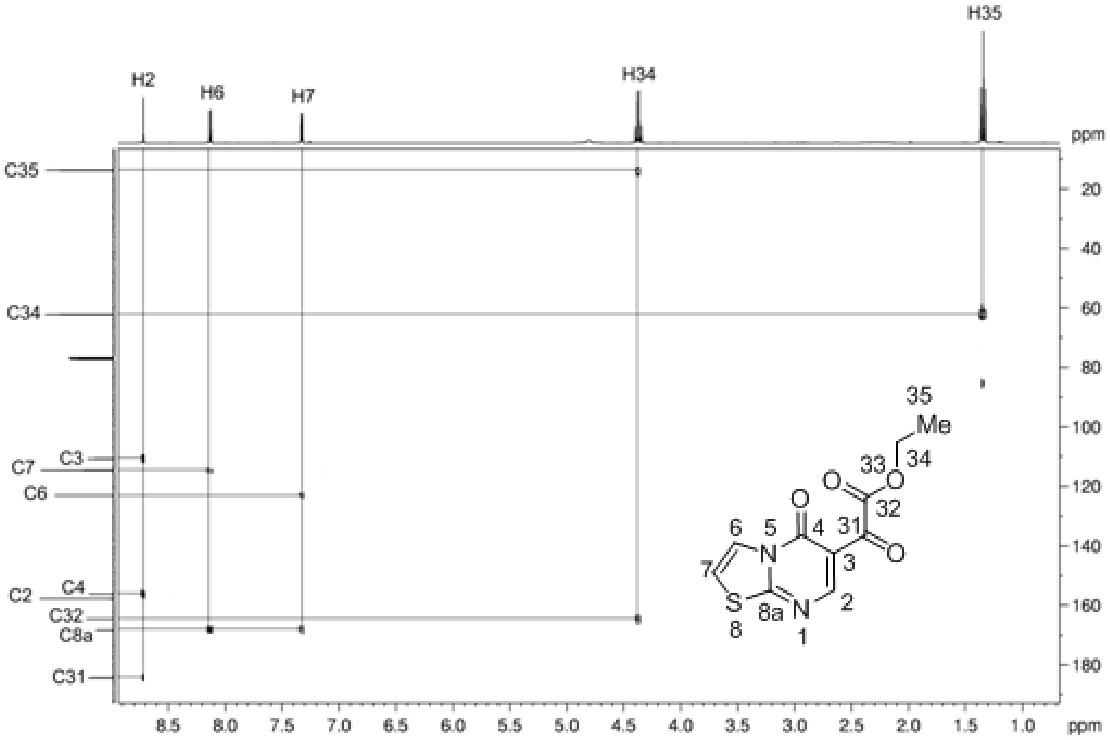

HMBC spectrum of compound **6** in $CDCl_3$ at 25 °C.

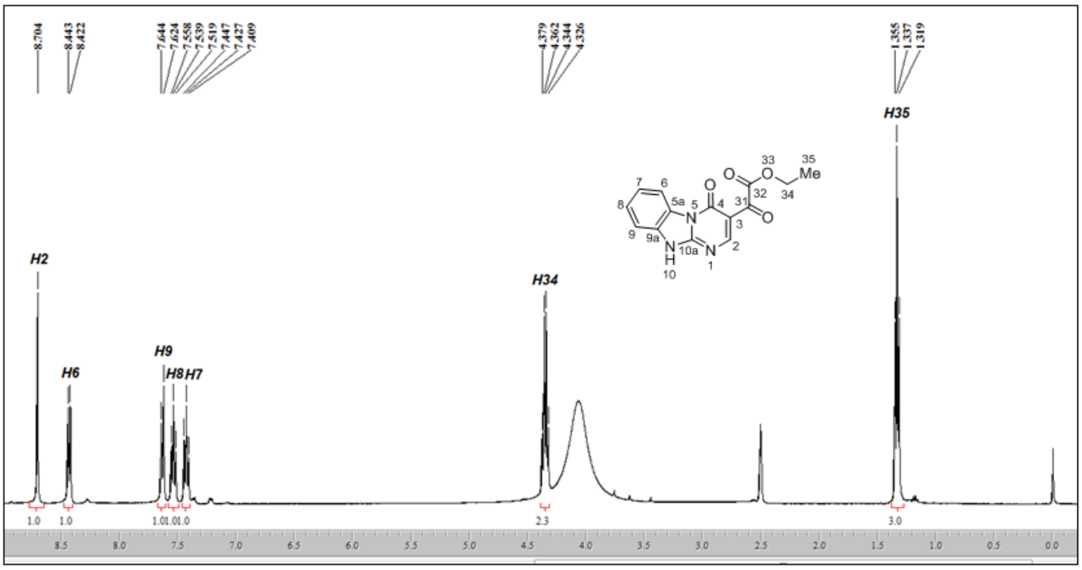

^{1}H NMR spectrum of compound **7** in DMSO-d_6 at 25 °C.

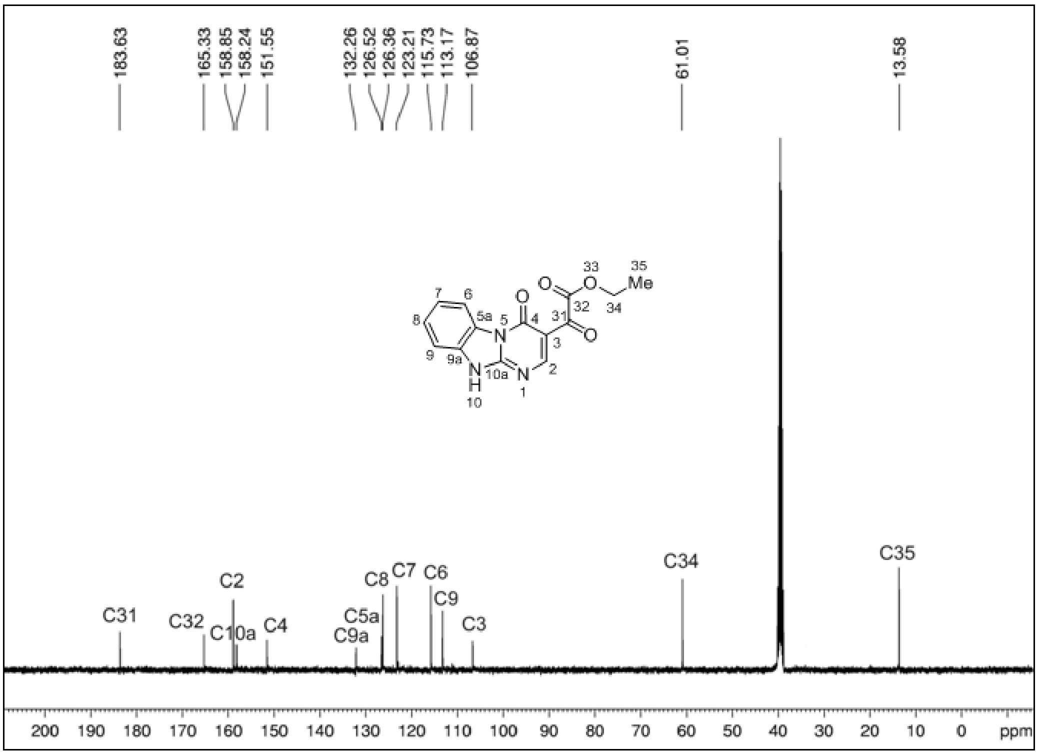

^{13}C NMR spectrum of compound **7** in DMSO-d_6 at 25 °C.

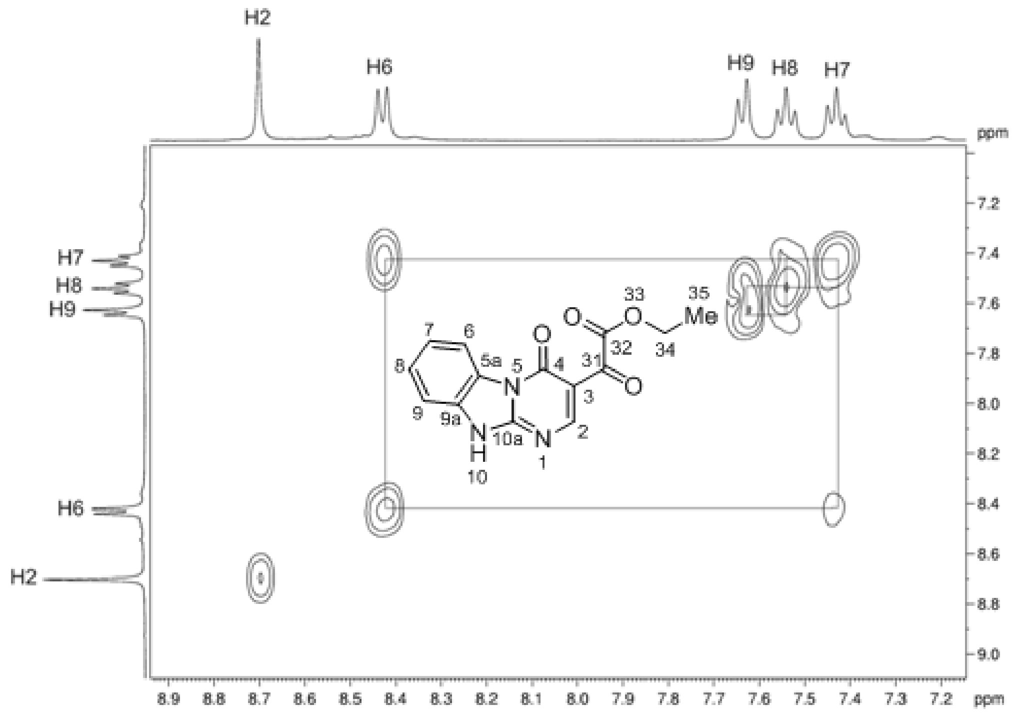

COSY spectrum of compound **7** in DMSO-d_6 at 25 °C.

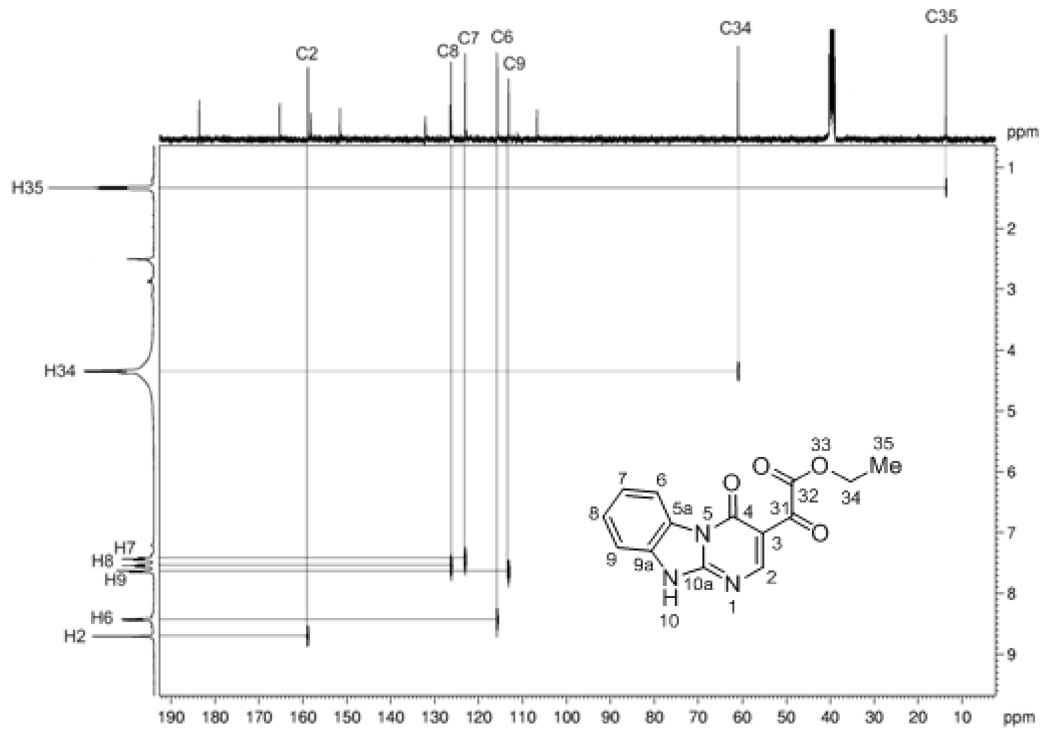

HETCOR spectrum of compound **7** in DMSO-d_6 at 25 °C.

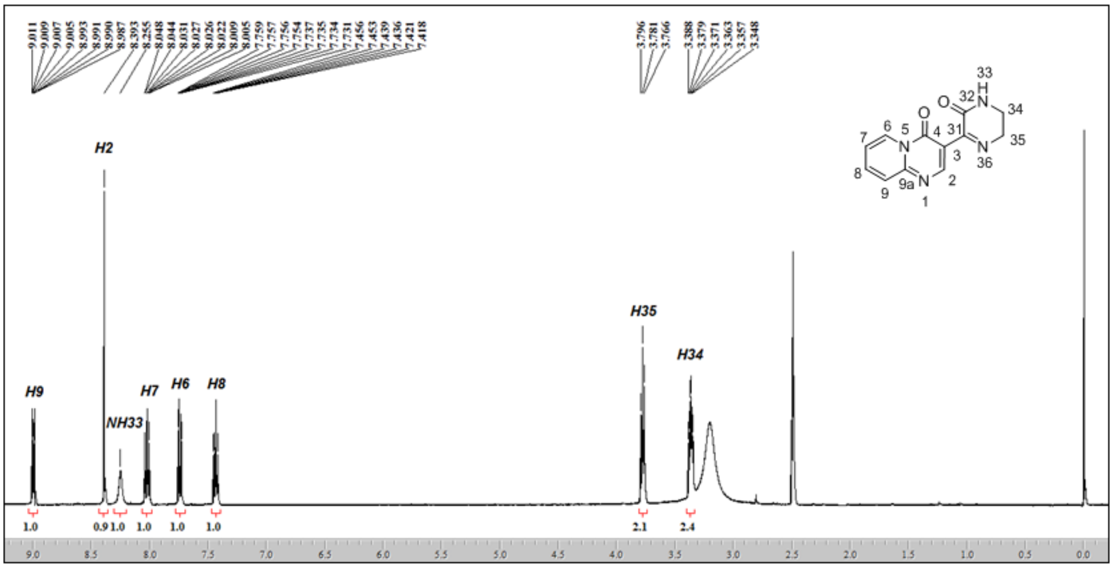

^{1}H NMR spectrum of compound **9a** in DMSO-d_6 at 25 °C.

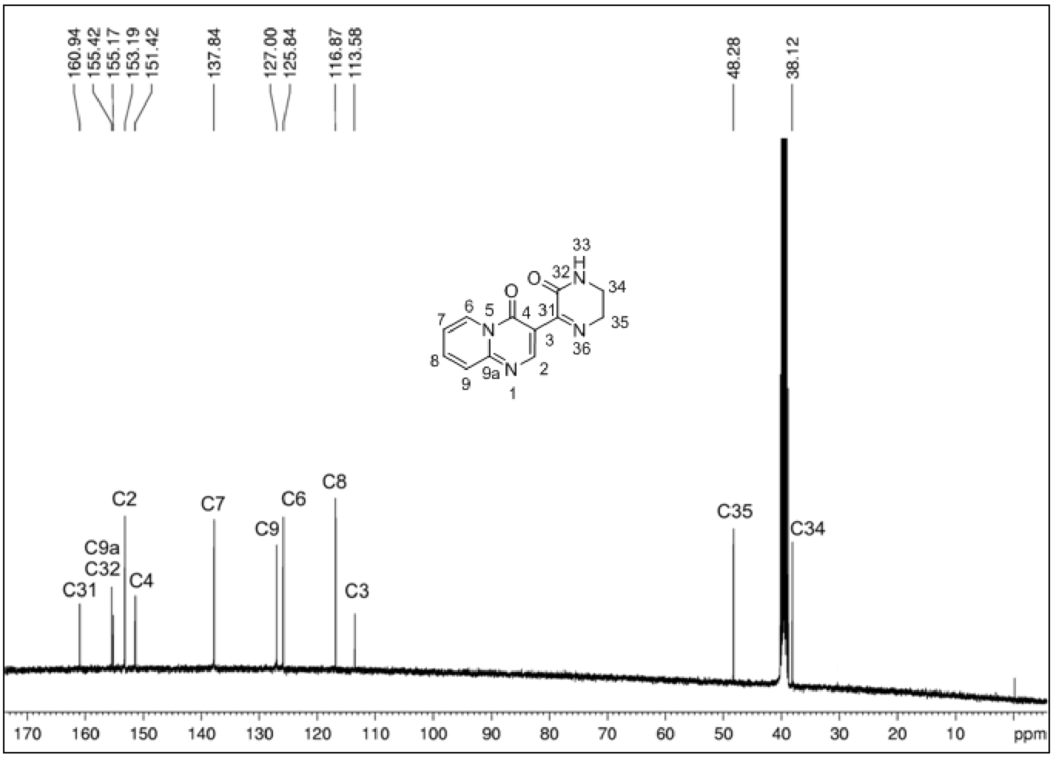

^{13}C NMR spectrum of compound **9a** in DMSO-d_6 at 25 °C.

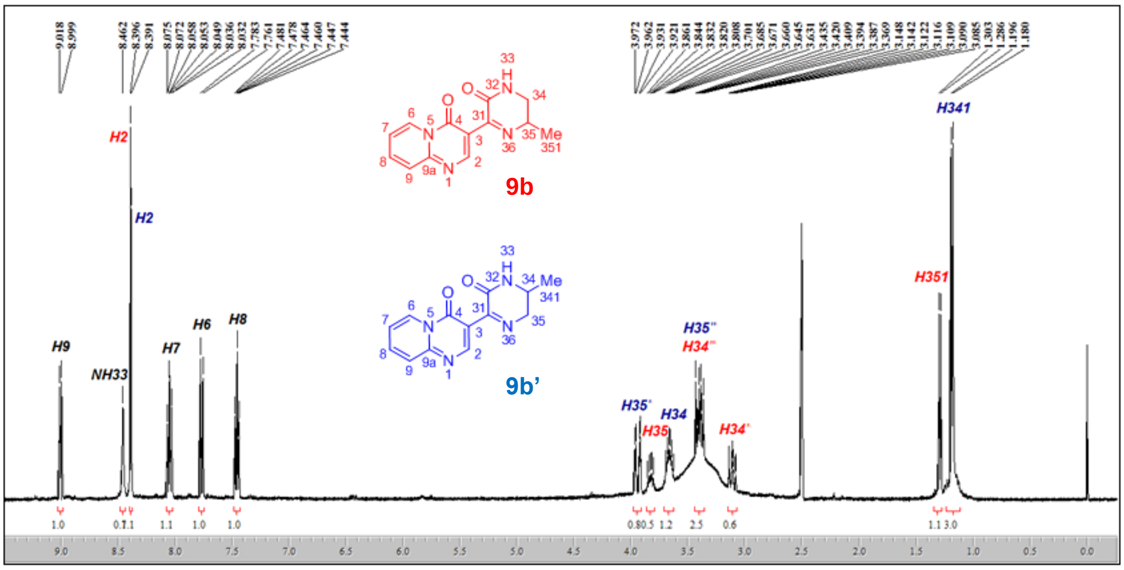

^{1}H NMR spectrum of compound **9b** and **9b’** in DMSO-d_6 at 25 °C.

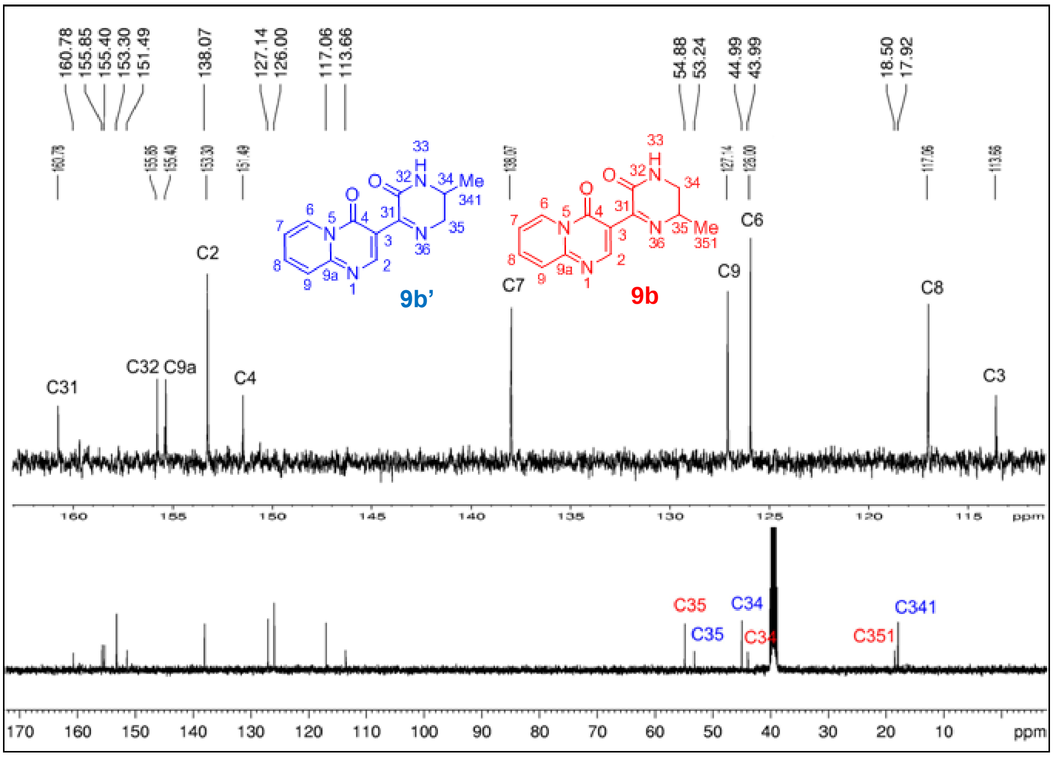

^{13}C NMR spectrum of compound **9b** and **9b’** in DMSO-d_6 at 25 °C.

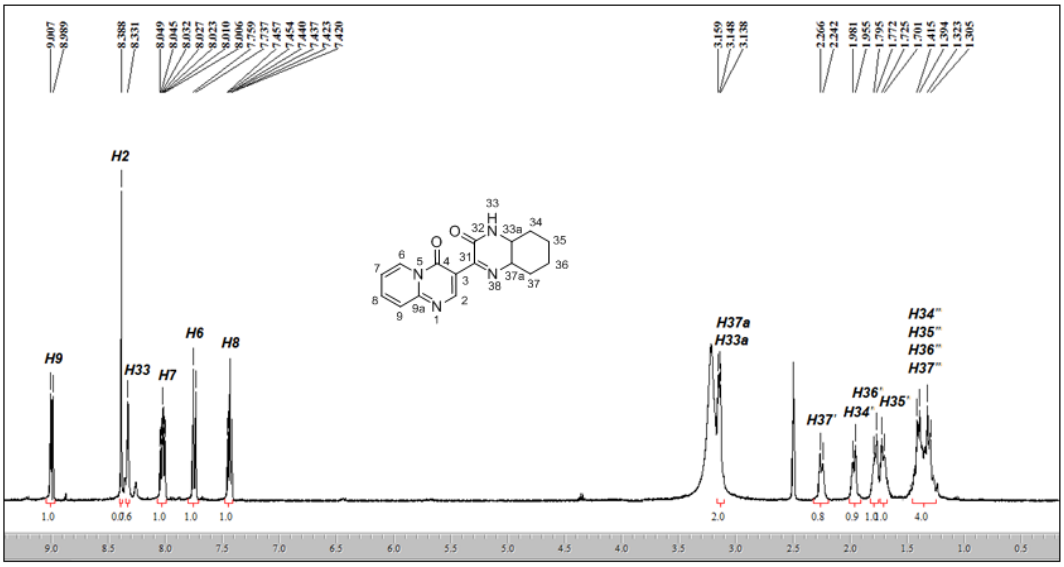

^{1}H NMR spectrum of compound **9c** in DMSO-d_6 at 25 °C.

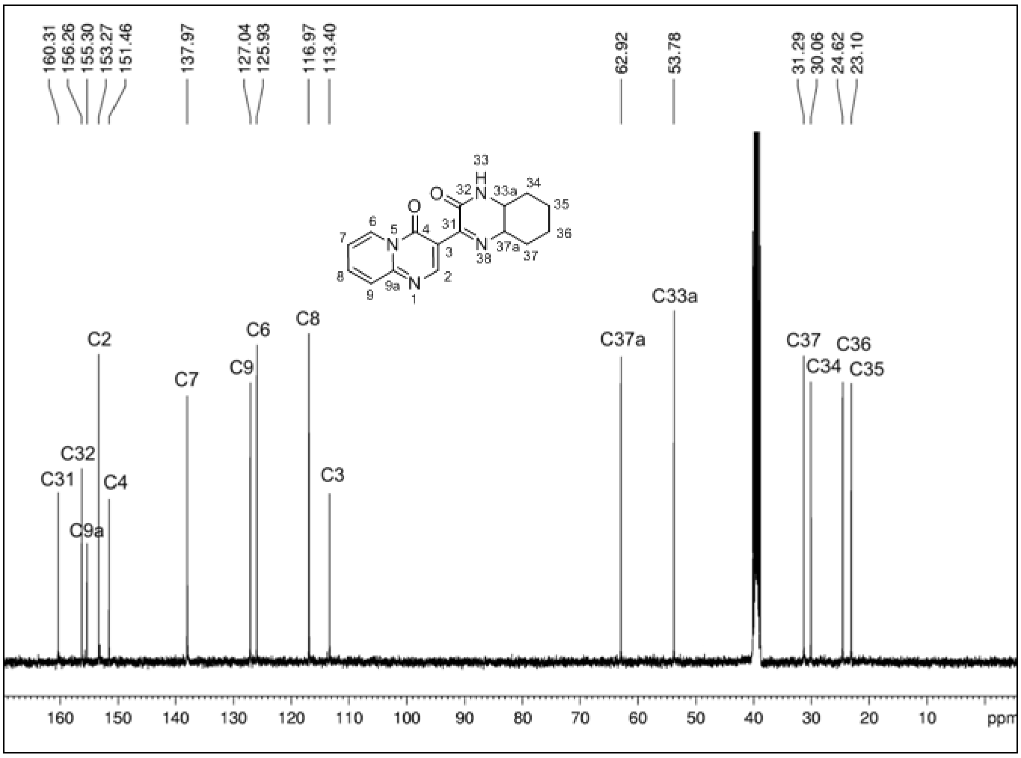

^{13}C NMR spectrum of compound **9c** in DMSO-d_6 at 25 °C.

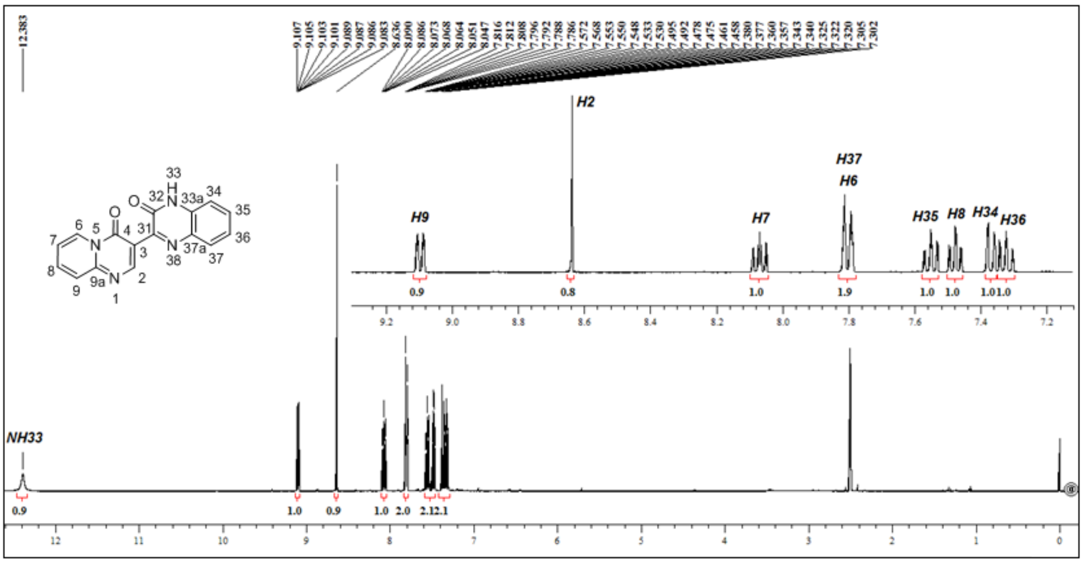

^{1}H NMR spectrum of compound **9d** in DMSO-d_6 at 25 °C.

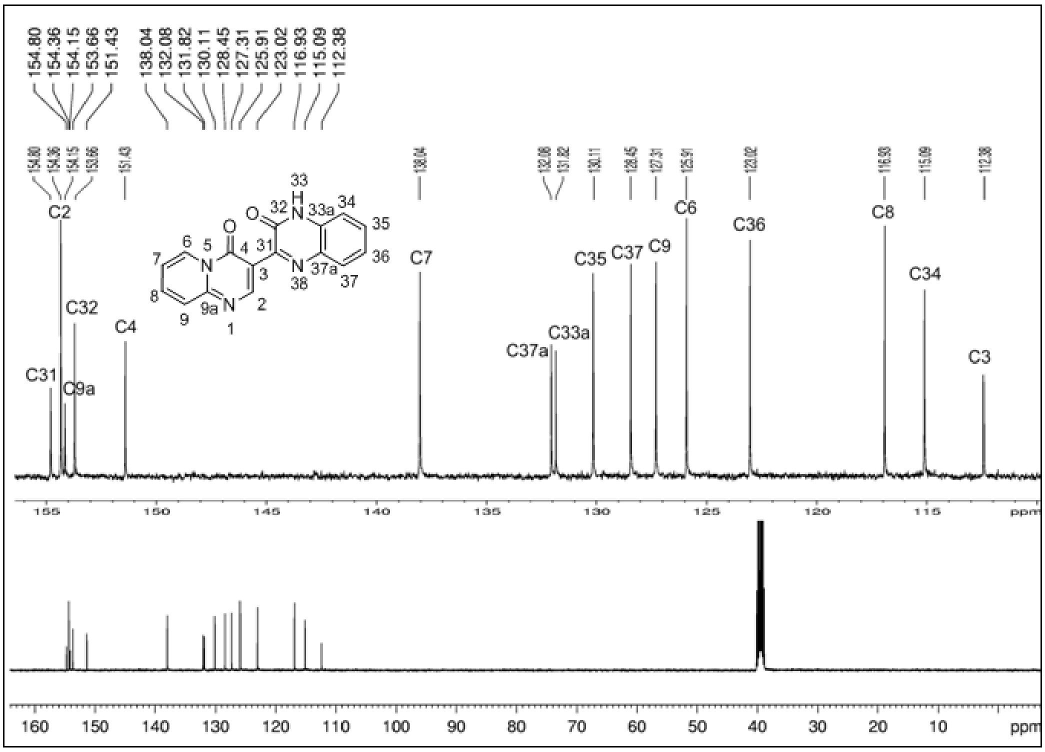

NMR

^{13}C NMR spectrum of compound **9d** in DMSO-d_6 at 25 °C.

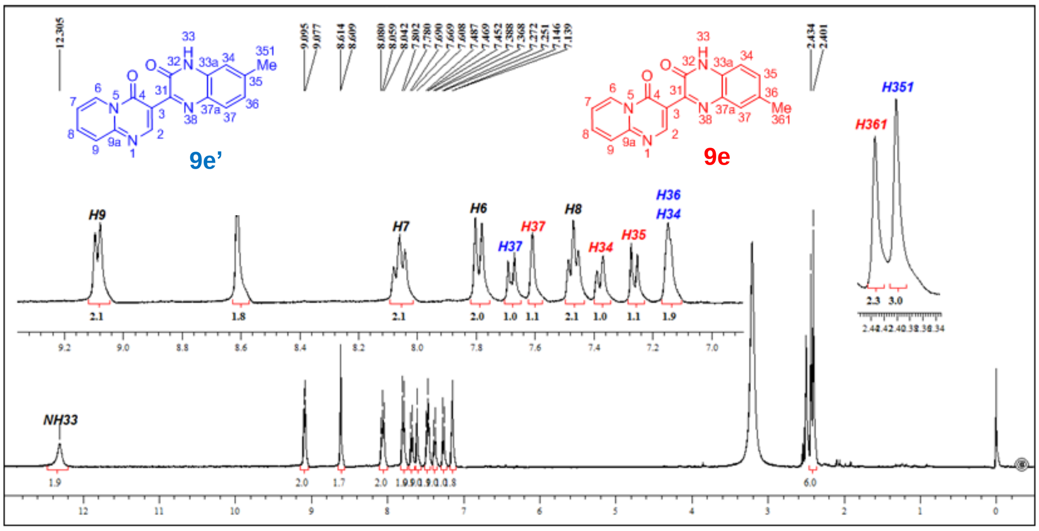

^{1}H NMR spectrum of compound **9e** and **9e’** in DMSO-d_6 at 25 °C.

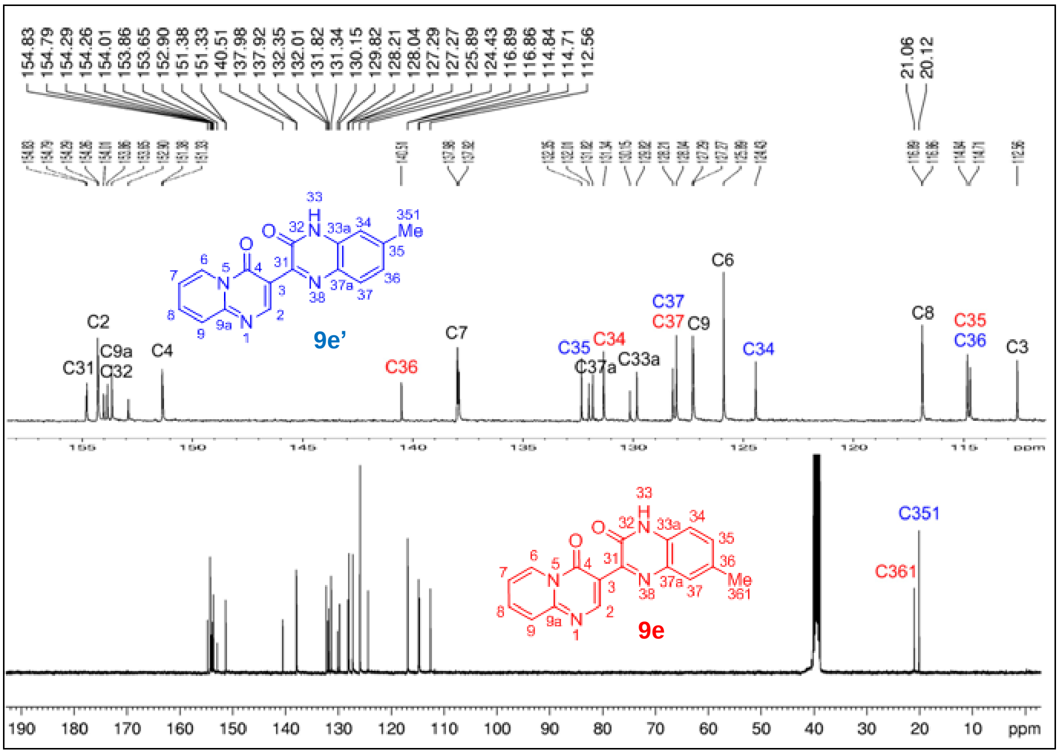

^{13}C NMR spectrum of compound **9e** and **9e’** in DMSO-d_6 at 25 °C.

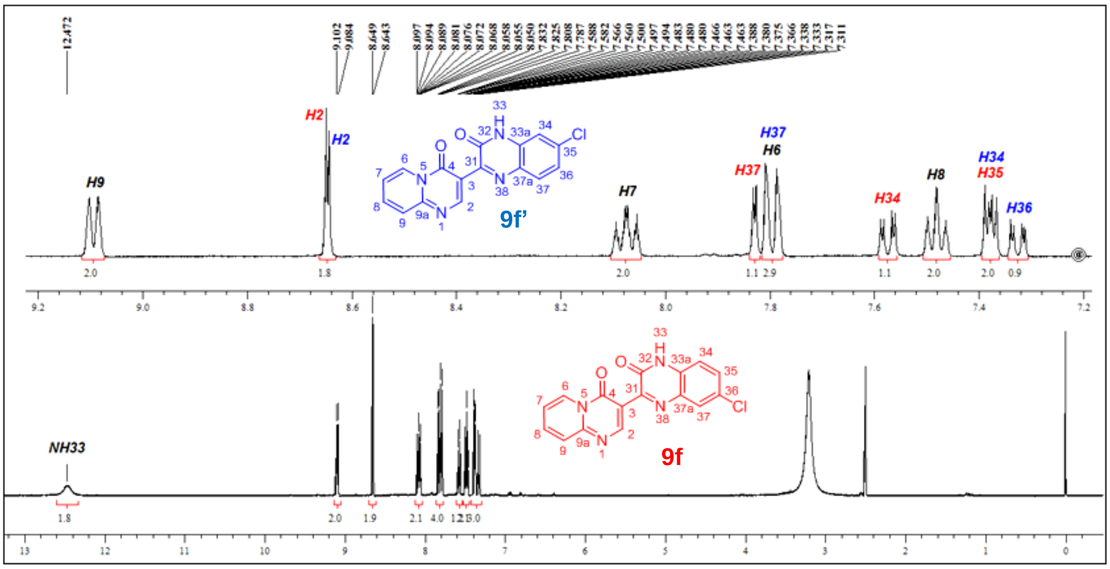

^{1}H NMR spectrum of compound **9f** and **9f'** in DMSO-d_6 at 25 °C.

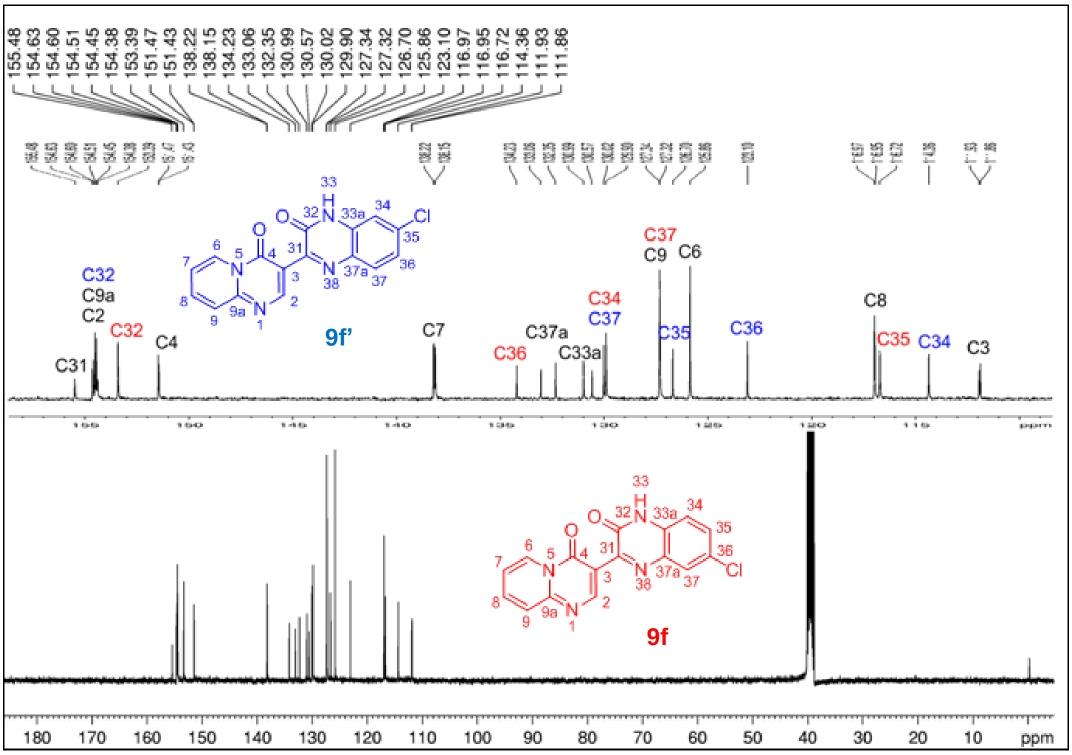

^{13}C NMR spectrum of compound **9f** and **9f'** in DMSO-d_6 at 25 °C.

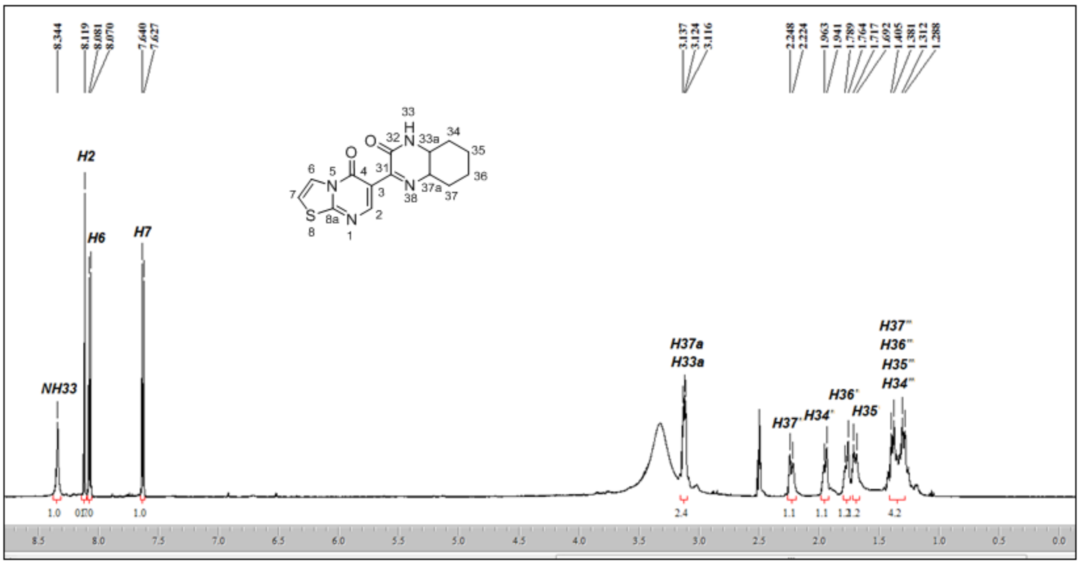

^{1}H NMR spectrum of compound **10c** in DMSO-d_6 at 25 °C.

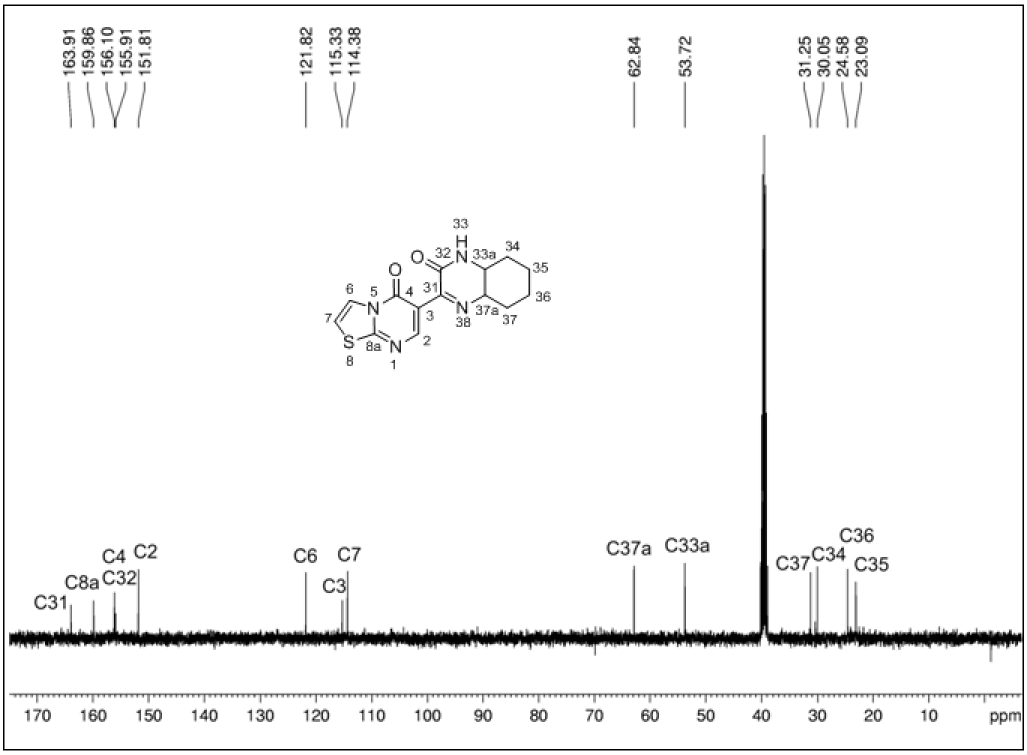

^{13}C NMR spectrum of compound **10c** in DMSO-d_6 at 25 °C.

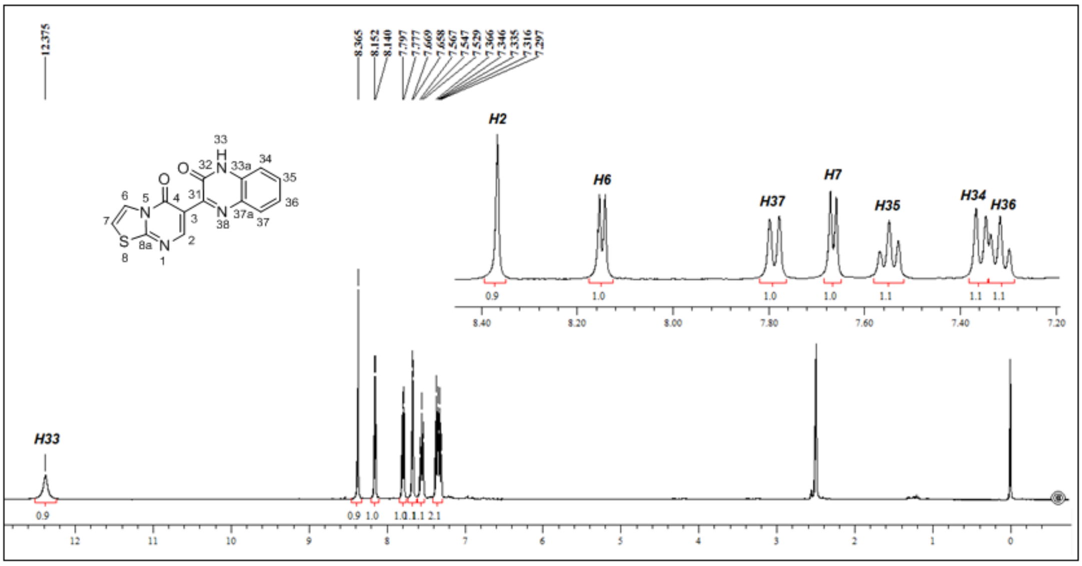

^{1}H NMR spectrum of compound **10d** in DMSO-d_6 at 25 °C.

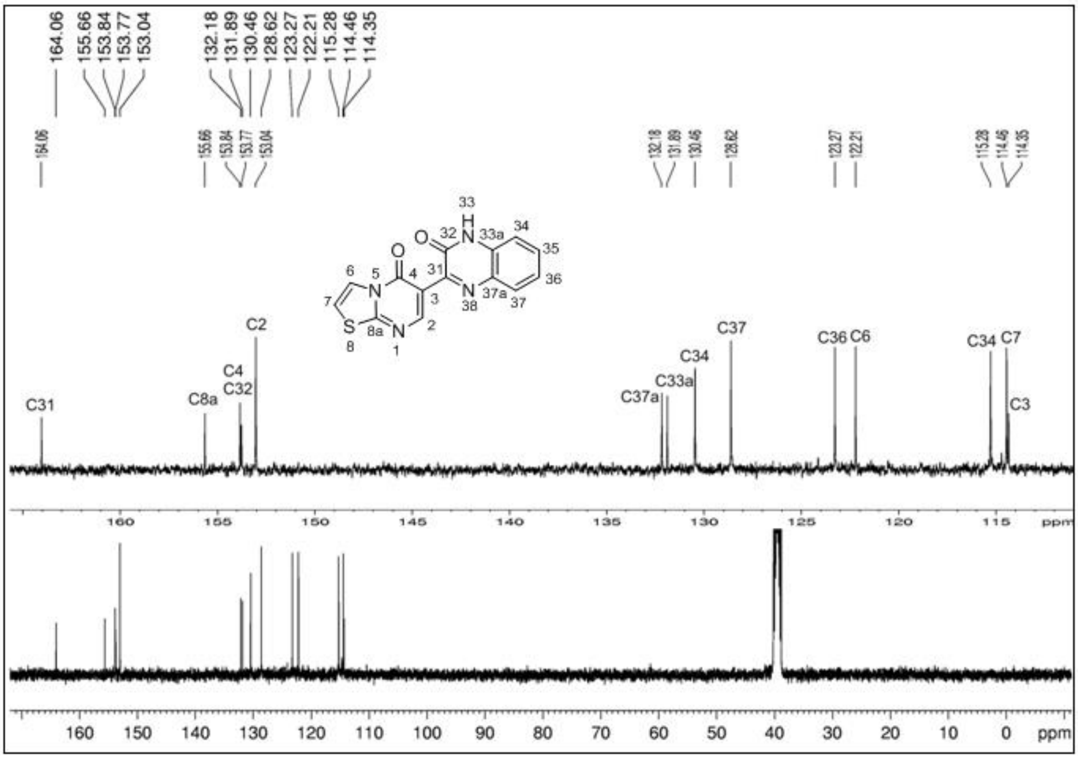

^{13}C NMR spectrum of compound **10d** in DMSO-d_6 at 25 °C.

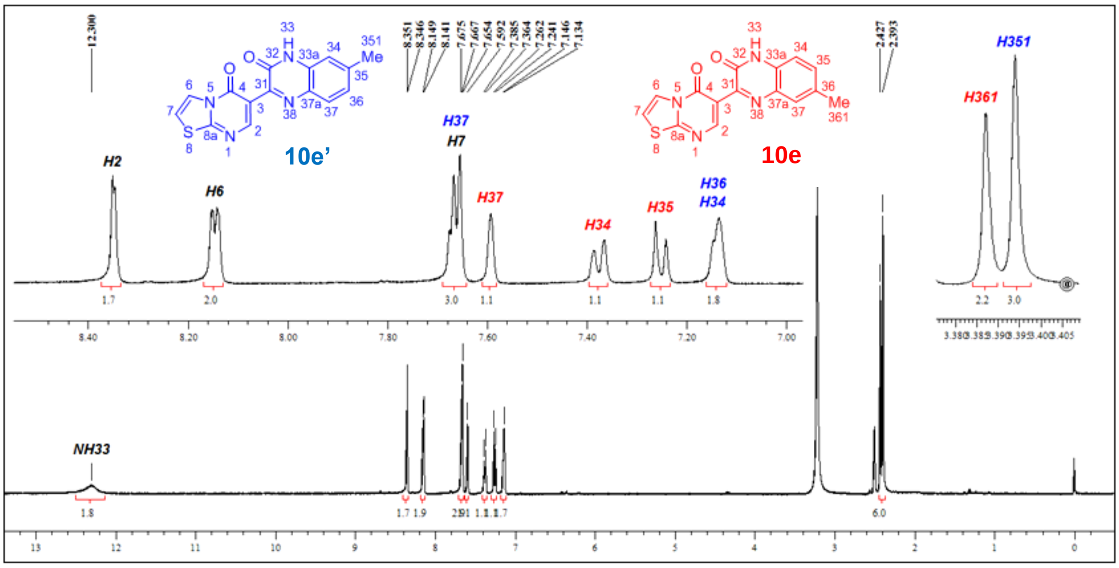

^{1}H NMR spectrum of compound **10e** and **10e'** in DMSO-d_6 at 25 °C.

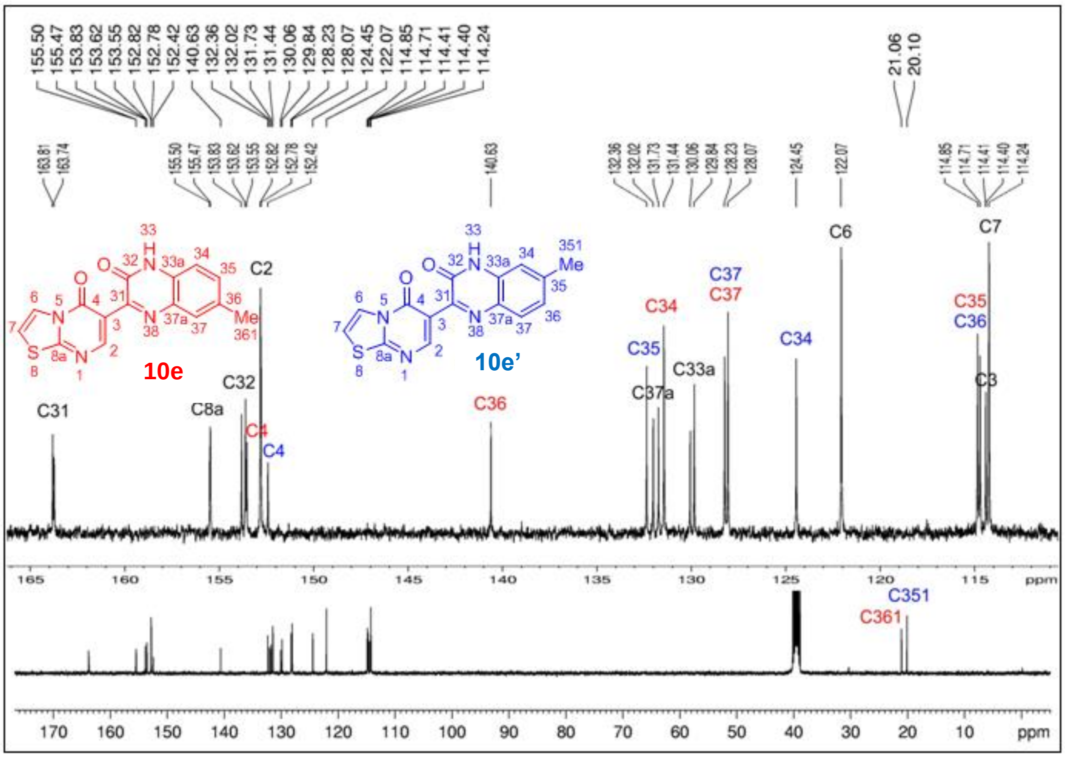

^{13}C NMR spectrum of compound **10e** and **10e'** in DMSO-d_6 at 25 °C.

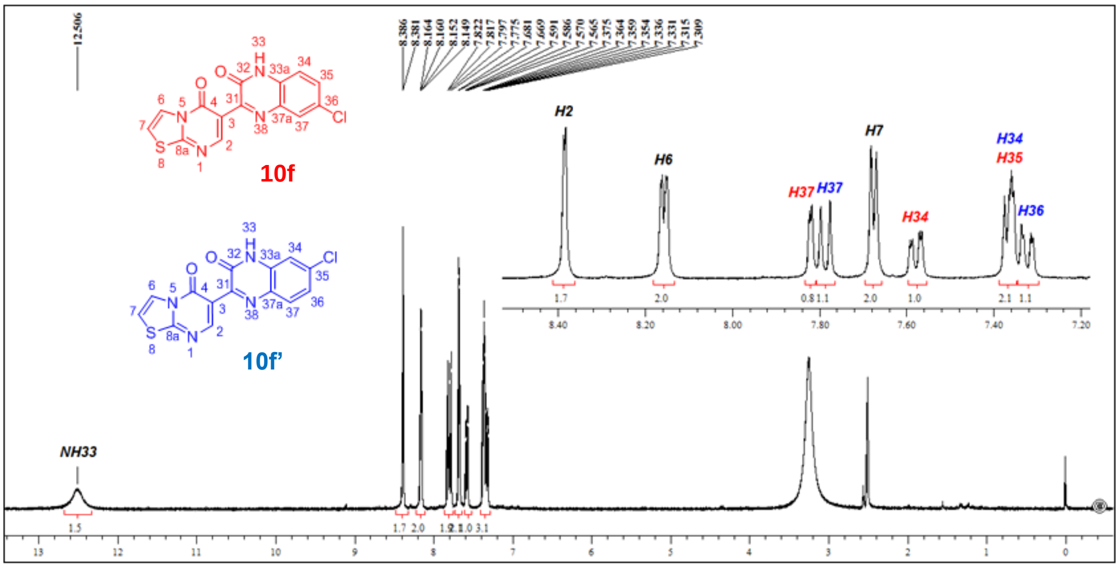

^{1}H NMR spectrum of compound **10f** and **10f'** in DMSO-d_6 at 25 °C.

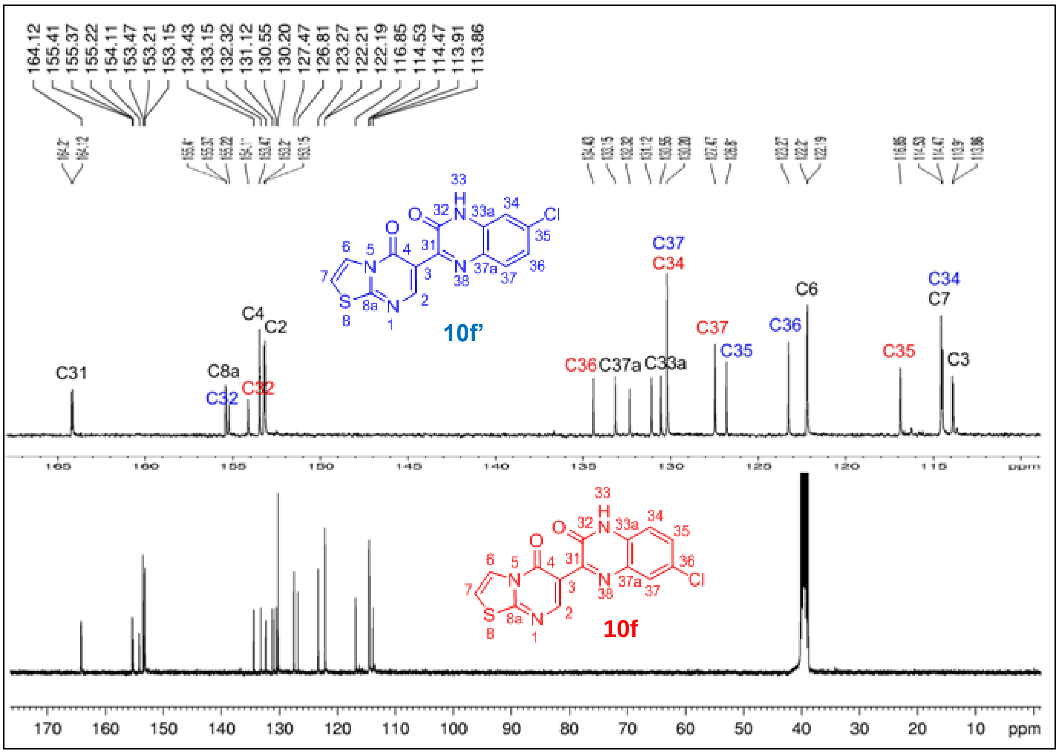

^{13}C NMR spectrum of compound **10f** and **10f'** in DMSO-d_6 at 25 °C.

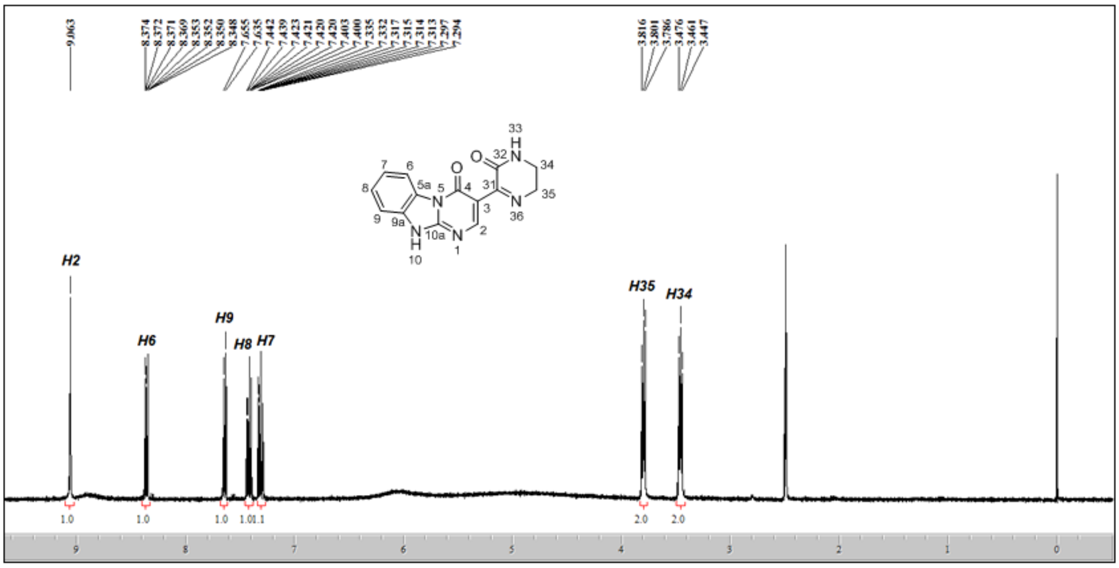

^{1}H NMR spectrum of compound **11a** in DMSO-d_6 at 25 °C.

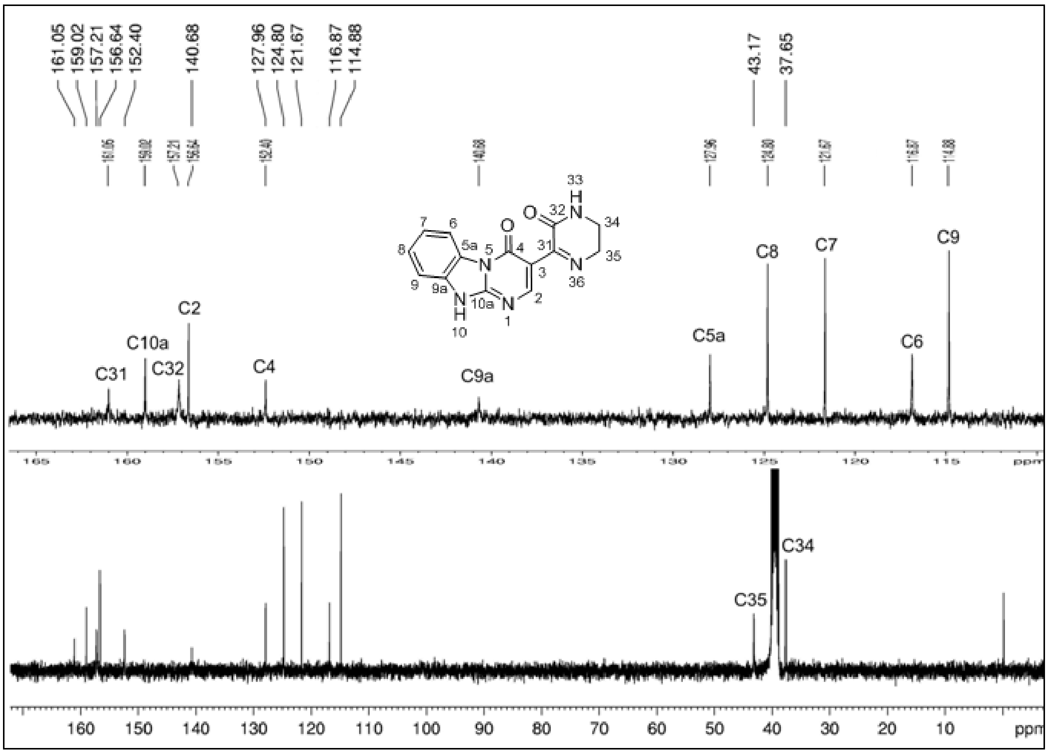

^{13}C NMR spectrum of compound **11a** in DMSO-d_6 at 25 °C.

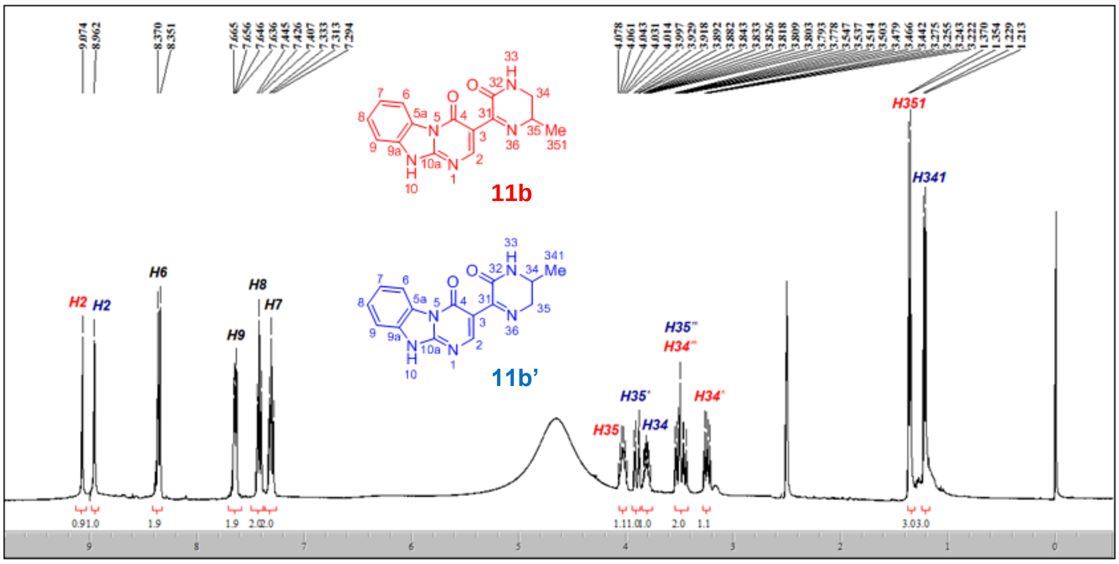

^{1}H NMR spectrum of compound **11b** and **11b'** in DMSO-d_6 at 25 °C.

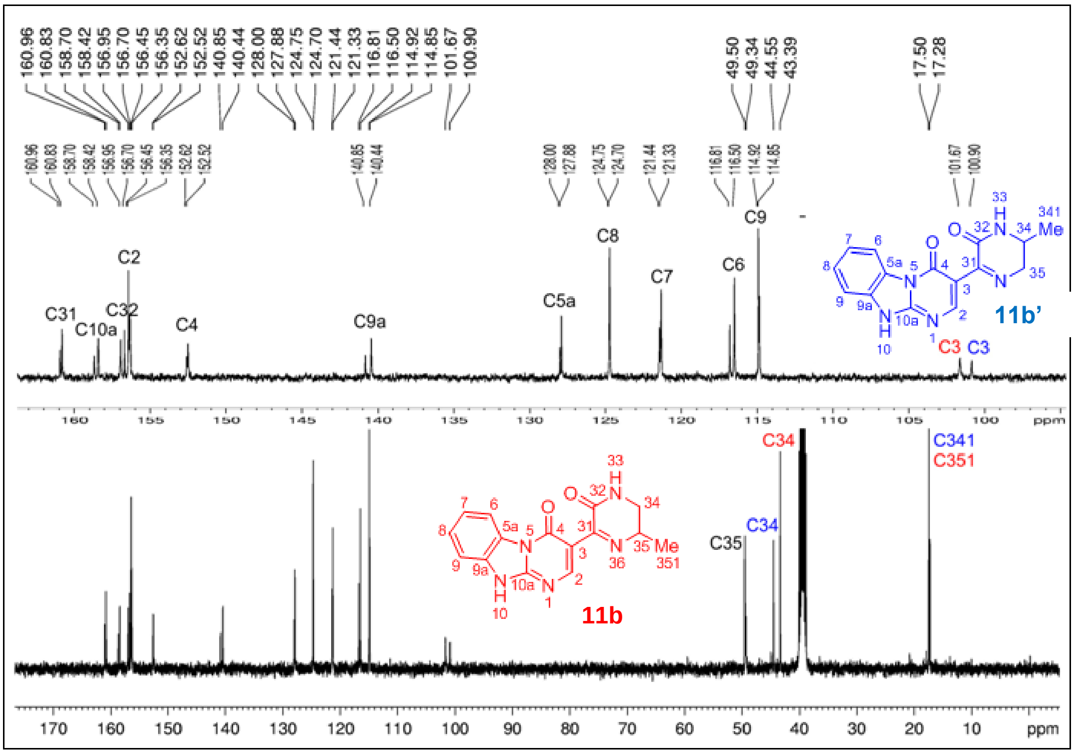

^{13}C NMR spectrum of compound **11b** and **11b'** in DMSO-d_6 at 25 °C.

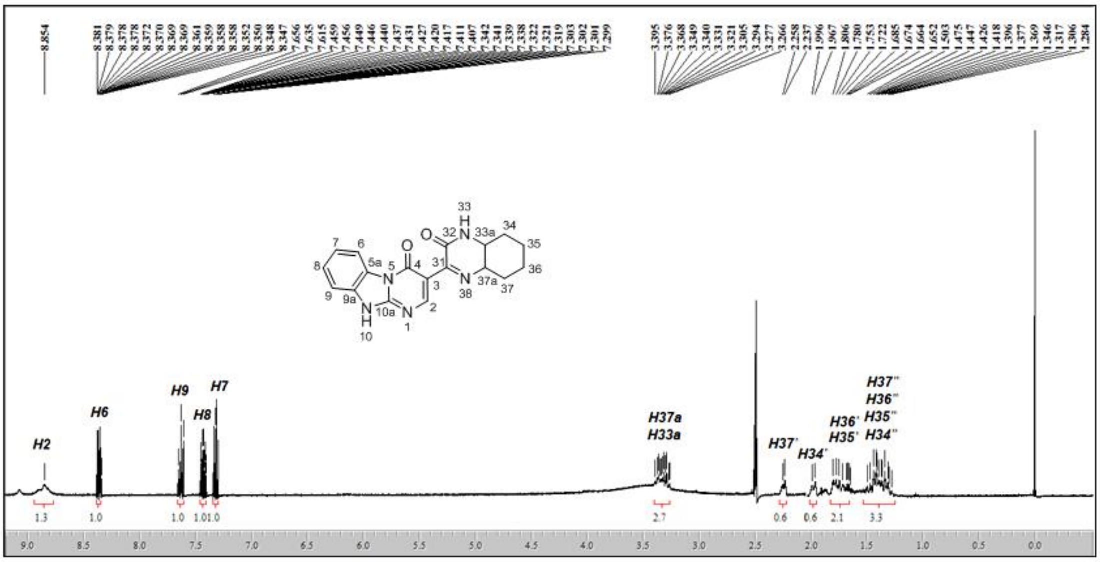

^{1}H NMR spectrum of compound **11c** in DMSO-d_6 at 25 °C.

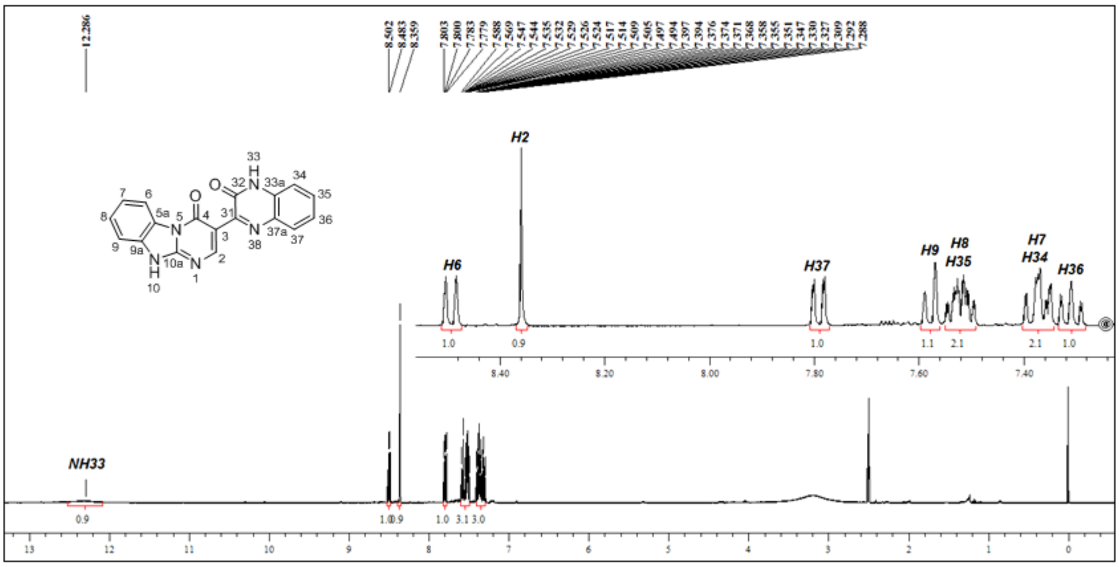

^{1}H NMR spectrum of compound **11d** in DMSO-d_6 at 25 °C.

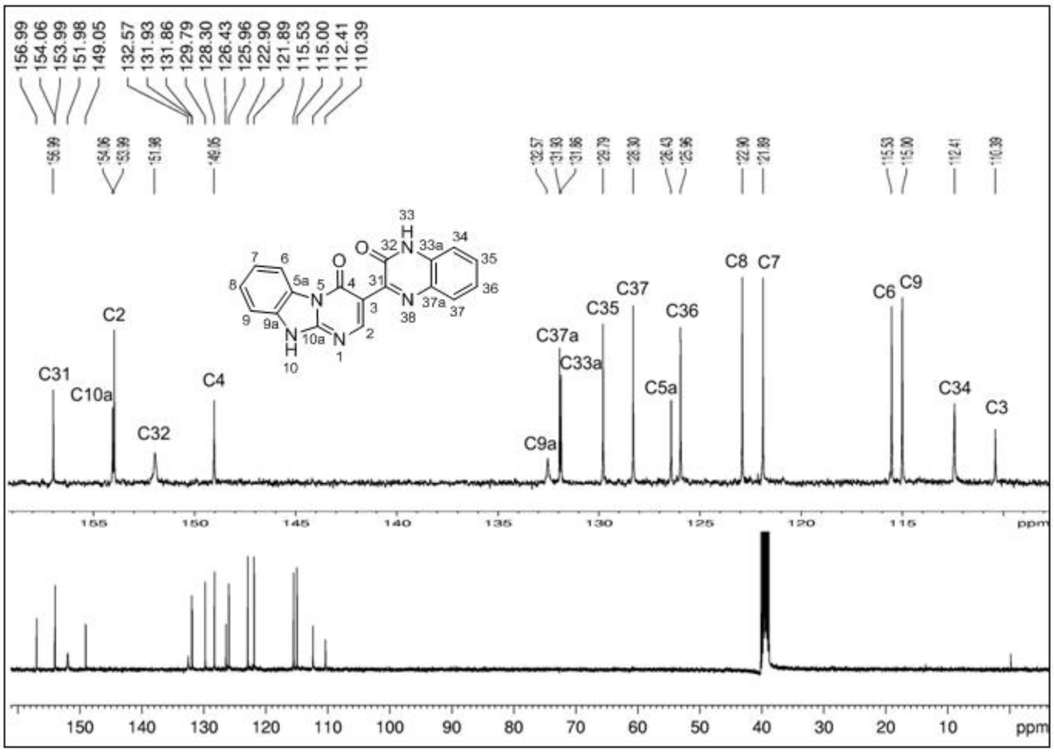

^{13}C NMR spectrum of compound **11d** in DMSO-d_6 at 25 °C.

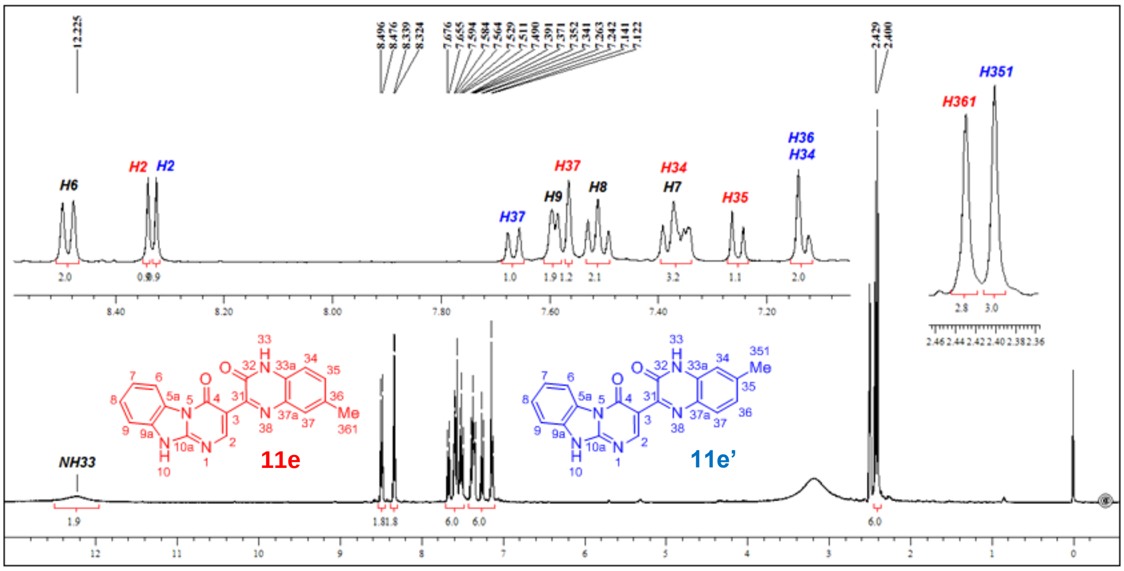

^{1}H NMR spectrum of compound **11e** and **11e’** in DMSO-d_6 at 25 °C.

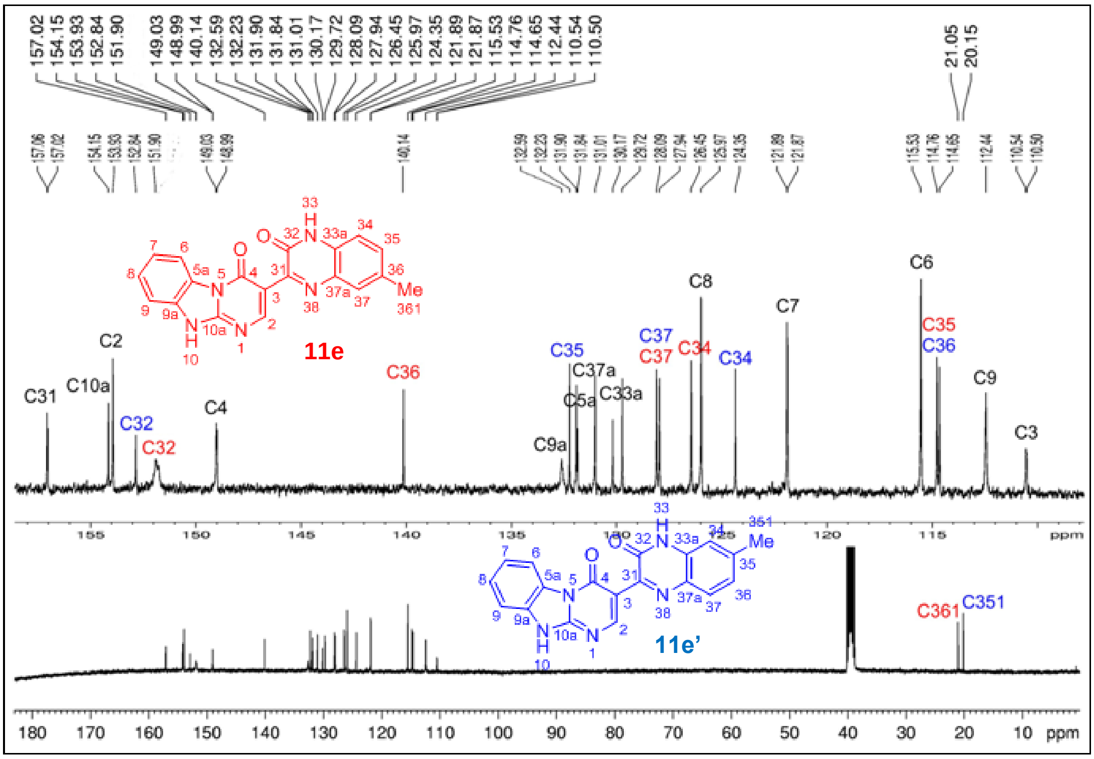

^{13}C NMR spectrum of compound **11e** and **11e'** in DMSO-d_6 at 25 °C.

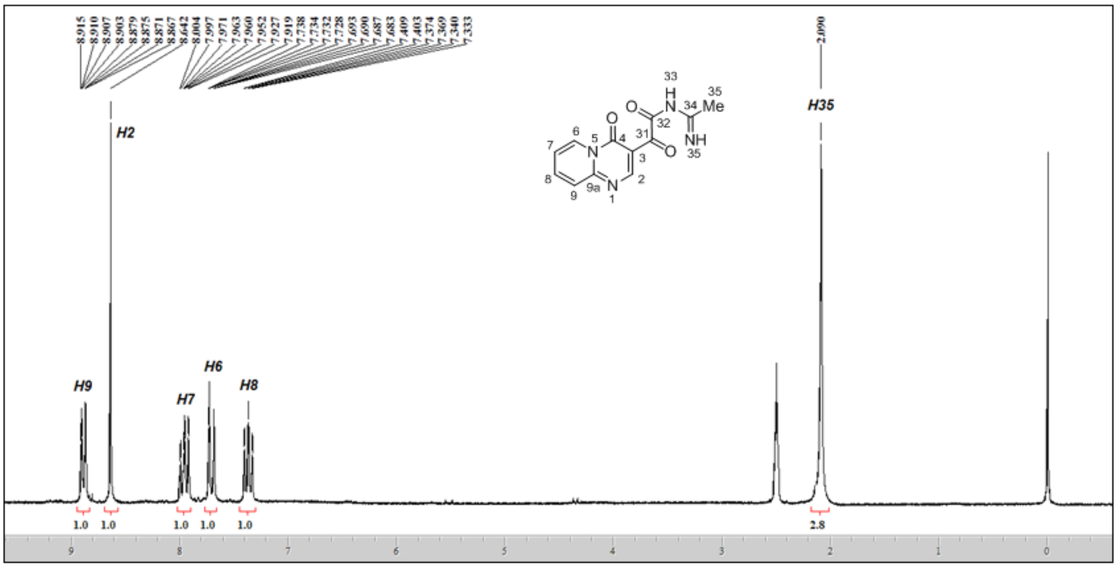

^{1}H NMR spectrum of compound **12g** in DMSO-d_6 at 25 °C.

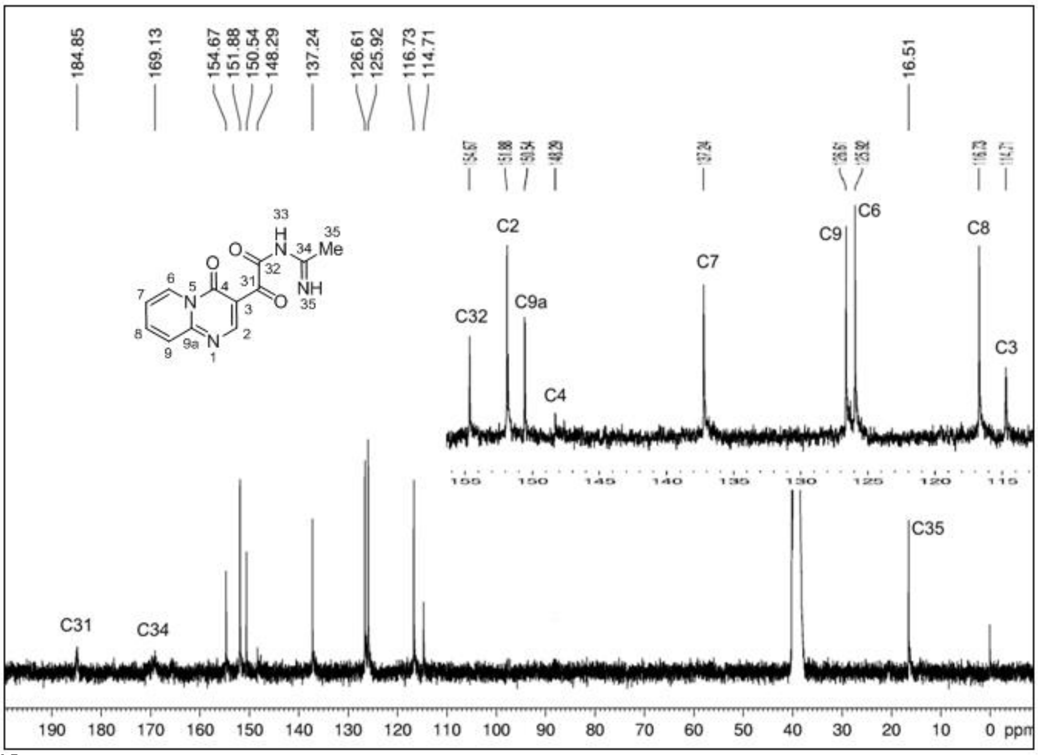

^{13}C NMR spectrum of compound **12g** in DMSO-d_6 at 25 °C.

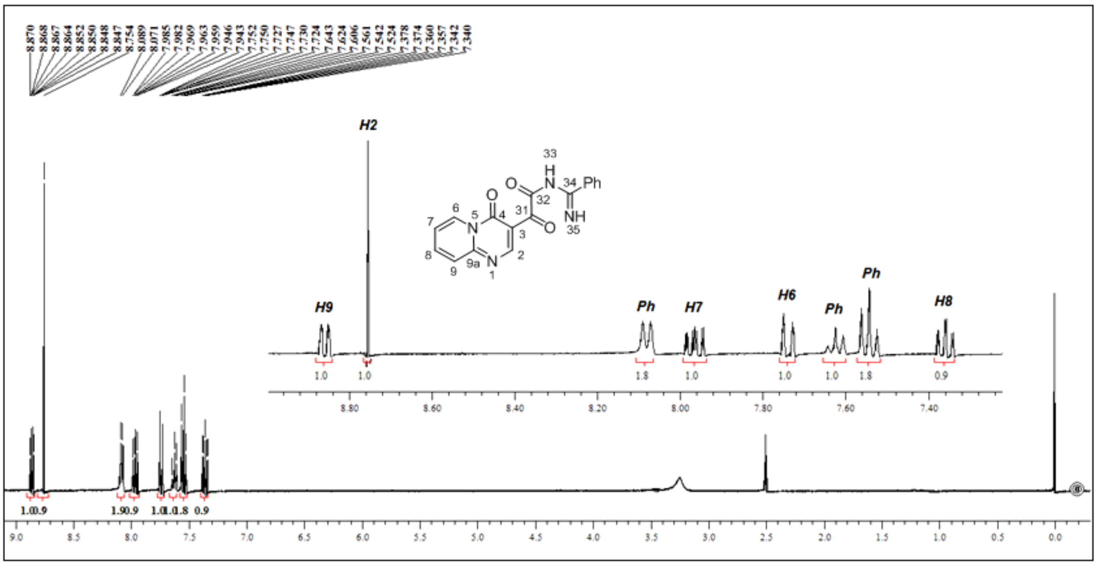

^{1}H NMR spectrum of compound **12h** in DMSO-d_6 at 25 °C.

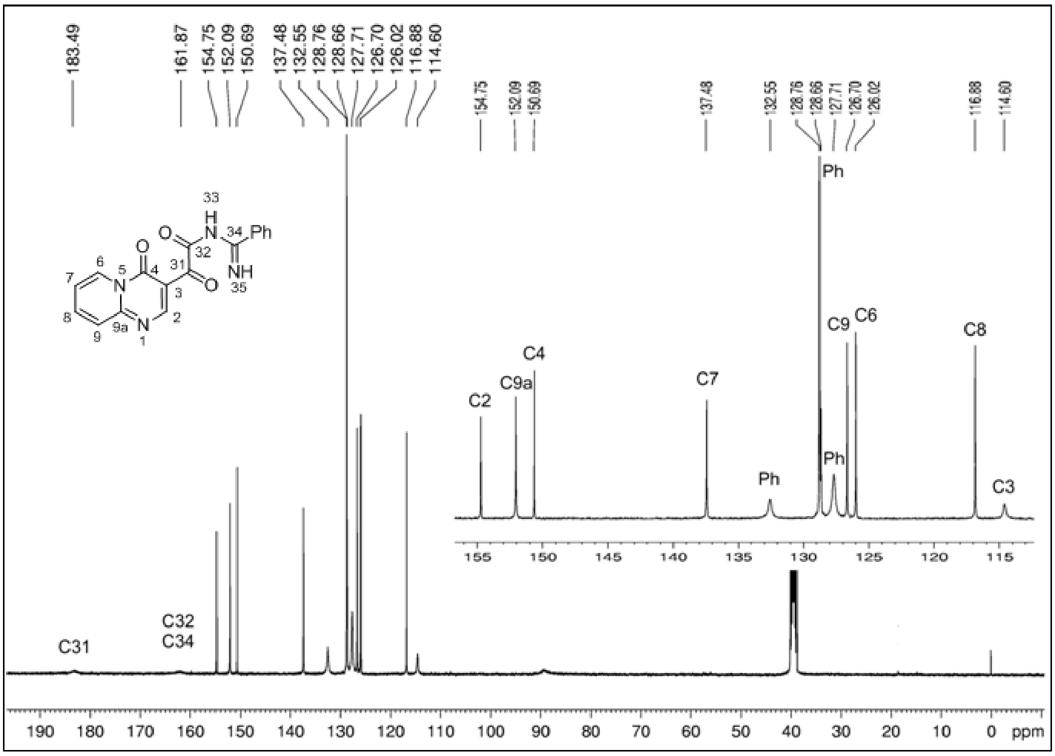

^{13}C NMR spectrum of compound **12h** in DMSO-d_6 at 25 °C.

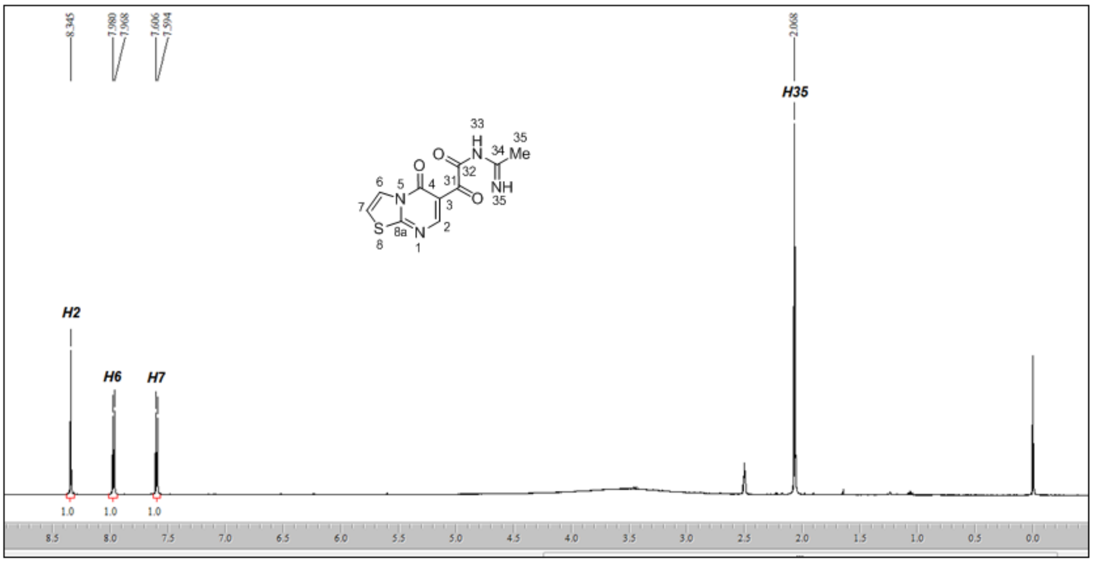

^{1}H NMR spectrum of compound **13g** in DMSO-d_6 at 25 °C.

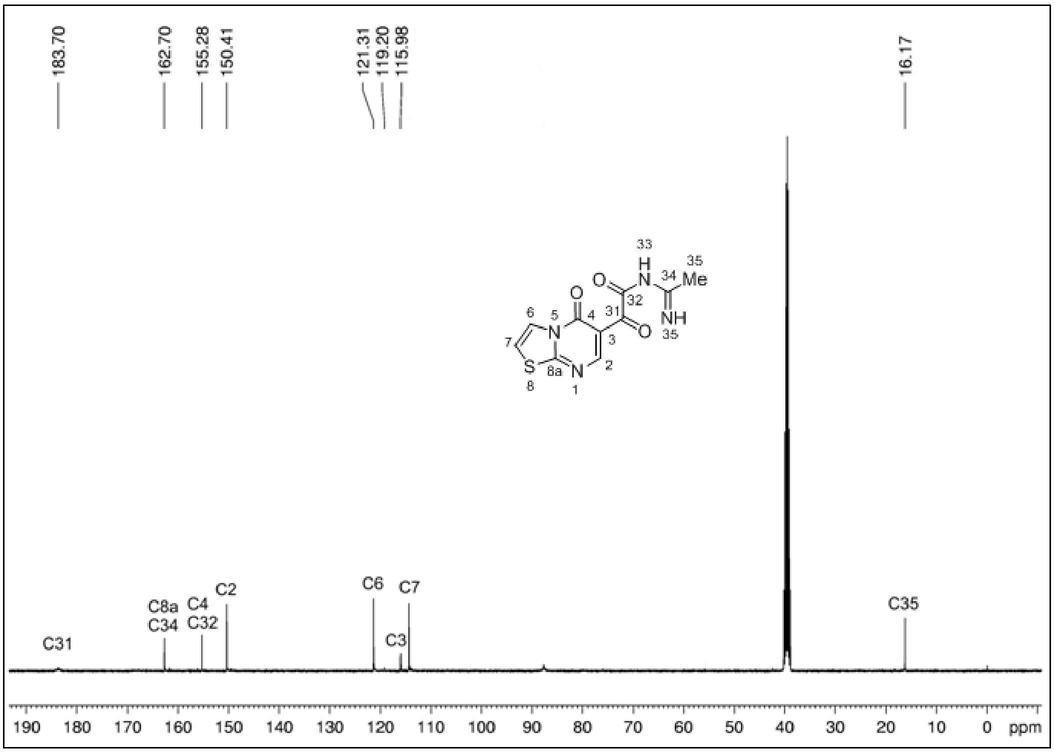

^{13}C NMR spectrum of compound **13g** in DMSO-d_6 at 25 °C.

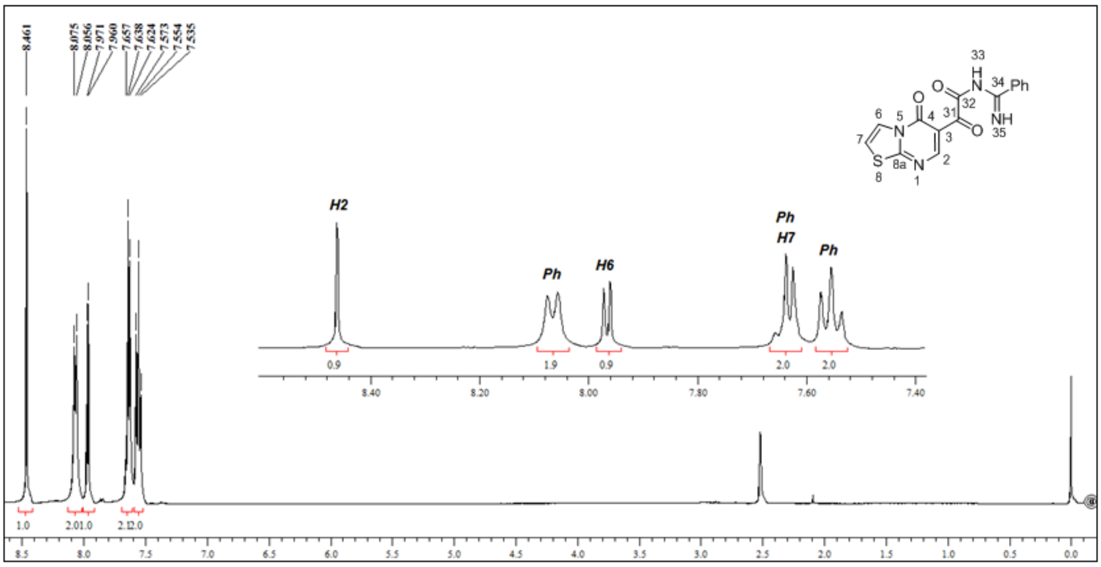

^{1}H NMR spectrum of compound **13h** in DMSO-d_6 at 25 °C.

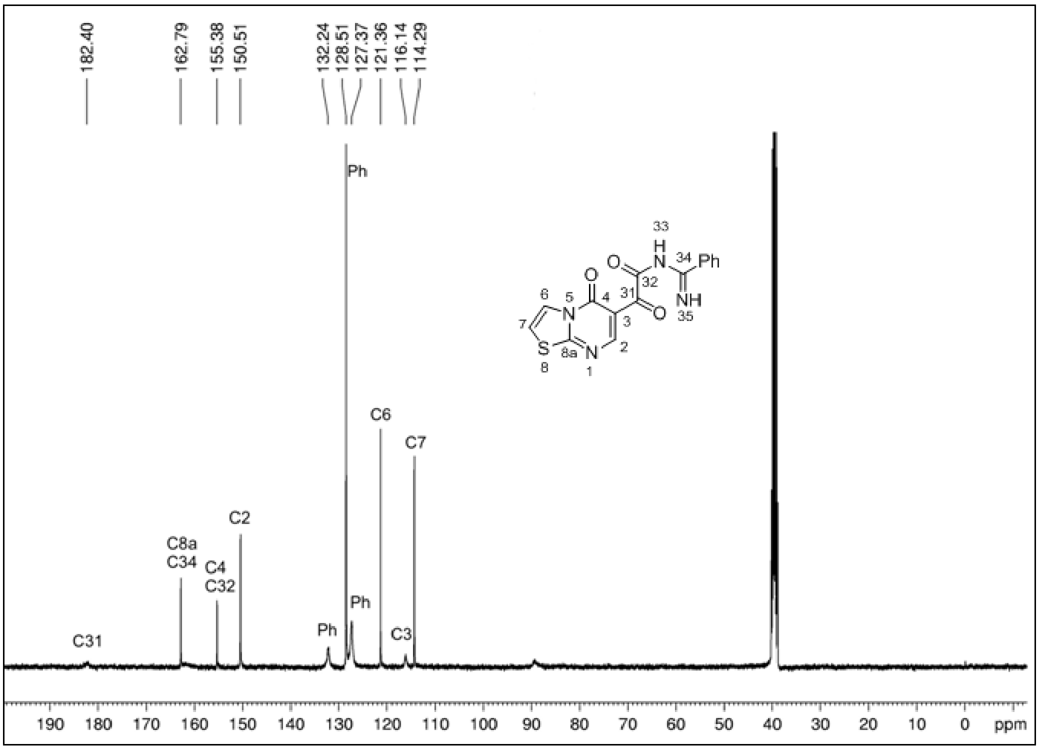

^{13}C NMR spectrum of compound **13h** in DMSO-d_6 at 25 °C.

5. Mass spectra

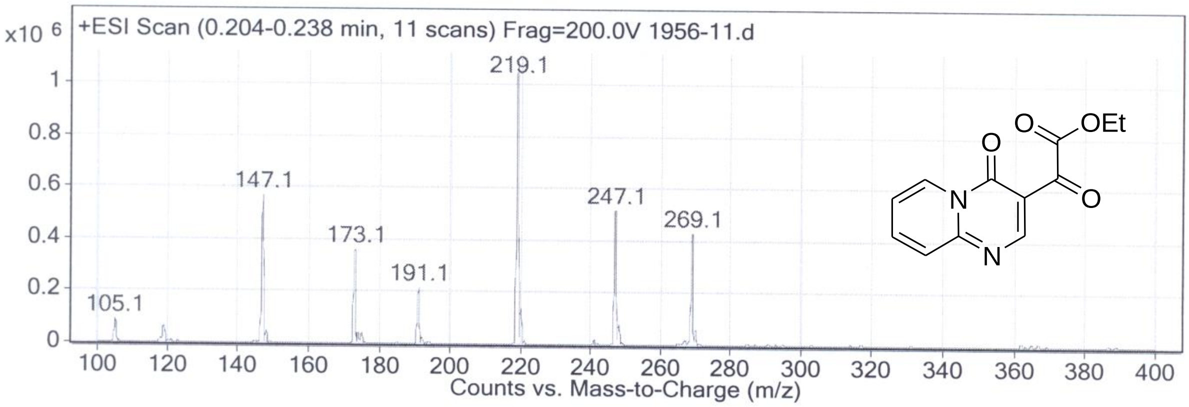

Mass spectrum of compound **5**.

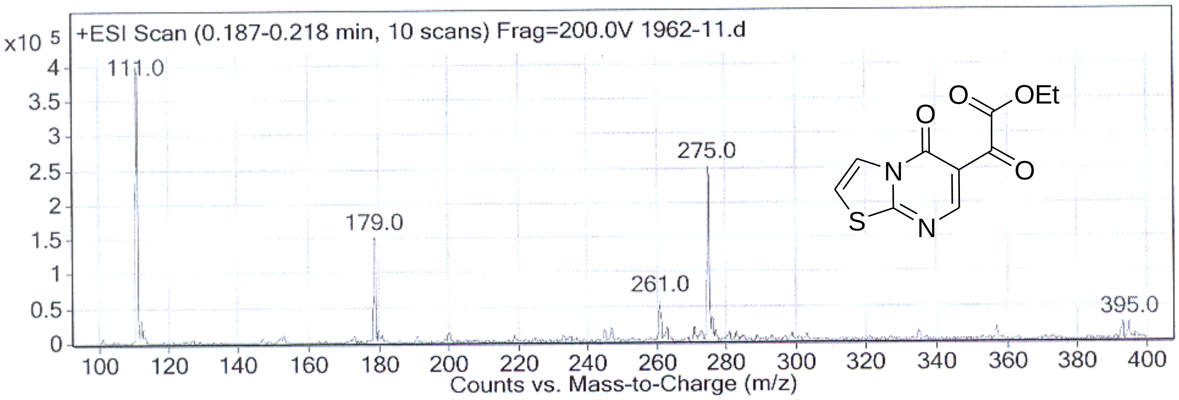

Mass spectrum of compound **6**.

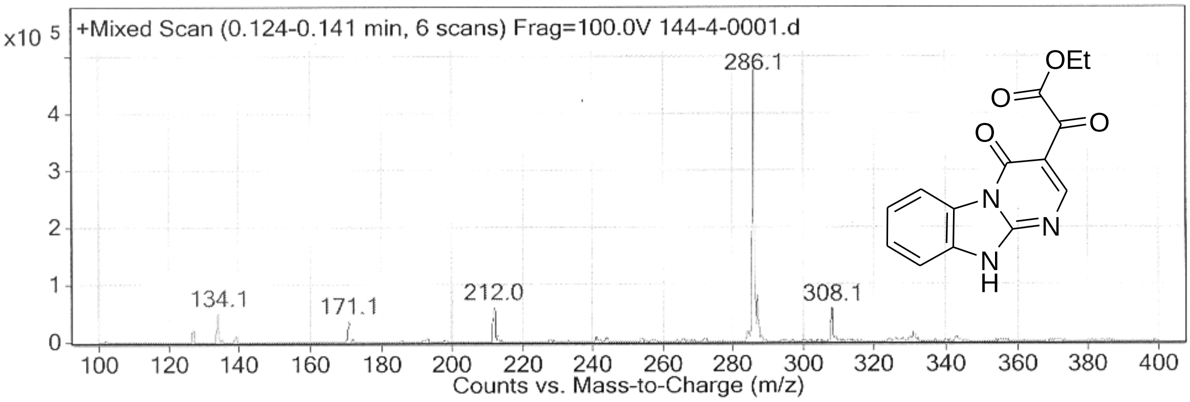

Mass spectrum of compound **7**.

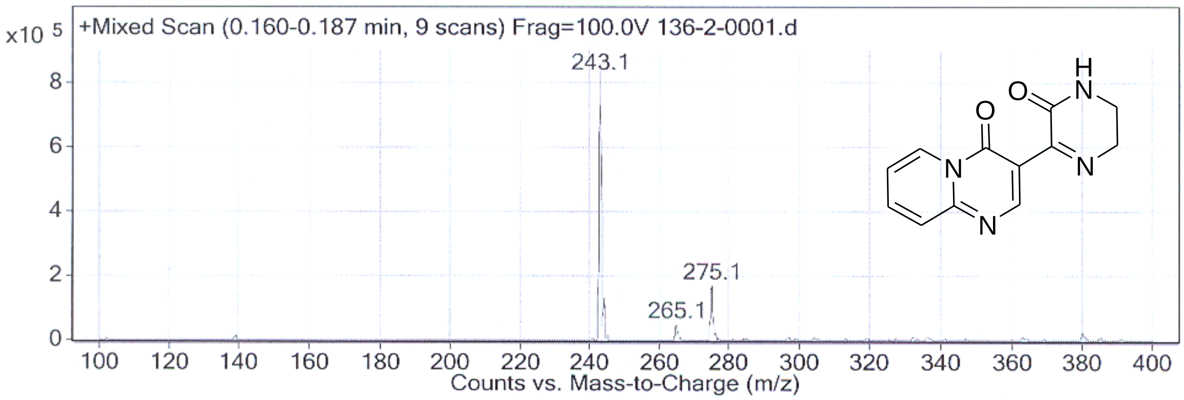

Mass spectrum of compound **9a**.

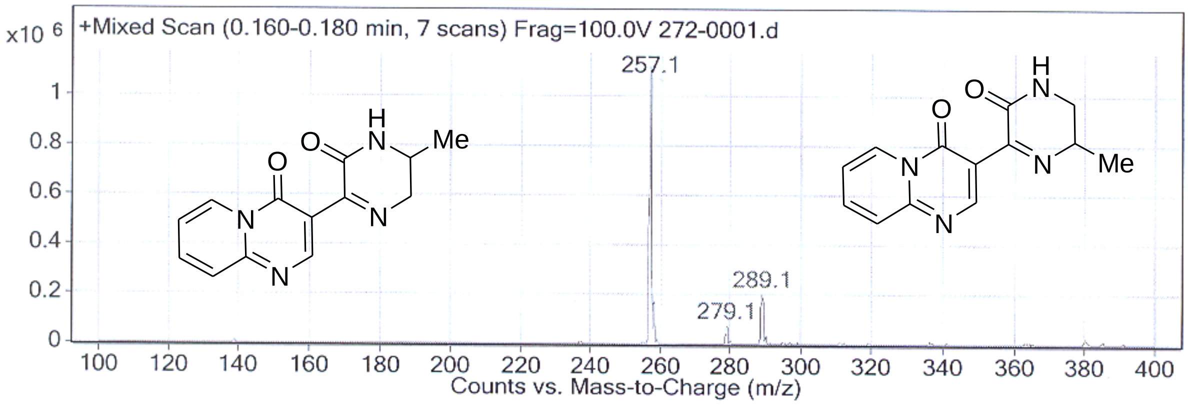

Mass spectrum of compounds **9b** and **9b'**.

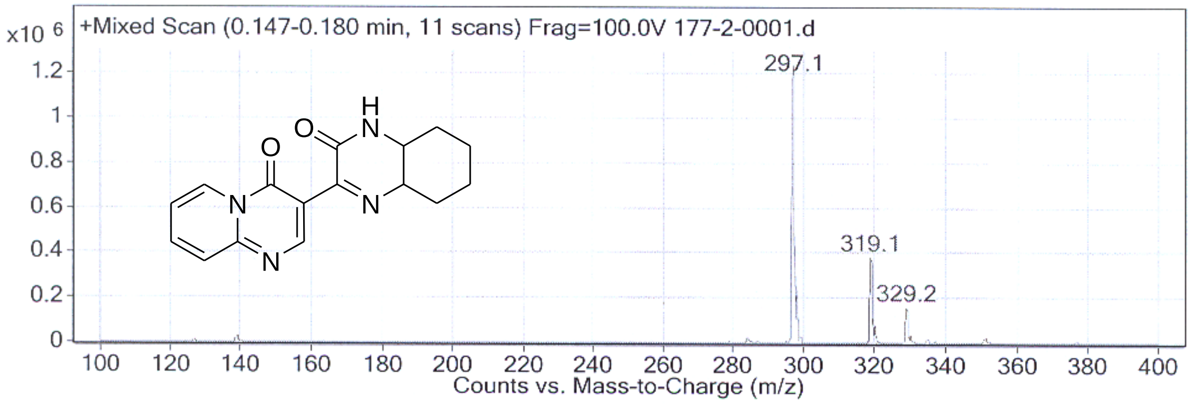

Mass spectrum of compound **9c**.

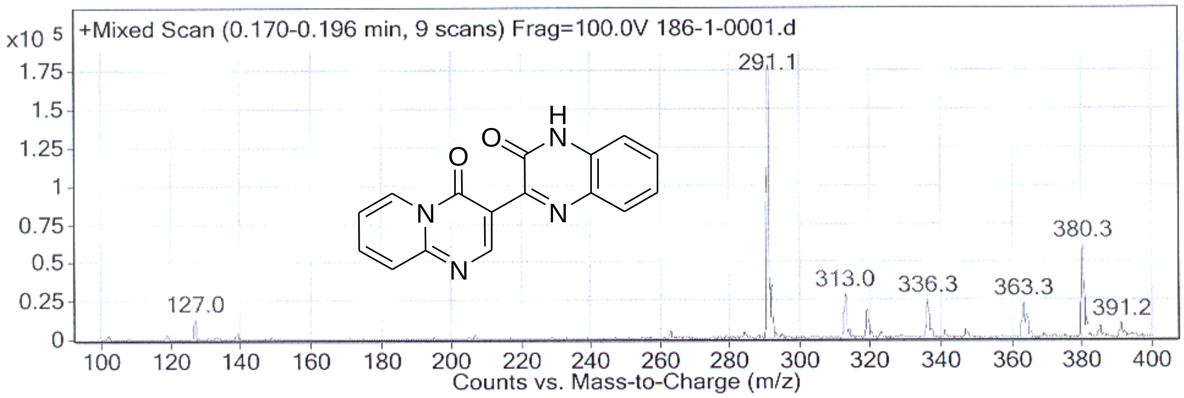

Mass spectrum of compound **9d**.

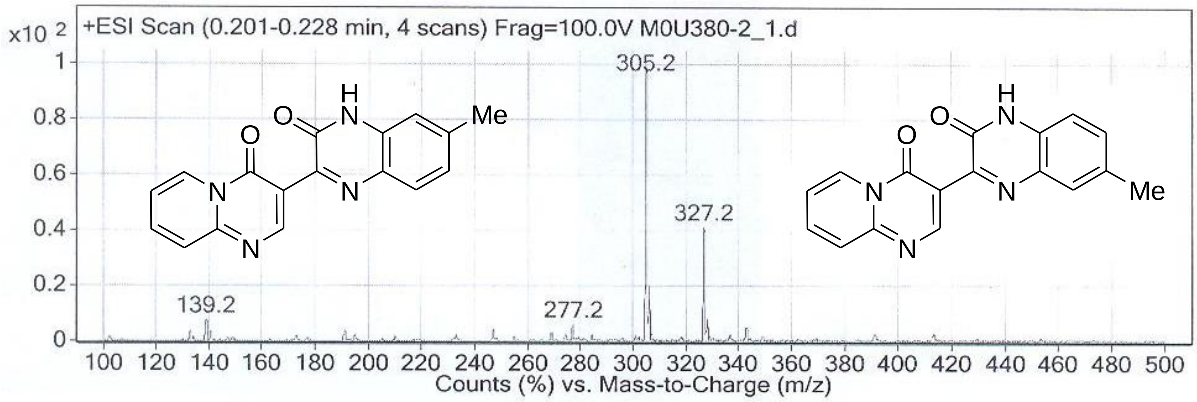

Mass spectrum of compounds **9e** and **9e’**.

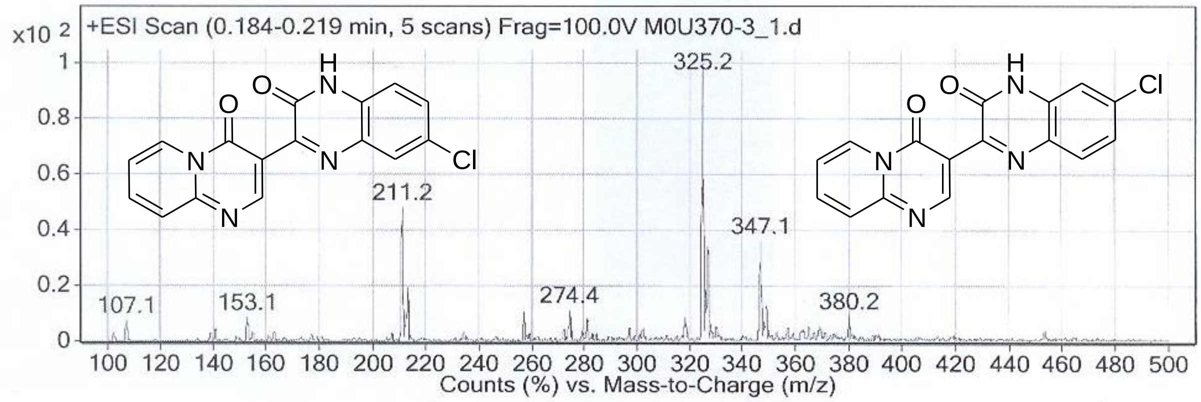

Mass spectrum of compounds **9f** and **9f’**.

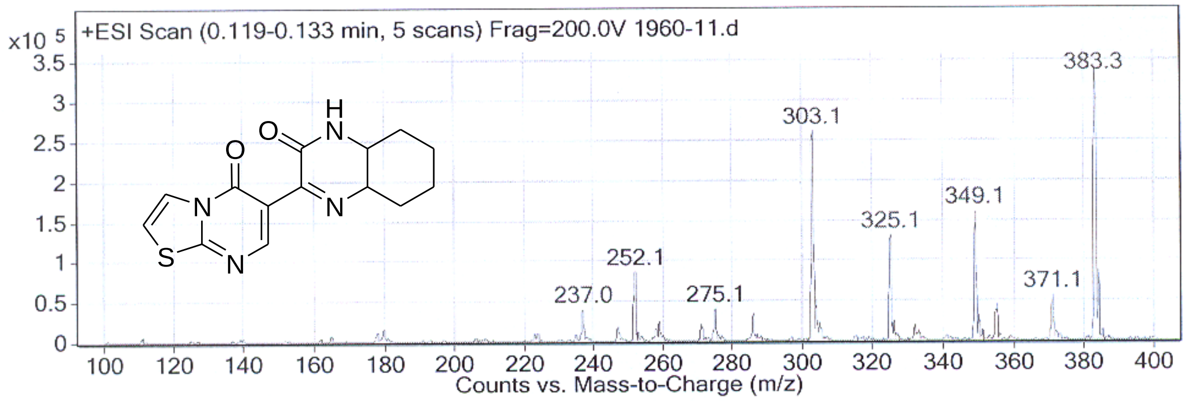

Mass spectrum of compound **10c**.

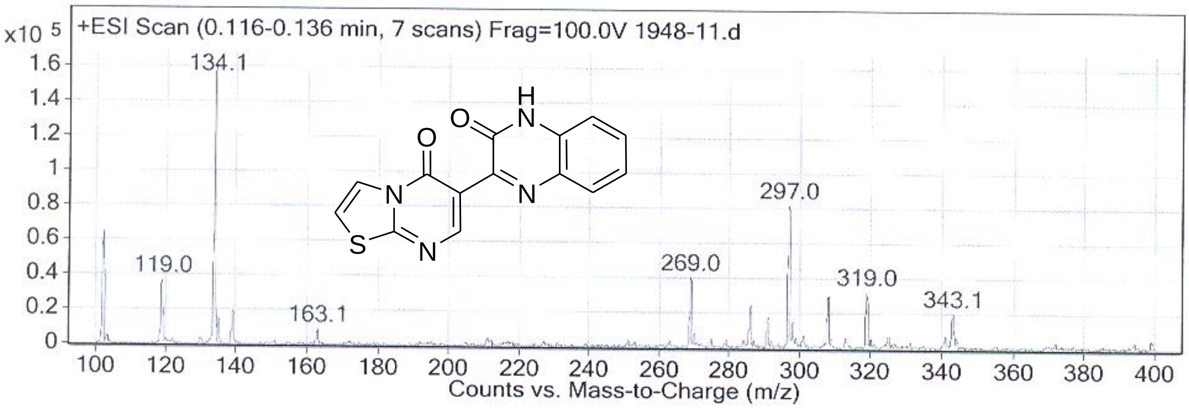

Mass spectrum of compound **10d**.

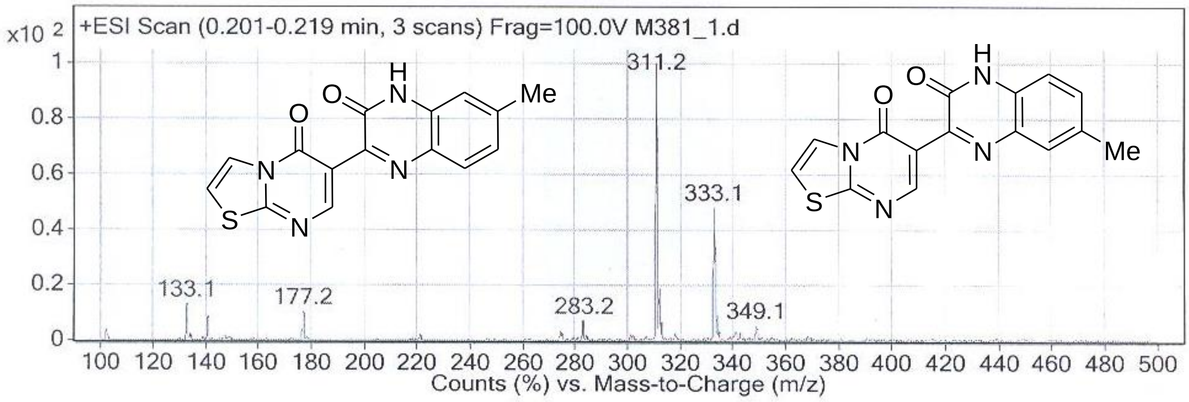

Mass spectrum of compounds **10e** and **10e’**.

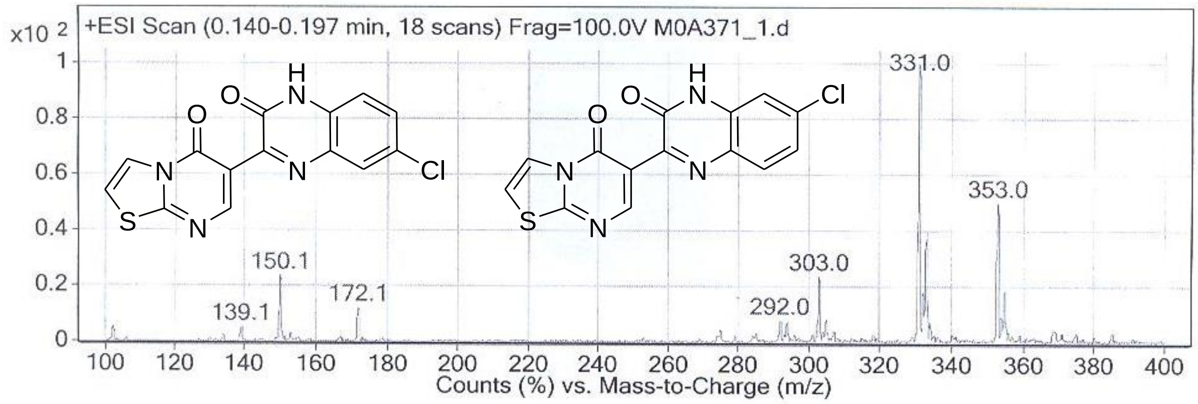

Mass spectrum of compounds **10f** and **10f'**.

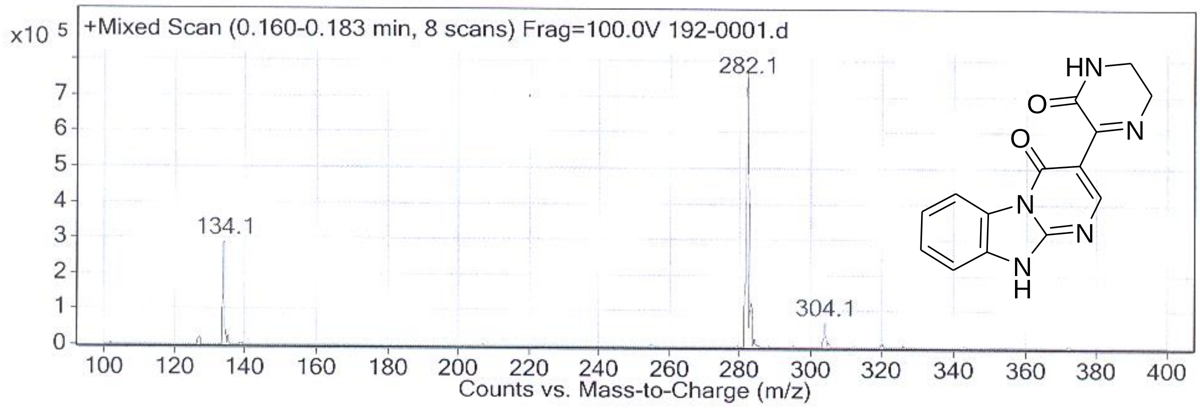

Mass spectrum of compound **11a**.

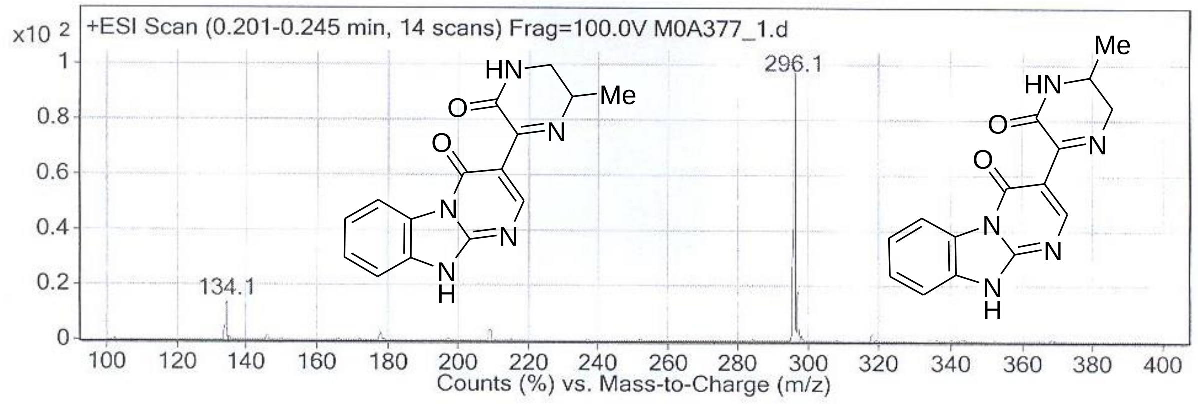

Mass spectrum of compounds **11b** and **11b'**.

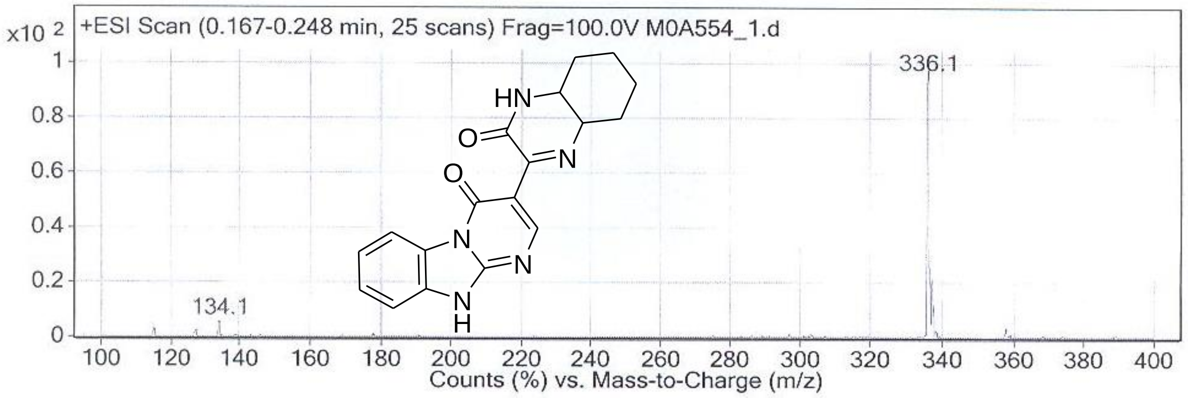

Mass spectrum of compound **11c**.

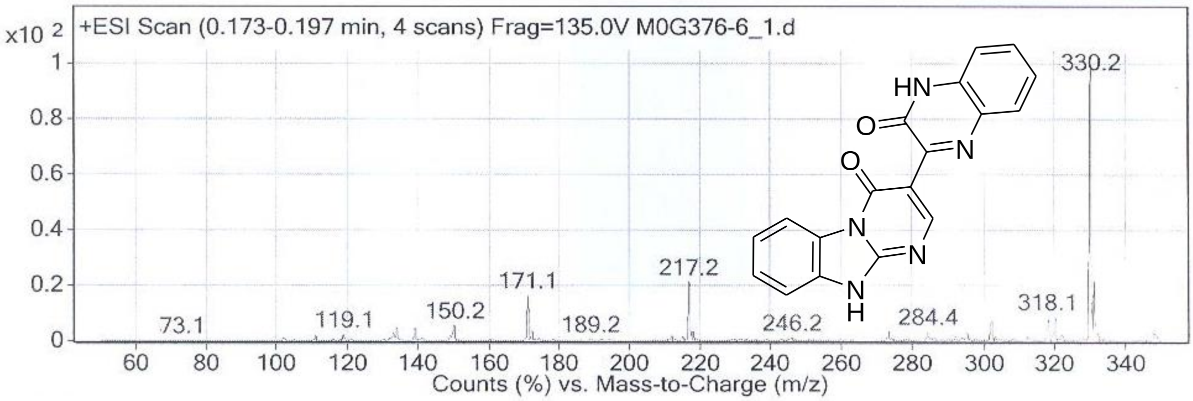

Mass spectrum of compound **11d**.

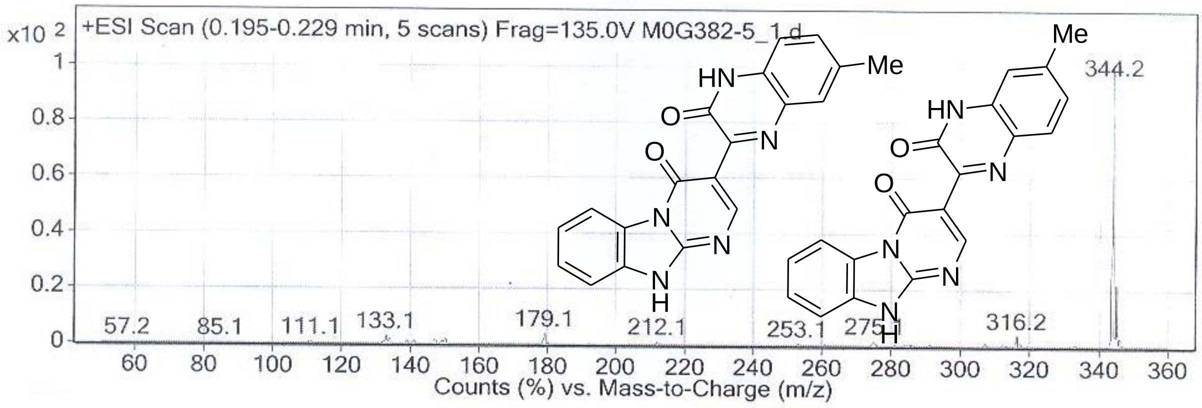

Mass spectrum of compounds **11e** and **11e’**.

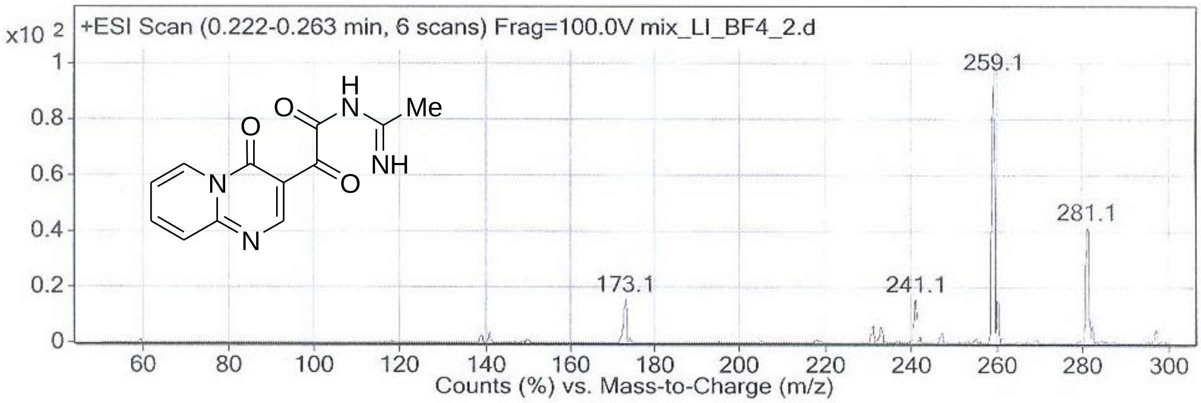

Mass spectrum of compound **12g**.

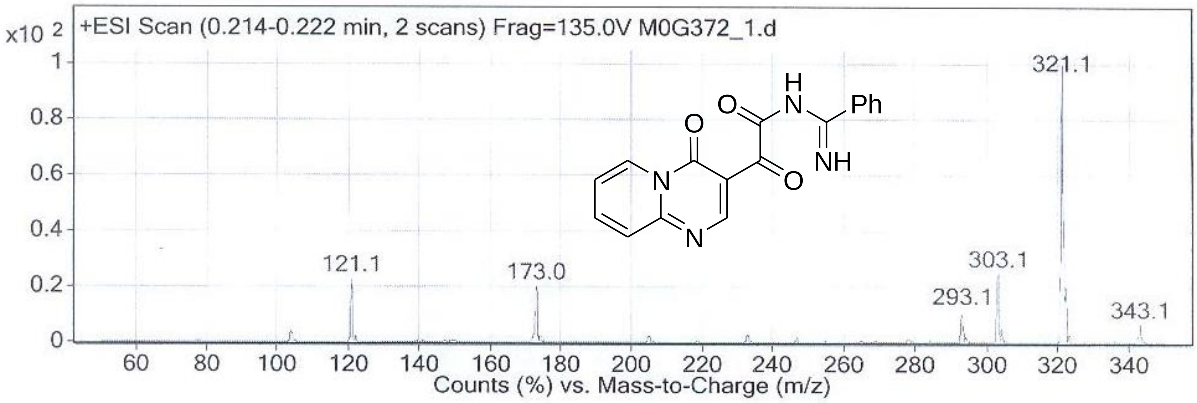

Mass spectrum of compound **12h**.

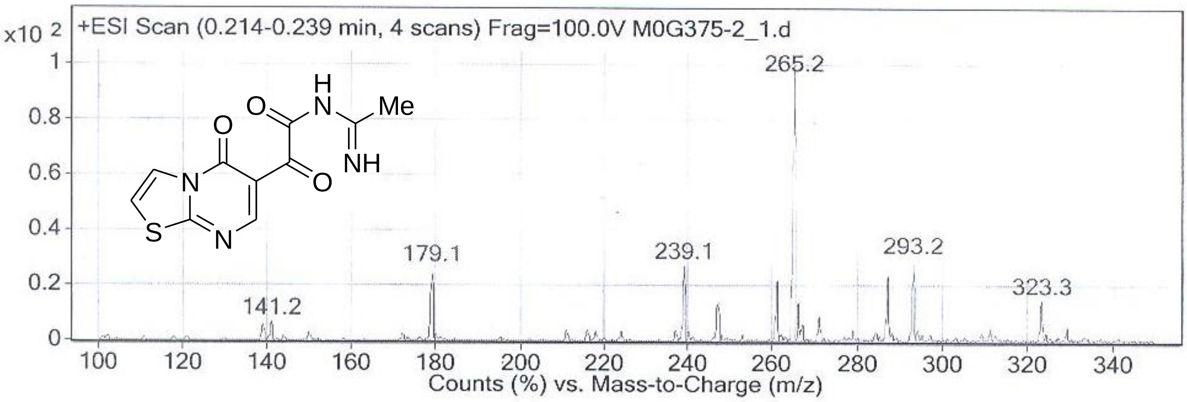

Mass spectrum of compound **13g**.

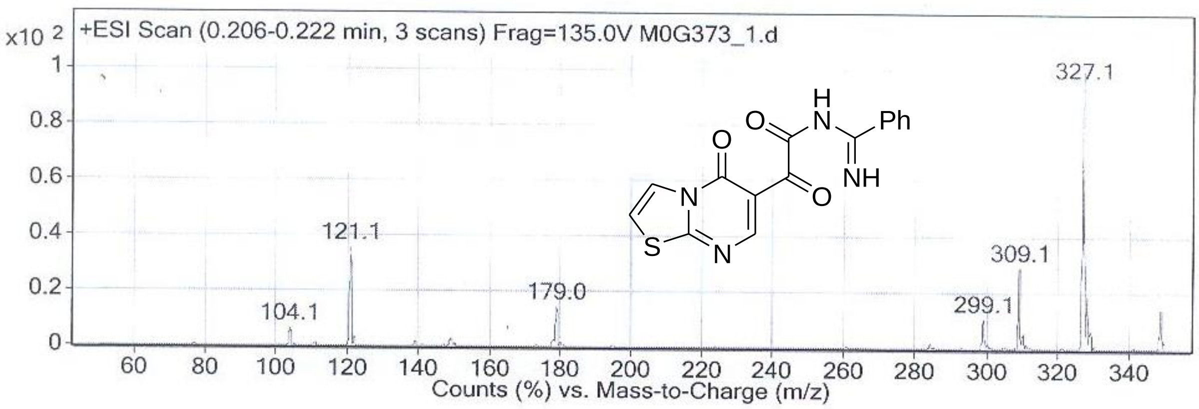

Mass spectrum of compound **13h**.

Table S3: Atomic coordinates used in theoretical calculations for all compounds

Compound	Atoms	Coordinates		
	C	1.74751000	-1.38630400	0.01450200
	C	-4.42378600	-0.85668800	0.29254800
	H	-3.99423100	-1.70006600	0.83258800
	H	-4.91093500	-0.20454800	1.01750700
	C	-5.36774100	-1.29956200	-0.80107300
	H	-4.85776400	-1.94441500	-1.51605300
	H	-6.19801800	-1.85739100	-0.36609800
	H	-5.77358900	-0.44212500	-1.33746700
	O	-0.62185900	-1.54873700	-0.19966700
	O	-1.66865500	2.14564500	-0.81348200
	O	-2.34302100	0.17940000	1.66962600
	C	1.14708900	1.58204900	-0.30948400
	H	2.09620800	1.21135300	-0.66823500
	N	1.17599300	2.87879500	-0.03221900
	C	2.31027000	3.68767700	-0.46438000
**1**	H	1.98938400	4.42659800	-1.20106600
	H	2.74294400	4.21249100	0.38798900
	H	3.06726800	3.05330700	-0.91774000
	C	0.14938000	3.58098500	0.72746100
	H	-0.32051700	2.89318300	1.42603500
	H	0.63263900	4.38227500	1.28419600
	H	-0.62229500	3.98973500	0.07674100
	Cl	1.63604800	-3.12933300	0.31182600
	Cl	2.66151400	-0.63811700	1.37572700
	Cl	2.65072400	-1.13171700	-1.53767000
	C	0.29446000	-0.75870100	-0.13722700
	C	0.09442000	0.68256600	-0.17995400
	C	-1.30926700	1.11874500	-0.26351500
	C	-2.37286100	0.29441500	0.46876700
	O	-3.34406000	-0.12096000	-0.33482100
Energy:	-2124.88662643 a.u.			

	C	1.19375700	1.16548000	-0.00347400
	C	-0.18543800	1.21432800	0.00520600
	C	-0.89867000	0.00289900	0.00761000
	C	1.04444100	-1.20642700	-0.00235300
	C	1.83779400	-0.07058600	-0.00507100
	H	1.76648900	2.08379400	-0.00687600
**2**	H	-0.71195400	2.15943900	0.01733200
	H	1.49787700	-2.19171900	-0.00686900
	H	2.91512500	-0.14866400	-0.01042800
	N	-0.28919800	-1.18548600	-0.00100600
	N	-2.27683900	-0.01700500	0.06559200
	H	-2.76785000	0.78584900	-0.28781200
	H	-2.68873400	-0.90542600	-0.16896000
Energy:	-303.76346788 a.u.			
	N	-0.15569900	1.34810400	-0.00203600
	C	1.20660700	1.15780500	-0.00392900
	H	1.85296800	2.02276600	-0.00908400
	C	1.64198600	-0.12340300	-0.00967900
	H	2.64886900	-0.50100300	-0.02413000
**3**	C	-0.77865700	0.21048600	0.00203700
	N	-2.14565100	0.08467700	0.07244600
	H	-2.63369200	0.94081700	-0.14175200
	H	-2.55043700	-0.73291900	-0.35220800
	S	0.27325800	-1.20177800	0.00648300
Energy:	-624.53087799 a.u.			
	C	-0.19924800	0.69855700	0.00053600
	C	-0.15939100	-0.71158700	0.00035200
	C	-1.35019000	-1.43130700	0.00375400
	C	-2.54581900	-0.72248900	0.00161800
	C	-2.56561700	0.67630900	-0.00161000
	C	-1.38536400	1.41426600	0.00162400
	H	-1.33438300	-2.51233100	0.00660900
	H	-3.48327100	-1.26234900	0.00201600
**4**	H	-3.51435200	1.19547300	-0.00531200
	H	-1.40255600	2.49613100	0.00283100
	C	1.87944200	-0.07297800	-0.00159200
	N	1.15618300	-1.15803200	0.00796400
	N	1.13611400	1.08528400	0.01398200
	H	1.49015300	2.01108900	-0.14733200
	N	3.25584800	-0.03373000	-0.08268900
	H	3.65949700	-0.94940500	0.04487000
	H	3.70500700	0.66211800	0.49343200
Energy:	-435.38775237 a.u.			
	N	1.91560400	-0.47054200	0.00356900
**5**	N	2.30879100	1.86180100	-0.29163400
	C	4.15105300	0.35470300	-0.21154800
	H	4.79240500	1.21184900	-0.34678700

	C	4.63400600	-0.91466500	-0.08605300
	H	5.69912300	-1.09749000	-0.11997100
	C	3.73735100	-1.99288800	0.08848000
	H	4.09270800	-3.00670000	0.19079200
	C	2.40313800	-1.74482700	0.12663200
	H	1.63875100	-2.49461700	0.25158400
	C	2.75891300	0.61532600	-0.16845400
	C	0.04542200	1.04926900	-0.04819300
	C	0.99407500	2.04534500	-0.23134900
	H	0.64114600	3.06524300	-0.32835300
	C	-4.15493400	-1.06721400	0.10625800
	H	-3.64525800	-1.86409500	0.64692900
	H	-4.82020000	-0.55741900	0.80267200
	C	-4.88760900	-1.57915100	-1.11185100
	H	-4.20286400	-2.07913800	-1.79638500
	H	-5.65130900	-2.29546800	-0.80624900
	H	-5.37582600	-0.76408600	-1.64553600
	O	-3.15542600	-0.11957200	-0.35035500
	O	-2.45603700	0.21528700	1.77497800
	O	-1.78844000	2.52443800	-0.28314300
	O	-0.22791400	-1.31186800	0.16340000
	C	-2.37496500	0.40744100	0.58966000
	C	-1.37995200	1.41913300	0.01134300
	C	0.45401900	-0.31300000	0.05652700
Energy:	-874.02013780 a.u.			
**6**	N	2.31722800	1.71485400	-0.25658700
	C	0.01384900	0.98429100	-0.05371200
	C	0.99315400	1.94649600	-0.21554700
	H	0.68297100	2.97916600	-0.31264800
	C	-4.26740500	-0.97556800	0.12871800
	H	-3.77881300	-1.78391400	0.67167100
	H	-4.90772600	-0.43675600	0.82659500
	C	-5.02872900	-1.47321200	-1.07755200
	H	-4.36758800	-2.00126600	-1.76416000
	H	-5.81219500	-2.16149000	-0.75800500
	H	-5.49548800	-0.64717400	-1.61352000
	O	-3.24068400	-0.06587200	-0.34653600
	O	-2.49831300	0.25475400	1.76680400
	O	-1.76951700	2.51499100	-0.33821400
	O	-0.34415700	-1.38214700	0.15015000
	C	-2.42880700	0.43588800	0.57894000
	C	-1.40152400	1.40419700	-0.01665900
	C	0.37042700	-0.40544000	0.05621100
	S	4.31010500	-0.11679300	-0.14534600
	C	3.75068300	-1.75885600	0.05772300
	H	4.44945400	-2.57417300	0.11600900
	C	2.41373800	-1.83028600	0.12549100
	H	1.78555200	-2.69532700	0.24711900
	C	2.66355900	0.46073000	-0.13299500

	N	1.80132100	-0.58823000	0.02103300
Energy:	-1194.79226400 a.u.			
	N	1.17640600	2.73821000	-0.26405500
	C	-0.82200200	1.37875000	-0.02680500
	C	-0.15594600	2.58135900	-0.18519300
	H	-0.75009300	3.48434300	-0.24670200
	C	-4.37411200	-1.69929700	0.06995400
	H	-3.67967800	-2.37064900	0.57417800
	H	-5.13093900	-1.39123100	0.79089800
	C	-4.98252800	-2.32542700	-1.16340200
	H	-4.20893100	-2.61824600	-1.87281200
	H	-5.54666900	-3.21598700	-0.88332000
	H	-5.66093700	-1.63150400	-1.65918700
	O	-3.63854000	-0.52380100	-0.35483600
	O	-3.03243200	-0.09228200	1.78168000
	O	-2.96534400	2.37046400	-0.19587700
	O	-0.51332500	-0.99774500	0.12493300
	C	-3.00437900	0.14904200	0.60298000
**7**	C	-2.29209000	1.39054300	0.05587800
	C	-0.08716500	0.13758700	0.03885900
	N	1.32310000	0.36111100	-0.03263100
	N	3.20308800	1.49352000	-0.22656500
	H	3.81456400	2.28465900	-0.33006500
	C	2.37290500	-1.95546900	0.14676700
	H	1.44864200	-2.50130700	0.23967900
	C	3.61507100	-2.58096800	0.15559900
	H	3.66122200	-3.65582900	0.25917000
	C	4.80186500	-1.85400900	0.03419900
	H	5.74791500	-2.37707000	0.04573800
	C	4.79532800	-0.47035200	-0.10099700
	H	5.71431000	0.09089100	-0.19404000
	C	2.36730100	-0.57545600	0.01131700
	C	3.55890500	0.15501700	-0.11049400
	C	1.85177200	1.61850600	-0.17999100
Energy:	-1005.65892941 a.u.			
	C	0.68060200	0.60234500	-0.30308500
	H	0.59517400	0.58540200	-1.39268100
	H	1.17665300	1.54628500	-0.03387400
	C	-0.72350100	0.57756000	0.30723200
	H	-0.63019300	0.59574300	1.39848800
	H	-1.25366800	1.49188300	0.03188600
**8a**	N	-1.54668600	-0.57235900	-0.05857600
	H	-1.79945200	-0.51774900	-1.03900300
	H	-0.98454500	-1.41106900	0.04184800
	N	1.42797000	-0.59293800	0.10508900
	H	2.27829600	-0.69250300	-0.43425700
	H	1.70613700	-0.52035000	1.07711600
Energy:	-190.59977801 a.u.			

	C	-0.47795900	0.03530600	0.32070800
	H	-0.35442100	0.03888800	1.41752400
	C	0.74025200	-0.67880900	-0.26393700
	H	0.72070400	-1.73153100	0.04662600
	H	0.65681400	-0.65326300	-1.35236900
	C	-1.76780800	-0.70990700	-0.00483800
	H	-1.75242000	-1.72290600	0.40175300
**8b**	H	-2.63154900	-0.19455800	0.41905900
	H	-1.91061900	-0.76501400	-1.08512000
	N	-0.52734400	1.38738300	-0.23732100
	H	-1.20356800	1.95745200	0.25581100
	H	0.38347000	1.81812000	-0.12133500
	N	1.97683500	0.01895300	0.10930300
	H	2.22692300	-0.18717000	1.06925900
	H	2.75131700	-0.28391200	-0.46669300
Energy:	-229.93150832 a.u.			

	C	0.50348700	-0.70418700	-0.00247600
	C	0.50348300	0.70420400	0.00248800
	C	-0.71001700	1.38375100	0.02319000
	C	-1.91811800	0.69353800	0.01807900
	C	-1.91811600	-0.69353700	-0.01805100
	C	-0.71000900	-1.38374100	-0.02325400
	H	-0.70535000	2.46742700	0.03246000
**8d**	H	-2.85008500	1.24189100	0.03136400
	H	-2.85007900	-1.24189800	-0.03125800
	H	-0.70534800	-2.46741700	-0.03256900
	N	1.74466200	-1.36522900	-0.04429500
	H	2.42006900	-0.96491200	0.59461900
	H	1.67046200	-2.36036700	0.10475000
	N	1.74472500	1.36520900	0.04434600
	H	2.41993600	0.96492900	-0.59480400
	H	1.67042400	2.36031900	-0.10477300
Energy:	-343.09204011 a.u.			

	C	0.71565600	0.76516400	-0.00028800
	C	1.15146700	-0.57414600	0.00108100
	C	0.20149000	-1.58577200	-0.02502600
	C	-1.16041600	-1.29446500	-0.03153700
	C	-1.60498200	0.02231900	-0.00010300
	C	-0.64676200	1.03865200	0.00937100
	H	0.53070100	-2.61841100	-0.03204300
**8e**	H	-1.87737200	-2.10511200	-0.05049600
	H	-0.96903800	2.07474100	0.01166000
	N	1.69017100	1.77730000	0.04461900
	H	2.47249200	1.59097800	-0.56984000
	H	1.31785200	2.69862800	-0.12948100
	N	2.53871600	-0.82124900	-0.03399900
	H	3.04887800	-0.25326500	0.63110800

	H	2.76825000	-1.79502100	0.09844700
	C	-3.07430300	0.35714100	0.02450500
	H	-3.37043800	0.78720800	0.98479000
	H	-3.33291400	1.08574700	-0.74673100
	H	-3.68350600	-0.53120500	-0.13977900
Energy:	-382.42245660 a.u.			

	C	1.04437100	0.77471800	0.00008600
	C	1.53976100	-0.54471200	0.00065500
	C	0.64030200	-1.60328700	-0.02506000
	C	-0.73511500	-1.38900600	-0.03025400
	C	-1.20364700	-0.08781800	0.00108400
	C	-0.32973800	0.99126400	0.01114200
	H	1.01844800	-2.61833700	-0.02967000
**8f**	H	-1.42346000	-2.22024300	-0.04505200
	H	-0.71866500	2.00025400	0.01388300
	N	1.96806600	1.82876800	0.04282700
	H	2.76274100	1.67853600	-0.56517900
	H	1.55830500	2.73521100	-0.12345800
	N	2.93388200	-0.72675800	-0.03293000
	H	3.42166500	-0.13235500	0.62536800
	H	3.21400800	-1.68782200	0.09252700
	Cl	-2.93425200	0.21669000	0.01272800
Energy:	-802.72421166 a.u.			

	C	-4.89311400	0.37446100	-0.01693000
	C	-3.95306500	1.26507600	-0.43718400
	C	-2.56554600	0.98048900	-0.32238700
	C	-3.16632000	-1.13244400	0.66638900
	C	-4.48918200	-0.85954900	0.54869000
	H	-5.94496300	0.60650200	-0.11208200
	H	-4.20762800	2.21887800	-0.87311500
	C	-0.81566100	-0.63155100	0.42709600
	H	-2.75618200	-2.03495200	1.08985100
	H	-5.21366100	-1.58366700	0.88860400
	C	0.09871400	0.31840900	-0.12840200
	C	-0.37743900	1.51137100	-0.62979300
**9b**	H	0.33891500	2.24243800	-0.98570500
	N	-2.21477000	-0.24149700	0.23796800
	N	-1.66536500	1.85847600	-0.73424900
	O	-0.57335800	-1.64955300	1.04126300
	C	1.54356200	0.03866400	-0.09714300
	C	2.01305200	-1.25426600	-0.64334200
	C	3.71125100	0.75162800	0.37212900
	C	4.22107900	-0.44873000	-0.00421300
	H	5.27187800	-0.69357100	0.04198100
	O	1.33805200	-2.08800500	-1.21874800
	N	2.35712800	0.96247000	0.32684900
	N	3.38312300	-1.41818900	-0.47473800
	H	3.75283200	-2.29371100	-0.81518700

	C	4.55799800	1.88111500	0.87674300
	H	4.43257900	2.76482000	0.24857700
	H	4.26149800	2.16195900	1.88860900
	H	5.61439300	1.61491200	0.88763700
Energy:	-871.17553776 a.u.			

	C	5.00077600	-0.05846200	-0.06226100
	C	4.14706600	-1.08731800	-0.31862900
	C	2.74015600	-0.92468200	-0.19907900
	C	3.14483800	1.35846800	0.45536600
	C	4.48580300	1.20020000	0.33393400
	H	6.06859600	-0.19922500	-0.15847700
	H	4.48765700	-2.06546000	-0.62167900
	C	0.85275900	0.60296500	0.37011300
	H	2.65442100	2.26947300	0.75793100
	H	5.14037600	2.03268000	0.54200700
	C	0.02698400	-0.50201300	-0.00949900
	C	0.61050700	-1.70307000	-0.35237000
	H	-0.03541400	-2.54433100	-0.57464100
	N	2.27913100	0.32678200	0.19166300
**9b’**	N	1.92512800	-1.93567700	-0.44999400
	O	0.52092100	1.67355300	0.83610700
	C	-1.43737300	-0.36105100	0.03791100
	C	-2.05284500	0.78287900	-0.66660000
	C	-3.50534900	-1.17852700	0.67922600
	C	-4.17623800	-0.11090300	0.16759900
	O	-1.48852800	1.58547500	-1.38564000
	N	-2.14790100	-1.28387000	0.62106700
	N	-3.43306800	0.84366900	-0.46699100
	H	-3.88680500	1.62673100	-0.91689700
	H	-4.03639100	-1.97406300	1.18111800
	C	-5.65380400	0.09435400	0.24883300
	H	-5.89295900	1.00656100	0.80068300
	H	-6.09475400	0.18555200	-0.74676900
	H	-6.12657200	-0.74350600	0.75550000
Energy:	-871.17788807 a.u.			

	C	5.91977700	-0.71993300	0.10512800
	C	4.93917900	-1.45902300	-0.48299300
	C	3.57395800	-1.07550000	-0.39308800
	C	4.27927100	0.81814400	0.91864900
	C	5.58138200	0.45220000	0.82425000
	H	6.95405400	-1.02536900	0.02601400
**9e**	H	5.14356800	-2.36016800	-1.04041200
	C	1.91544100	0.56035900	0.49699300
	H	3.91695600	1.68403100	1.44876300
	H	6.33919300	1.05654700	1.29880900
	C	0.96430700	-0.22054300	-0.23208300
	C	1.37284400	-1.36850600	-0.87735700
	H	0.62377100	-1.97797300	-1.36909000

	N	3.28789800	0.08099400	0.32158300
	N	2.63348600	-1.80636500	-0.96990100
	O	1.71684400	1.50046400	1.23795500
	C	-0.45356300	0.17654100	-0.22845400
	C	-0.77797100	1.57912300	-0.62963100
	O	0.02332100	2.38196800	-1.06756300
	N	-1.35767900	-0.71067700	0.03170800
	N	-2.11977600	1.88081700	-0.50958400
	H	-2.37314600	2.82291800	-0.77150900
	C	-2.69320900	-0.36104800	0.03845600
	C	-3.10315300	0.96346100	-0.20767100
	C	-4.45645500	1.29283500	-0.15350700
	H	-4.77277200	2.31148600	-0.33940300
	C	-5.38747700	0.30983600	0.13485900
	H	-6.43596400	0.57634400	0.17225800
	C	-5.00950300	-1.01830400	0.37851300
	C	-3.65949900	-1.33055100	0.32727700
	H	-3.31029400	-2.33748400	0.51543600
	C	-6.03795900	-2.07519800	0.69069200
	H	-6.01440400	-2.88144900	-0.04500000
	H	-5.85946400	-2.52614700	1.66873500
	H	-7.04453900	-1.65892900	0.69542400
Energy:	-1024.87725367 a.u.			

	C	-6.01798000	-0.41474800	-0.10121200
	C	-5.08802200	-1.27561700	0.39641600
	C	-3.69911700	-0.98636900	0.31559600
	C	-4.27768100	1.06914000	-0.80009100
	C	-5.60211900	0.79188500	-0.71502300
	H	-7.07110600	-0.64979400	-0.03068000
	H	-5.35214400	-2.20790400	0.87141800
	C	-1.93452500	0.60120600	-0.44968800
	H	-3.85844400	1.95229200	-1.25431900
	H	-6.31868900	1.49172300	-1.11689300
	C	-1.03502300	-0.31345100	0.18300800
	C	-1.52004200	-1.48423800	0.72588700
**9e’**	H	-0.81233800	-2.19094100	1.14313900
	N	-3.33652700	0.20869400	-0.29342700
	N	-2.80873000	-1.83553000	0.80242600
	O	-1.67591500	1.59140900	-1.10174200
	C	0.40868200	-0.02341200	0.18888300
	C	0.84458300	1.30228300	0.71831500
	O	0.11492100	2.12110700	1.24289100
	N	1.24107200	-0.94520800	-0.17409400
	N	2.20716800	1.51007900	0.60793600
	H	2.53391500	2.40049000	0.95562000
	C	2.59670500	-0.69790200	-0.16903100
	C	3.11330200	0.55708300	0.20119400
	C	4.48916100	0.79775100	0.16396500
	H	4.86582100	1.77225600	0.45111100

	C	5.36742000	-0.19920700	-0.23188500
	C	4.85020600	-1.45549200	-0.59607700
	C	3.49414200	-1.69814800	-0.56737900
	H	3.08546400	-2.65768900	-0.85213300
	H	5.52998000	-2.23869000	-0.90544800
	C	6.85065900	0.05117800	-0.27983600
	H	7.10221900	1.04569400	0.08538500
	H	7.39150400	-0.67822800	0.32608100
	H	7.22877900	-0.03723900	-1.30060500
Energy:	-1024.87843303 a.u.			
	C	6.17859600	-0.93999700	0.12449900
	C	5.16714100	-1.60690100	-0.49748800
	C	3.82447000	-1.15177900	-0.41207500
	C	4.61305400	0.66058200	0.96441900
	C	5.89470400	0.22690400	0.87422800
	H	7.19565300	-1.29922900	0.04871700
	H	5.32965000	-2.50120400	-1.07913200
	C	2.24445100	0.54365500	0.50800000
	H	4.29130100	1.52868200	1.51659200
	H	6.67764900	0.77469100	1.37585100
	C	1.26247000	-0.16361600	-0.25450700
	C	1.61737900	-1.31341500	-0.93011400
	H	0.84314100	-1.86735700	-1.44789800
	N	3.59173100	-0.00361400	0.33353700
	N	2.85303300	-1.81357900	-1.02235600
**9f**	O	2.08643800	1.46935600	1.27606700
	C	-0.13172700	0.30448600	-0.25142200
	C	-0.38269100	1.73896300	-0.59200200
	O	0.45893100	2.51947400	-0.98859500
	N	-1.08343000	-0.54743100	-0.04669600
	N	-1.71055400	2.10277000	-0.46638600
	H	-1.91322600	3.06769900	-0.68614500
	C	-2.39824200	-0.13058200	-0.03998700
	C	-2.74071500	1.22298600	-0.22203100
	C	-4.07758200	1.62198400	-0.16650300
	H	-4.33776800	2.66358700	-0.30338400
	C	-5.06801200	0.68420100	0.05911700
	H	-6.10437300	0.98449300	0.10149600
	C	-4.72694800	-0.65893600	0.23221100
	C	-3.41194600	-1.07006100	0.18774500
	H	-3.13563700	-2.10379000	0.32982400
	Cl	-5.99713000	-1.83153400	0.51488600
Energy:	-1445.17656399 a.u.			
	C	6.35985800	-0.40849900	0.12410100
	C	5.43287100	-1.27494400	-0.37025900
**9f’**	C	4.04404400	-0.98586300	-0.29847500
	C	4.61505000	1.07985200	0.80134400
	C	5.94026900	0.80293100	0.72483900

	H	7.41346100	-0.64330000	0.06070600
	H	5.70008800	-2.21124700	-0.83547900
	C	2.27414800	0.60617900	0.44365200
	H	4.19311300	1.96662300	1.24589700
	H	6.65431900	1.50694800	1.12382300
	C	1.37896900	-0.31504200	-0.18558900
	C	1.86780500	-1.49152900	-0.71508200
	H	1.16289800	-2.20276700	-1.12939800
	N	3.67752400	0.21382700	0.29783400
	N	3.15616300	-1.84088000	-0.78233200
	O	2.01070800	1.60079200	1.08656100
	C	-0.06365600	-0.02754200	-0.20095900
	C	-0.49983600	1.30066800	-0.72869200
	O	0.22887900	2.11963100	-1.25050300
	N	-0.89770000	-0.95215400	0.15024800
	N	-1.86414200	1.50932800	-0.62076900
	H	-2.18922900	2.40124600	-0.96636300
	C	-2.25372000	-0.70716600	0.13741500
	C	-2.76998000	0.55255800	-0.22652600
	C	-4.14453000	0.79481500	-0.19593000
	H	-4.54176700	1.76125600	-0.47223100
	C	-4.98945200	-0.22832700	0.19062600
	C	-4.50713900	-1.48836200	0.55126800
	C	-3.14588900	-1.71474900	0.52347900
	H	-2.73441800	-2.67425800	0.80299300
	H	-5.19618100	-2.26400900	0.84841200
	Cl	-6.71263400	0.06561200	0.22747300

Energy: -1445.17745639 a.u.

	C	5.45313000	0.22385900	0.73910000
	C	3.51841900	-0.89694000	-0.34248600
	C	4.28764900	0.82654800	1.01005400
	H	6.42974200	0.51382200	1.08320400
	C	1.84524600	0.65861900	0.56933200
	H	4.09843600	1.69643100	1.61439900
	C	0.92712300	-0.13359300	-0.21310500
	C	1.37652300	-1.23439000	-0.90113600
	H	0.66125300	-1.84199300	-1.44061200
	N	3.20209100	0.20833100	0.40246300
**10e**	N	2.66603500	-1.63984200	-0.98367600
	O	1.61216800	1.56989700	1.33474600
	C	-0.50364900	0.22272300	-0.21700600
	C	-0.85847000	1.62178900	-0.60116100
	O	-0.07035700	2.45051100	-1.01430400
	N	-1.38494900	-0.69009600	0.02778900
	N	-2.20853800	1.88753900	-0.49301400
	H	-2.48357500	2.82689300	-0.74275400
	C	-2.72934600	-0.37396000	0.02835500
	C	-3.17044300	0.94211100	-0.20887500
	C	-4.53217700	1.23639400	-0.16296800

	H	-4.87296600	2.24836900	-0.34176600
	C	-5.43986600	0.22730000	0.10869200
	H	-6.49507700	0.46665000	0.13990300
	C	-5.03034000	-1.09309900	0.34367900
	C	-3.67251500	-1.37050700	0.30057100
	H	-3.29929400	-2.36996000	0.48229600
	C	-6.03410400	-2.17846800	0.63786600
	H	-5.98675700	-2.97514900	-0.10703100
	H	-5.84962400	-2.63624400	1.61157800
	H	-7.05059700	-1.78713600	0.64178800
	S	5.24802600	-1.17104900	-0.29531000
Energy:	-1345.65016193 a.u.			

10e’	C	3.63231500	-0.81261700	-0.27931700
	C	1.86144900	0.69937100	0.51367900
	C	0.99389400	-0.22727300	-0.17295400
	C	1.51407700	-1.35353400	-0.76267700
	H	0.83857400	-2.05937500	-1.22881500
	N	3.24539600	0.33340600	0.36387600
	N	2.82877100	-1.67236700	-0.83091000
	O	1.57213800	1.66089600	1.19383800
	C	-0.45910100	0.02345100	-0.18363100
	C	-0.92188800	1.34748800	-0.69158900
	O	-0.20556500	2.19276200	-1.19213100
	N	-1.26915300	-0.92317300	0.16246600
	N	-2.28971000	1.52032000	-0.58961800
	H	-2.63568200	2.40921600	-0.92235900
	C	-2.63064600	-0.70747700	0.15437200
	C	-3.17473400	0.53999300	-0.20158300
	C	-4.55616200	0.74707000	-0.16873700
	H	-4.95493100	1.71598400	-0.44466300
	C	-5.41210300	-0.27596600	0.20899000
	C	-4.86704700	-1.52458100	0.55874500
	C	-3.50549000	-1.73442600	0.53413400
	H	-3.07537200	-2.68783300	0.80759100
	H	-5.52961900	-2.32785700	0.85345700
	C	-6.90079500	-0.06054900	0.25556700
	H	-7.17680600	0.92171900	-0.12479300
	H	-7.42502100	-0.81267600	-0.33672900
	H	-7.27455300	-0.14102900	1.27873400
	S	5.37823500	-0.95604800	-0.24019700
	C	4.29046400	1.08305800	0.88899800
	H	4.04573800	1.99075100	1.41237800
	C	5.49340700	0.54234000	0.65340600
	H	6.45036600	0.93321300	0.94957500
Energy:	-1345.65136864 a.u.			

10f	C	3.77139100	-0.97752000	-0.35449300
	C	2.17655200	0.64365200	0.58136900
	C	1.22527400	-0.07645200	-0.23075300

C	1.62093700	-1.18380600	-0.94299700
H	0.87930000	-1.73732200	-1.50463200
N	3.50811500	0.12375100	0.41596600
N	2.88611800	-1.65581200	-1.02399600
O	1.98428000	1.54548100	1.36867400
C	-0.18280400	0.35506700	-0.23732400
C	-0.46148900	1.78690400	-0.56381200
O	0.36927800	2.58920600	-0.93950900
N	-1.11446600	-0.52013200	-0.04540900
N	-1.79820900	2.11949800	-0.44968600
H	-2.02054000	3.08230500	-0.65979900
C	-2.43882600	-0.13246800	-0.04502800
C	-2.80985000	1.21430300	-0.22051700
C	-4.15587000	1.58248900	-0.17279600
H	-4.43870300	2.61884100	-0.30432400
C	-5.12628100	0.62094200	0.03858800
H	-6.16944800	0.89754700	0.07495000
C	-4.75664700	-0.71545100	0.20575400
C	-3.43232300	-1.09626300	0.16869900
H	-3.13357900	-2.12440200	0.30584900
Cl	-6.00190600	-1.91781500	0.47100000
S	5.48103100	-1.34895400	-0.29750700
C	4.61921800	0.66468000	1.05066700
H	4.47182100	1.52763600	1.67624700
C	5.75208700	0.00443100	0.77542000
H	6.73947600	0.22983600	1.13670800

Energy: -1765.94932553 a.u.

10f’

C	3.97305000	-0.81643400	-0.26577000
C	2.20143600	0.70417100	0.50731900
C	1.33551200	-0.22700700	-0.17536600
C	1.85642100	-1.36012900	-0.75336400
H	1.18166900	-2.06887600	-1.21625600
N	3.58517800	0.33507800	0.36640900
N	3.16974000	-1.68008000	-0.81394400
O	1.90968100	1.67058700	1.17905900
C	-0.11578200	0.02512100	-0.19393300
C	-0.57592800	1.35296700	-0.69970600
O	0.14133100	2.19618100	-1.19828800
N	-0.92957900	-0.92189000	0.14123900
N	-1.94483200	1.53039600	-0.59901500
H	-2.28688300	2.42153400	-0.93016400
C	-2.29078600	-0.70483000	0.12622700
C	-2.83172700	0.54852200	-0.22342000
C	-4.21126400	0.76092000	-0.19651900
H	-4.62794000	1.72222900	-0.46184400
C	-5.03586200	-0.28502800	0.17240600
C	-4.52849300	-1.53928500	0.51908700
C	-3.16268800	-1.73625300	0.49494500
H	-2.73197400	-2.69032100	0.76385800

	H	-5.20226600	-2.33305500	0.80307000
	Cl	-6.76462700	-0.02805300	0.20504100
	S	5.71691900	-0.96268000	-0.21892700
	C	4.62924200	1.08743200	0.88965400
	H	4.38424200	1.99983200	1.40470300
	C	5.83199200	0.54251200	0.66270100
	H	6.78872300	0.93369000	0.95919800
Energy:	-1765.95024156 a.u.			

	C	1.61732400	-1.55356500	-0.38847300
	C	0.20923000	0.37069800	0.18236000
	C	-0.86441100	-0.53198300	-0.17543500
	C	-0.59025300	-1.83246900	-0.52439800
	H	-1.41692100	-2.49783000	-0.73831300
	N	0.64361800	-2.38057000	-0.64202500
	O	0.13446900	1.49439500	0.63730400
	C	-2.25556500	-0.05284800	-0.10713400
	C	-2.59640800	1.18479000	-0.84452300
	C	-4.45127600	-0.35721600	0.61022500
	C	-4.82995400	0.82209100	0.05674300
	H	-5.83211500	1.21933100	0.12127600
	O	-1.86718900	1.80620700	-1.59577600
	N	-3.14372100	-0.76490700	0.52376200
	N	-3.91458400	1.56851700	-0.62862700
	H	-4.19365900	2.41839400	-1.09630600
**11b**	C	-5.39225000	-1.25241400	1.35897600
	H	-5.44504400	-2.23530100	0.88708400
	H	-5.04445300	-1.40591500	2.38178200
	H	-6.39796500	-0.83493500	1.39502700
	C	5.04883700	-0.48793600	0.00634200
	H	5.76267700	-1.26902000	-0.21479600
	C	3.68377300	-0.70598300	-0.08826600
	C	2.75984700	0.31122200	0.19769100
	C	3.17112300	1.57581700	0.58797200
	H	2.44595900	2.34276700	0.80470800
	C	4.54272000	1.79187500	0.68335100
	H	4.90174700	2.76570500	0.98533000
	C	5.46275100	0.78111600	0.39850300
	H	6.52093200	0.98647100	0.48335800
	N	2.95193700	-1.83123100	-0.44303000
	H	3.30468200	-2.73599200	-0.69961700
	N	1.48603500	-0.24004500	-0.00513200
Energy:	-1002.81312197 a.u.			

	C	1.79863900	-1.58636500	-0.19800000
	C	0.24800400	0.26780300	0.21347500
**11b’**	C	-0.75465400	-0.75336500	-0.00375400
	C	-0.38009500	-2.05722400	-0.22171300
	H	-1.15270700	-2.80742900	-0.33166900
	N	0.89261300	-2.51276900	-0.32463600

	O	0.09088700	1.42618300	0.54424700
	C	-2.17923700	-0.38653600	0.07035100
	C	-2.65525900	0.72914300	-0.77427700
	C	-4.31010500	-0.77943600	0.88472100
	C	-4.84260400	0.29354200	0.24050200
	O	-2.01526000	1.32962900	-1.61683700
	N	-2.98626000	-1.09717300	0.80466200
	N	-4.00027400	1.02538800	-0.54835300
	H	-4.35658700	1.79695800	-1.09526600
	C	5.13611400	-0.20465100	-0.05715000
	H	5.90753300	-0.94429100	-0.21975800
	C	3.79276600	-0.54384800	-0.07166200
	C	2.79369000	0.41971500	0.13760100
	C	3.10629400	1.74985300	0.36927500
	H	2.32459500	2.47392900	0.52961300
	C	4.45640700	2.08780500	0.38389100
	H	4.73919200	3.11581700	0.56183600
	C	5.45092600	1.13053400	0.17484500
	H	6.48957700	1.43058800	0.19317300
	N	3.15037600	-1.75789700	-0.27291600
	H	3.57190900	-2.65327700	-0.44435800
	N	1.56646400	-0.25337700	0.04559300
	H	-4.92546000	-1.40606900	1.51361200
	C	-6.26853300	0.73008900	0.33162500
	H	-6.34458700	1.73573200	0.75204400
	H	-6.73952200	0.74778800	-0.65422000
	H	-6.83351800	0.05221700	0.96689100
Energy:	-1002.81542190 a.u.			

	N	-4.00821000	1.81934300	0.55830900
	H	-4.36489500	2.69146700	0.90638700
	O	-1.17013600	-1.40595200	-0.74773400
	N	-2.53884200	0.26347900	0.01855300
	N	2.75548600	-1.77482500	0.81440900
	H	2.96727600	-2.67747800	1.21517900
	N	-1.71206800	2.28549300	0.98143800
	N	2.12067700	0.74008800	-0.11008500
	O	0.63211300	-2.01846600	1.58031200
	C	6.82816400	1.61518400	-1.26854500
**11e**	H	6.42262400	2.57966500	-1.57038600
	H	7.60664400	1.79801900	-0.52468500
	H	7.31389700	1.16989500	-2.13943700
	C	-6.49268100	-0.60849700	-0.77202500
	H	-7.54547200	-0.77782600	-0.95107900
	C	-5.56968000	-1.59552100	-1.12303100
	H	-5.92111300	-2.51502500	-1.56956700
	C	-4.20474200	-1.42383600	-0.91228300
	H	-3.47766700	-2.17326900	-1.17831400
	C	-3.80331800	-0.22828800	-0.33762400
	C	-4.73009400	0.76516800	0.01460600

	C	-6.08861600	0.59153400	-0.19585900
	H	-6.80479900	1.35427000	0.07587100
	C	-2.67766200	1.52000000	0.55827100
	C	-0.20300300	0.47660900	0.38526800
	C	-0.48149100	1.72896500	0.87822600
	H	0.33763200	2.34173300	1.23242100
	C	1.18300700	-0.02055200	0.35430300
	C	3.76922200	-0.99487400	0.30247500
	C	3.42698700	0.29513100	-0.13859300
	C	4.43179500	1.13069400	-0.64573600
	H	4.13546600	2.11585200	-0.98046800
	C	5.74961600	0.71581200	-0.72327700
	C	6.06109000	-0.58103900	-0.28043200
	H	7.08567600	-0.92723400	-0.33506100
	C	5.09428300	-1.42917400	0.22392600
	H	5.35660400	-2.42420100	0.56089100
	C	-1.26112300	-0.34818400	-0.15809300
	C	1.44708200	-1.35839300	0.96496800
Energy:	-1156.51527369 a.u.			
	N	-4.14957800	1.75339900	0.36655100
	H	-4.55576400	2.63930400	0.60974800
	O	-1.13256700	-1.43486000	-0.58127700
	N	-2.59273000	0.23359500	-0.00512900
	N	2.83847000	-1.35552900	0.91460800
	H	3.11449600	-2.18329600	1.42349800
	N	-1.88167800	2.40739700	0.67901700
	N	2.02215000	0.96700300	-0.31615300
	O	0.75096900	-1.62824000	1.76375900
	C	-6.49419600	-0.96613400	-0.60444000
	H	-7.53646800	-1.21906400	-0.74151300
	C	-5.51600600	-1.93218200	-0.84778500
	H	-5.81493500	-2.91961600	-1.17032000
	C	-4.16198500	-1.65435900	-0.68523200
**11e’**	H	-3.39310800	-2.38645000	-0.86944300
	C	-3.82847300	-0.37455000	-0.27098600
	C	-4.81089500	0.59767000	-0.02680900
	C	-6.15838300	0.31821300	-0.18847500
	H	-6.91704000	1.06482500	0.00012600
	C	-2.80282300	1.53764700	0.37590100
	C	-0.26987300	0.63341300	0.27904300
	C	-0.61969900	1.91827900	0.61814900
	H	0.16404800	2.62002600	0.87348800
	C	1.14453400	0.22141300	0.27335900
	C	3.78931100	-0.59381800	0.27384200
	C	3.35149600	0.60111700	-0.32491600
	C	4.29686200	1.40979300	-0.96995100
	H	3.94780200	2.32334700	-1.43085600
	C	5.62396900	1.04091500	-1.01596800
	C	6.06218700	-0.15581700	-0.42153500

	C	5.13552800	-0.96442700	0.21856200
	H	5.45120400	-1.89106200	0.68313300
	C	-1.28177700	-0.31689500	-0.13053600
	C	1.50736300	-1.00402300	1.04523800
	C	7.51575800	-0.54168900	-0.48008900
	H	7.87217500	-0.57198400	-1.51145900
	H	8.13496100	0.18434300	0.05131500
	H	7.69026200	-1.52000900	-0.03478000
	H	6.34133000	1.67597300	-1.51928600
Energy:	-1156.51611363 a.u.			
	N	-1.97191400	1.73769500	-0.49981000
	N	-2.51326800	-0.50470900	0.07274800
	C	-4.74886600	-1.28721600	-0.11158500
	H	-5.45999900	-2.09136000	-0.00082700
	C	-3.44376900	-1.49995000	0.19893700
	H	-3.03885300	-2.43217400	0.55860600
	C	-5.14720800	-0.01260400	-0.57281400
	H	-6.18256600	0.17272000	-0.82366200
	C	-4.22140300	0.98115100	-0.69860000
	H	-4.47211700	1.97218300	-1.04408300
	C	-2.86049600	0.75575100	-0.37303200
	C	-1.11579300	-0.85475900	0.44263300
	O	-0.91009900	-1.98197600	0.82056400
	C	3.80999600	0.13827200	-0.94595600
	H	2.99453800	-0.51552500	-1.24649200
Amide (derived from ethylenediamine A)	H	4.25689000	0.53471700	-1.85865300
	C	4.85863100	-0.66986700	-0.17981700
	H	4.41774600	-0.99700400	0.76673100
	H	5.70474100	-0.02004100	0.05620700
	O	1.72228100	-0.99356100	0.87760000
	O	1.81108400	2.30215200	1.20099300
	C	1.21659400	0.06710100	0.56608700
	C	2.12062400	1.32066900	0.54074200
	C	-0.21579900	0.25471900	0.28274900
	C	-0.71127500	1.47375300	-0.17338800
	H	-0.02826700	2.30643100	-0.27005300
	N	5.34359200	-1.75876500	-1.03068600
	H	6.18423700	-2.16583900	-0.64133000
	H	4.65647400	-2.50303800	-1.06814600
	N	3.26783600	1.24828900	-0.17298100
	H	3.84990400	2.06631700	-0.06219800
Energy:	-909.51096090 a.u.			
	N	-2.15175200	1.75233300	0.02307400
Imine (derived from ethylenediamine A)	N	-2.75359000	-0.54282600	-0.01860500
	C	-5.04426900	-1.18198000	-0.01962100
	H	-5.78588000	-1.96600100	-0.02819400
	C	-3.72732600	-1.50813800	-0.02778000
	H	-3.34087300	-2.51429400	-0.04181300

	C	-5.41792000	0.18267500	0.00035400
	H	-6.46376500	0.45729600	0.00675000
	C	-4.45461300	1.14547900	0.01238300
	H	-4.68376800	2.19988000	0.02966600
	C	-3.07653000	0.80400600	0.00488000
	C	-0.40568700	0.05996500	-0.03665300
	C	-0.87638500	1.36120300	0.00698100
	H	-0.15958600	2.17415100	0.01873400
	C	-1.35345200	-1.02303300	-0.03326600
	O	-1.17416200	-2.21779300	-0.02266200
	C	1.04360000	-0.20033900	-0.07531000
	C	1.93915500	0.94790400	0.39164700
	O	2.15479400	1.19818800	1.55000500
	C	2.95057200	-1.57085900	-0.45108500
	H	3.28450200	-1.56964600	-1.49218300
	H	3.54727700	-0.81807900	0.08193500
	C	3.23663500	-2.95249200	0.13591000
	H	2.83642900	-2.99241700	1.15703000
	H	2.68848100	-3.69211700	-0.44767000
	O	2.47956400	1.60198200	-0.64966800
	C	3.41879900	2.67004200	-0.34971300
	H	4.07800300	2.69315000	-1.21452500
	H	3.98906900	2.39289500	0.53436200
	C	2.70884500	3.99515400	-0.15610000
	H	3.44599700	4.78653500	-0.01094400
	H	2.10829100	4.24666500	-1.03029500
	H	2.06489400	3.96853000	0.72164800
	N	1.52583600	-1.31730300	-0.43318900
	N	4.66928900	-3.24774700	0.04159000
	H	5.18927300	-2.69663100	0.71489200
	H	4.84305900	-4.21764900	0.27335400
Energy:	-988.14381545 a.u.			
	N	2.02899200	-0.44355900	-0.02780500
	C	3.95880900	-1.82728400	-0.07955600
	H	4.39259300	-2.80724500	-0.20623800
	C	2.61357200	-1.67420800	-0.17688700
	H	1.91345900	-2.47048700	-0.37159300
	C	4.76383800	-0.69704100	0.18804700
	H	5.83642700	-0.80531700	0.27068200
Amide (derived from acetamidine B)	C	4.18388500	0.52791300	0.34180300
	H	4.75368600	1.42082100	0.54748800
	C	2.78078700	0.69143900	0.23474600
	C	0.05922900	0.94099100	-0.03869800
	C	0.91684800	1.99269400	0.24881400
	H	0.48842400	2.98162000	0.36189800
	C	0.56934100	-0.38452600	-0.14966600
	O	-0.04436300	-1.42351000	-0.29810900
	C	-1.37909300	1.20180200	-0.20504000
	C	-2.18159100	0.25180000	-1.10425800

	O	-1.91862300	2.22260300	0.17443200
	O	-1.88642600	0.26868200	-2.28347100
	C	-2.92983700	-0.37490700	1.81990200
	H	-1.90036400	-0.72037600	1.71595900
	H	-2.91865500	0.70629100	1.94869100
	H	-3.37637300	-0.83474800	2.69565500
	N	-4.82808300	-1.42363600	0.80120200
	H	-5.30309000	-1.63046600	-0.07751100
	N	2.23482100	1.89605600	0.38582600
	N	-3.28269400	-0.42876100	-0.65334700
	H	-3.79098000	-0.84945200	-1.42103000
	C	-3.75096900	-0.77092900	0.62946600
Energy:	-908.30560559 a.u.			

Imine (derived from acetamidine B)	N	2.00277000	1.67467600	-0.83337000
	N	2.74253800	-0.33934800	0.18557100
	C	5.05689100	-0.69924300	0.59167400
	H	5.84171000	-1.33550700	0.97099700
	C	3.76752000	-1.11753800	0.65742700
	H	3.44486200	-2.06005800	1.06919800
	C	5.34494000	0.56436600	0.02575700
	H	6.36773800	0.90999300	-0.03370900
	C	4.32854100	1.34186400	-0.44316100
	H	4.49247200	2.31323000	-0.88366500
	C	2.98100800	0.90414800	-0.37412700
	C	1.38015500	-0.90251500	0.30847200
	O	1.26524600	-1.98351200	0.83510900
	C	-2.06735500	0.69457500	-0.45285400
	O	-2.72376400	0.79624800	-1.45748100
	N	-1.42140500	-1.61910200	-0.09933200
	O	-2.14765500	1.49862900	0.61397300
	C	-3.14763300	2.55743700	0.57731500
	H	-3.25360800	2.89667400	-0.45064800
	H	-2.71497800	3.35212200	1.18100300
	C	-4.46720100	2.07537700	1.14444700
	H	-5.18496600	2.89738300	1.14191200
	H	-4.87478500	1.26106200	0.54810100
	H	-4.34738800	1.72919700	2.17056900
	C	-3.25596800	-2.69421000	-1.29075100
	H	-3.24349200	-1.99947900	-2.13216900
	H	-2.59636000	-3.52658600	-1.53978300
	H	-4.26665800	-3.05878700	-1.12933100
	N	-3.51107700	-1.89057200	0.97872300
	H	-3.00902600	-1.42525400	1.73521700
	C	-2.76875200	-2.00443000	-0.05003200
	C	-1.03782000	-0.41461800	-0.23220100
	C	0.76097000	1.20294100	-0.74629500
	H	-0.00072100	1.86329300	-1.14608100
	C	0.37550400	-0.02278400	-0.22493000
Energy:	-986.94091849 a.u.			

	O	1.15325700	-0.39557900	0.00005600
	H	1.98624900	0.08318000	-0.00011600
	C	0.08233900	0.54746300	-0.00002300
	H	0.13664200	1.19307700	-0.88455900
Ethanol	H	0.13660200	1.19317400	0.88444600
	C	-1.22163600	-0.22292000	-0.00001000
	H	-2.06996200	0.46285600	-0.00007300
	H	-1.28987900	-0.85751100	-0.88358200
	H	-1.28993000	-0.85740500	0.88363500
Energy:	-155.10598089 a.u.			
	O	0.00000000	0.00000000	0.11772300
Water	H	0.00000000	0.75999600	-0.47089200
	H	0.00000000	-0.75999600	-0.47089200
Energy:	-76.45983966 a.u.			

6. References

1. Bruker, APEX2 (Version 2.1), COSMO (Version 1.56), BIS (Version 2.0.1.9), SAINT (Version 7.3A) and SADABS (Version 2004/1) & XPREP (Version 2005/4), Bruker AXS Inc., Madison, Wisconsin, USA, **2006.**

2. G.M. Sheldrick, SHELXS-97 and SHELXL-97, University of Göttingen, Germany,**1997**.

3. Coppens, P.; Leiserowitz, L.; Rabinovich, D. *Acta Crystallogr.* **1965**, *18*, 1035-1038.

4. Stewart, J. J. P. *Comput. Chem.***1989**, *10*, 209-220.

5. J. J. P. Stewart, *J. Comput. Chem.***1989**, *10*, 221-264.

6. Frisch, M. J.; Trucks, G. W.; Schlegel, H. B.; Scuseria, G. E.; Robb, M. A.; Cheeseman, J. R.; Scalmani, G.; Barone, V.; Mennucci, B.; Petersson, G. A.; Nakatsuji, H.; Caricato, M.; Li, X.; Hratchian, H. P.; Izmaylov, A. F.; Bloino, J.; Zheng, G.; Sonnenberg, J. L.; Hada, M.; Ehara, M.; Toyota, K.; Fukuda, R.; Hasegawa, J.; Ishida, M.; Nakajima, T.; Honda, Y.; Kitao, O.; Nakai, H.; Vreven, T.; Montgomery, J. A., Jr.; Peralta, J. E.; Ogliaro, F.; Bearpark, M.; Heyd, J. J.; Brothers, E.; Kudin, K. N.; Staroverov, V. N.; Kobayashi, R.; Normand, J.; Raghavachari, K.; Rendell, A.; Burant,

J. C.; Iyengar, S. S.; Tomasi, J.; Cossi, M.; Rega, N.; Millam, J. M.; Klene, M.; Knox, J. E.; Cross, J. B.; Bakken, V.; Adamo, C.; Jaramillo, J.; Gomperts, R.; Stratmann, R. E.; Yazyev, O.; Austin, A. J.; Cammi, R.; Pomelli, C.; Ochterski, J. W.; Martin, R. L.; Morokuma, K.; Zakrzewski, V. G.; Voth, G. A.; Salvador, P.; Dannenberg, J. J.; Dapprich, S.; Daniels, A. D.; Farkas, Ö.; Foresman, J. B.; Ortiz, J. V.; Cioslowski, J.; Fox, D. J. Gaussian, Inc., Wallingford CT, **2009**.
